# Prevalence of psychotic disorders and its association with methodological issues. A systematic review and meta-analyses

**DOI:** 10.1371/journal.pone.0195687

**Published:** 2018-04-12

**Authors:** Berta Moreno-Küstner, Carlos Martín, Loly Pastor

**Affiliations:** 1 Department of Personality, Assessment and Psychological Treatment, University of Malaga, Málaga, Spain; 2 Biomedical Research Institute of Malaga (IBIMA), Andalusian Group of Psychosocial Research, Maristán Network, Málaga, Spain; 3 Andalusian Health Service, North East Granada Sanitary District, Clinical Management Unit at Marquesado, Alquife, Granada, Spain; FIDMAG (Fundación para la Investigación y la Docencia Maria Angustias Giménez), Barcelona, and CIBERSAM (Centro de Investigación Biomédica en Red de Salud Mental), SPAIN

## Abstract

**Objectives:**

The purpose of this study is to provide an updated systematic review to identify studies describing the prevalence of psychosis in order to explore methodological factors that could account for the variation in prevalence estimates.

**Methods:**

Studies with original data related to the prevalence of psychosis (published between 1990 and 2015) were identified via searching electronic databases and reviewing manual citations. Prevalence estimates were sorted according to prevalence type (point, 12-months and lifetime). The independent association between key methodological variables and the mean effect of prevalence was examined (prevalence type, case-finding setting, method of confirming diagnosis, international classification of diseases, diagnosis category, and study quality) by meta-analytical techniques and random-effects meta-regression.

**Results:**

Seventy-three primary studies were included, providing a total of 101 estimates of prevalence rates of psychosis. Across these studies, the pooled median point and 12-month prevalence for persons was 3.89 and 4.03 per 1000 respectively; and the median lifetime prevalence was 7.49 per 1000. The result of the random-effects meta-regression analysis revealed a significant effect for the prevalence type, with higher rates of lifetime prevalence than 12-month prevalence (p<0.001). Studies conducted in the general population presented higher prevalence rates than those carried out in populations attended in health/social services (p = 0.006). Compared to the diagnosis of schizophrenia only, prevalence rates were higher in the probable psychotic disorder (p = 0.022) and non-affective psychosis (p = 0.009). Finally, a higher study quality is associated with a lower estimated prevalence of psychotic disorders (p<0.001).

**Conclusions:**

This systematic review provides a comprehensive comparison of methodologies used in studies of the prevalence of psychosis, which can provide insightful information for future epidemiological studies in adopting the most relevant methodological approach.

## Introduction

### 1.1 The burden of schizophrenia and related disorders

There is no doubt that schizophrenia is a chronic and severe mental illness that implies a high global disease burden and was ranked among the top 15 leading causes of disability worldwide in 2016 [1]. Although schizophrenia is classified as a *low-prevalence disorder* [2], its economic burden was estimated to range from 0.02% to 1.65% of gross domestic product [3].

To understand the causes of mental disorders, it is essential to measure their frequency [4]. While incidence studies are useful for identifying disease risk factors, prevalence studies show the burden of disease in society [5]. Knowing the exact extent of disease burden in populations, at local, national or international levels is important to advise governments, health managers, and health professionals to improve development and distribution of health services [6,7].

In addition to etiological and environmental factors, the variability of the estimated prevalence may also be influenced by the methodological factors of the studies [2]. There is a growing consensus that the variability of the prevalence of schizophrenia in epidemiological studies is due partly to the differences in the methodological aspects of the studies [8] [9], but there are also other factors such as genetic and environmental that do play a role in this variation.

Several studies in the last three decades have published original data on the prevalence of schizophrenia which has been summarized in three systematic reviews [5,10,11]. However, one of the main problems found in previous systematic reviews is the high heterogeneity in the methodological aspects of the studies, which hinders the comparison of results in a homogeneous way [9,12]. In two systematic reviews of prevalence of schizophrenia, Saha et al.[10] and Simeone et al. [11] analyzed factors such as diagnostic criteria, case selection methods, and study quality but stated that findings were inconclusive. To our knowledge, none of the previous systematic reviews has performed a meta-analysis including the methodological aspects of the studies.

### 1.2 The present study

The first objective of this study is to develop a comprehensive, systematic review of the existing evidence from the past 25 years (1990–2015) regarding studies that assess the prevalence of psychotic disorders worldwide in order to explore possible sources of heterogeneity in the data by sorting the results according to methodological features. The second objective is to apply a meta-analysis to determine whether the exhibited variation in the prevalence estimate is associated with methodological factors including the following: 1) prevalence type, 2) case-finding setting, 3) method of confirming diagnosis, 4) international classification of diseases, 5) diagnosis category and 6) study quality.

## Methods

### 2.1 Terminology

In this review, we refer to a “citation/reference” as any unique article from the published literature included in our analyses. We distinguish this from a “study”, which refers to any of the different studies performed in an article. Thus, it is important to highlight that one citation can include more than one study and it may generate many items of information on the estimates of the prevalence of psychotic disorders. For example, one citation can offer different estimates concerning the type of prevalence, such as point, 12-month and lifetime, or it may also offer estimates for different locations, such as Sao Paulo, Porto Alegre, or Brasilia within the same citation.

For the preparation of this manuscript, we adhered to the PRISMA statement [13] and included a copy of the PRISMA checklist (S1 Appendix). This is a proposal to improve systematic reviews and meta-analyses publications consisting of a 27-item instrument that follows the basic structure of a scientific article and details the specific contents that should be reported in each section. We also followed the MOOSE criteria which is another formal reporting guideline for meta-analyses of observational studies. However, we have attached a copy only of the PRISMA checklist as do the majority of the meta-analysis we have revised. This review has been registered in PROSPERO, with registration number CRD42016047069.

### 2.2 Literature search: Identification of articles

To identify relevant articles, we conducted a comprehensive systematic search of the different electronic bibliographic indexes of published literature: Medline, Psycinfo, Scopus, ProQuest Psychology Journals, Embase and Web of Science, with January 1, 1990 to December 31, 2015 as the reference period. Keywords used for this research were: "schizophreni*”, *“psychosis”*, “epidemiolog*” and *“prevalence”*. Specifically, the keyword combination introduced was as follows: Title (schizophreni* OR *psychosis*) AND Title (*prevalence* OR epidemiolog*). Searches were carried out between October 2015 and February 2016.

To minimize the possibility of overlooking any relevant data, we also reviewed the bibliographies of each citation identified above, as well as the systematic literature reviews pertinent to our objectives [5,10,11].

### 2.3 Inclusion criteria

The inclusion criteria for articles in the search were: (i) reported prevalence rates of psychotic disorders, (ii) general population or attended population studies, (iii) original studies with primary data, and (iv) period of publication from the year 1990 to 2015, (v) articles published in English or Spanish.

### 2.4 Selection process articles: Flow diagram

**Fig 1:**
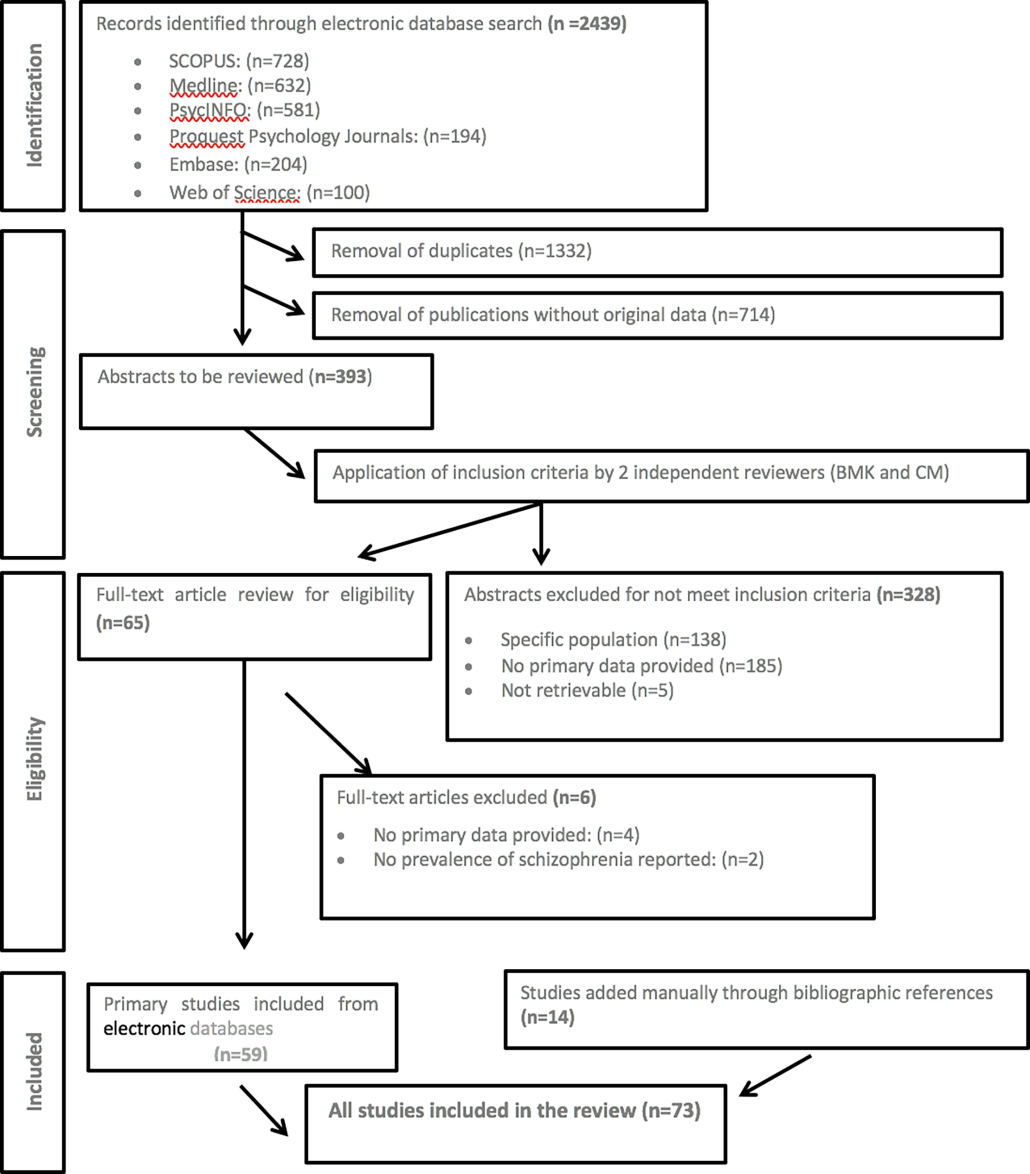
Flow diagram (selection strategy) of included studies from 1990 to 2015.

A flow diagram of the search strategy for study inclusion is shown in Fig 1. At the first stage, we identified 2439 initial citations from the published literature. After identification and removal of duplicate citations (n = 1332) and citations without original data (n = 714), a total of 393 unique citations met potential inclusion for this review. The defined inclusion criteria were applied to the title, and where necessary, abstracts for these citations were revised by two researchers (BMK, CM), independently. The reviewers agreed that 65 citations “met inclusion criteria” and a further 328 citations “did not meet inclusion criteria” for the review. Thus, at the eligibility stage, the full text of the article was obtained for any citation that met inclusion criteria. From the electronic database search, 59 citations met the inclusion criteria for the systematic review following scrutiny of the full text. However, these results were extended through manual bibliography checks of the previously selected articles. With this procedure, 14 additional valid articles were included. In total, the number of articles included in this review rose to 73 (59+14) which provided relevant prevalence data.

### 2.5 Data extraction

All manuscripts were retrieved in paper or electronic format, with all necessary permissions granted prior to their distribution. Once an article was included in this review, the information was extracted in a card for each article. The cards for these articles are available as supporting information in S2 Appendix. Card for each article.

We distinguished the following types of variables:

1. Citation-level variables: article reference number [ID], first author, year of publication and country.
2. Middle-level or meta-variables (methodological issues): 1) case-finding setting (general population/attended in health and/or social services), 2) method of confirming diagnosis (Composite International Diagnostic Interview [CIDI]/Schedules for Clinical Assessment in Neuropsychiatry [SCAN]/Structured Clinical Interview for DSM-IV [SCID]/clinical diagnoses/others), 3) international classification of diseases (International Classification of Diseases [ICD]/ Diagnostic and Statistic Manual of Mental Disorders [DSM], both), 4) diagnostic categories (schizophrenia/non-affective psychosis/schizophrenia and related disorders/probable psychotic disorder, 5) study quality (1–16) and 6) prevalence type (point/12-month/lifetime).
3. Rate-level variables: number of cases (numerator), population size (reported denominator), prevalence rate by 1000 persons.

S1 Table provides descriptions of the variables we used to analyze the studies.

To assess possible bias in prevalence reporting, the study quality was given for certain features following the criteria of Saha et al. [10]. Details of the quality scores used in this review are provided in S2 Table.

### 2.6 Data analysis

Subsequently, data extraction was managed using a Microsoft Excel file where the categories of data to be collected were examined and agreed on beforehand. The document screening was carried out by two independent reviewers (BMK, CM) who checked all data used in the analysis. When disagreements arose, these were resolved through consensus by a third co-author (LPE).

#### 2.6.1 Identification of relevant data

Prevalence estimates were sorted through the application of sequential filters. To classify relevant data in order to identify all citations which included suitable data for any given analysis, we classified the studies by the following: case-finding setting; method of confirming diagnosis; international classification of diseases; diagnosis categories; and study quality. For this classification, many different analyses could be permuted concerning the type of prevalence rate (point/12-month/lifetime). For each, we identified and recorded citations that contributed relevant rate data and extracted this to a separate analysis.

#### 2.6.2 Descriptive analyses

This included descriptive study characteristics reporting the studies counts and percentage. Descriptive statistics, including means, standard deviations, median, ranges, quantiles (10%, 25%, 75%, 90%) and interquartile ranges (IQRs) were used to summarize prevalence estimates. The variation of the estimated rates and any other relevant information (where available) was presented. For each methodological variable we presented the global, point, 12-month and lifetime prevalence rate.

#### 2.6.3 Meta-analyses

First, we performed bivariate analyses. We included all independent variables measured and then excluded (step by step) from the model those variables that obtained a significance at P<0.05 (results not shown).

In the meta-analysis, we estimated the mean effect (mean prevalence) in all studies with a 95% confidence interval (CI). We also estimated the mean prevalence depending on the period of study (point, 12 months and lifetime). We used random-effects meta-regression to estimate the effects of meta-level (methodological) explanatory variables on the outcome of interest. We selected the random effects model for this study assuming that the studies included in the meta-analysis were conducted out in populations that might differ between them. Statistical heterogeneity was evaluated using *I*^2^. In addition, we calculated the *Q* statistic and its *P* value. In this way, we obtained the mean prevalence (unweighted) because we considered all the articles to have the same weight. Thus, we determined which variables were associated independently with the mean effect of the prevalence of psychotic disorders and the proportion of the variance that is explained in the final adjusted model. P<0.05 was set as the limit of statistical significance of the coefficients.

Unless otherwise stated, all prevalence rates are expressed per 1000 persons with their 95% CI where available.

#### 2.6.4 Publication bias

To inspect evidence of publication bias we conducted visual inspection of funnel plots and used Egger’s test for bias in meta-analyses[14].

All statistical analyses were performed with the open-source software environment R 2.12.0 [15]. Calculation of mean effect and random-effects meta-regression was performed with the metaphor package [16].

## Results

### 3.1 Characteristics of the studies

We identified 73 articles which provided original data on the prevalence of psychotic disorders worldwide, between 1990 and 2015 [11,17–88]. Key features of these articles sorted by first author are provided in Table 1.

**Table 1:** Characteristics of the 101 studies included in the systematic review.

ID	First author, year	Country	Case finding setting	Method of confirming diagnosis	International classification	Diagnostic category	Study quality^1^	Prevalence type	Population	Case	Rate^2^	LCI	UCI
17	Agius, 2009	UK (South East)	attended	clinical	DSM	SRD	7	Point	21350	42	1.97	1.42	2.66
17	Agius, 2009	UK (North East)	attended	clinical	DSM	SRD	7	Point	43829	137	3.13	2.62	3.69
17	Agius, 2009	UK (North West)	attended	clinical	DSM	SRD	7	Point	57414	122	2.12	1.76	2.54
17	Agius, 2009	UK (South West)	attended	clinical	DSM	SRD	7	Point	61778	331	5.36	4.80	5.97
18	Almeida, 1997	Porto-Alegre	general population	SCID	DSM	NAP	15	Lifetime	2384	57	24.00	4.36	43.64
18	Almeida, 1997	Sao Paulo	general population	SCID	DSM	NAP	15	Lifetime	1742	16	9.18	4.70	13.66
18	Almeida,1997	Brasilia	general population	SCID	DSM	NAP	15	Lifetime	2345	7	3.24	0.94	5.54
19	Andrade, 2002	Brazil	general population	CIDI	ICD	NAP	13	12-month	1464	12	8.20	3.58	12.82
19	Andrade, 2002	Brazil	general population	CIDI	ICD	NAP	13	Point	1464	10	6.83	2.61	11.05
19	Andrade, 2002	Brazil	general population	clinical	ICD	NAP	13	Lifetime	1464	28	19.13	12.11	26.14
20	Andrews, 2001	Australia	general population	CIDI	both	NAP	15	Point	10641	43	4.04	2.84	5.25
20	Andrews, 2001	Australia	general population	CIDI	both	NAP	15	12-month	10641	43	4.04	2.84	5.25
21	Arajarvi, 2005	Finland	general population	SCID	ICD	Schizophrenia	14	Lifetime	12368	191	15.44	13.27	17.62
22	Awas, 1999	Ethiopia	general population	CIDI	ICD	Schizophrenia	15	Point	10468	63	6.02	4.54	7.50
23	Bijl, 1998	Holland	general population	CIDI	DSM	Schizophrenia	16	12-month	7076	14	1.98	0.94	3.01
23	Bijl, 1998	Holland	general population	CIDI	DSM	Schizophrenia	16	Point	7076	14	1.98	0.94	3.01
23	Bijl, 1998	Holland	general population	CIDI	DSM	Schizophrenia	16	Lifetime	7076	28	3.96	2.49	5.42
24	Binbay, 2012	Turkey	general population	CIDI	DSM	Schizophrenia	13	Lifetime	4011	30	7.48	4.81	10.15
25	Chien, 2004	Taiwan	general population	clinical	ICD	Schizophrenia	12	12-month	137914	607	4.40	4.05	4.75
26	Cho, 2007	Korea	general population	CIDI	DSM	Schizophrenia	13	Lifetime	6275	13	2.07	0.95	3.20
26	Cho,2007	Korea	general population	CIDI	DSM	Schizophrenia	13	12-month	6275	13	2.07	0.95	3.20
27	Clayer, 1995	Australia	general population	others	DSM	Schizophrenia	13	12-month	1009	8	7.93	2.46	13.40
28	Cohidon, 2009	France	general population	others	ICD	Probable	7	Lifetime	40157	1084	26.99	25.41	28.58
30	Díaz Martínez, 2003	Mexico	general population	CIDI	ICD	Schizophrenia	7	12-month	608	12	19.74	8.68	30.79
29	Díaz-Cruz, 2004	Spain	general population	CIDI	DSM	NAP	12	Point	800	2	2.50	-0.96	5.96
31	Dourado, 2001	Azores	Atended	others	both	Schizophrenia	10	Lifetime	4332	14	2.40	0.94	3.86
32	Favarelli, 2004	Italy	general population	SCID	DSM	NAP	14	Lifetime	2363	17	7.19	3.79	10.60
33	Fekadu, 2004	Ethiopia	general population	CIDI	ICD	Schizophrenia	10	Point	2281	1	0.44	-0.42	1.30
34	Fors, 2007	Sweden	Atended	clinical	both	Schizophrenia	10	12-month	64041	237	3.70	3.23	4.17
35	Gigantesco, 2006	Italy	general population	others	ICD	Probable	15	Point	267	1	3.75	-3.58	11.07
35	Gigantesco, 2006	Italy	general population	others	ICD	Probable	15	12-month	267	1	3.75	-3.58	11.07
35	Gigantesco, 2006	Italy	general population	others	ICD	Probable	15	Lifetime	267	2	7.49	-2.85	17.83
36	Goldner, 2003	Canada	Atended	clinical	both	Schizophrenia	8	12-month	11516	48	4.17	2.99	5.34
37	Gureje, 2010	Nigeria	general population	CIDI	DSM	NAP	11	Lifetime	4985	105	21.06	17.08	25.05
37	Gureje, 2010	Nigeria	general population	CIDI	DSM	NAP	11	12-month	4985	55	11.03	8.13	13.93
38	Hosain, 2007	Bangladesh	general population	clinical	DSM	NAP	10	Point	766	9	11.75	4.12	19.38
39	Hovatta, 1997	Finland	Atended	clinical	DSM	SRD	12	Lifetime	4998478	29091	5.82	5.75	5.88
40	Jablensky, 2000	Australia	Atended	SCAN	ICD	Probable	13	Point	980	5	5.10	0.64	9.56
41	Jeffreys, 1997	UK	Atended	clinical	DSM	Probable	14	Point	115294	588	5.10	4.69	5.51
42	Jenkins, 1997	UK	general population	SCAN	ICD	NAP	15	12-month	10108	40	3.96	2.73	5.18
43	Jörgensen, 2013	Sweden	attended	clinical	ICD	Schizophrenia	10	12-month	946381	3502	3.70	3.58	3.82
44	KaKe, 2008	Australia	attended	clinical	ICD	Schizophrenia	10	12-month	3736269	11956	3.20	3.14	3.26
45	Kebede, 1999	Ethiopia	general population	CIDI	ICD	Schizophrenia	12	Point	10203	31	3.04	1.97	4.11
46	Kebede, 2003	Ethiopia	general population	CIDI	ICD	Schizophrenia	11	Lifetime	68378	321	4.69	4.18	5.21
47	Kendler, 1996	USA	general population	CIDI	DSM	SRD	14	Lifetime	8098	89	10.99	8.72	13.26
49	Kessler, 1994	USA	general population	CIDI	DSM	NAP	15	Lifetime	8098	57	7.04	5.22	8.86
49	Kessler, 1994	USA	general population	CIDI	DSM	NAP	15	12-month	8098	40	4.94	3.41	6.47
48	Kessler, 2005	USA	general population	CIDI	DSM	NAP	13	Lifetime	9282	29	3.12	1.99	4.26
48	Kessler, 2005	USA	general population	CIDI	DSM	NAP	13	12-month	9282	46	5.00	3.63	6.60
50	Kodesh, 2012	Israel	attended	clinical	ICD	SRD	8	12-month	8848	44	4.97	3.51	6.44
51	Kringlen, 2001	Norway	general population	CIDI	DSM	NAP	12	Lifetime	2066	8	3.87	1.19	6.55
51	Kringlen, 2001	Norway	general population	CIDI	DSM	NAP	12	12-month	2066	4	1.94	0.04	3.83
52	Kringlen, 2006	Norway	general population	CIDI	DSM	NAP	15	12-month	1080	3	2.78	-0.36	5.92
52	Kringlen, 2006	Norway	general population	CIDI	DSM	NAP	15	Lifetime	1080	4	3.70	0.08	7.33
53	Kurihara, 2005	Indonesia	general population	SCID	DSM	Schizophrenia	11	Point	8546	36	4.21	2.84	5.59
54	Lindström, 1997	Sweden	attended	clinical	DSM	Schizophrenia	9	12-month	64886	273	4.21	3.71	4.71
55	McConnell, 2002	EIRE	general population	SCAN	ICD	Schizophrenia	10	12-month	1242	5	4.03	0.50	7.55
56	McCreadie, 1997	Nishsdale	attended	others	ICD	Schizophrenia	12	Point	57831	161	2.78	2.35	3.21
56	McCreadie, 1997	Norwood	attended	others	ICD	Schizophrenia	12	Point	23007	52	2.24	1.63	2.85
56	McCreadie, 1997	Nunhead	attended	others	ICD	Schizophrenia	12	Point	29448	102	3.46	2.79	4.13
57	Moreno, 2008	Spain	attended	clinical	ICD	SRD	9	12-month	270629	774	2.86	-0.90	6.62
58	Moreno-Küstner, 2015	Spain	attended	clinical	ICD	SRD	11	12-month	265229	1053	3.97	0.84	3.40
59	Morgan, 2014	Australia	attended	others	ICD	NAP	13	12-month	4928	12	3.45	3.73	4.21
59	Morgan, 2014	Australia	attended	others	ICD	NAP	13	Point	4928	10	3.10	1.01	3.73
60	Myles-Worsley, 1999	Micronesia	attended	others	DSM	SRD	10	Lifetime	13750	262	19.90	17.57	22.23
61	Nimgaonkar, 2000	Canada	attended	clinical	DSM	Schizophrenia	9	12-month	8542	11	1.29	0.94	1.64
62	Ortega, 1995	Spain	general population	others	DSM	SRD	14	Point	793	1	1.26	-1.21	3.73
8	Perälä, 2007	Finland	general population	SCID	DSM	Schizophrenia	16	Lifetime	8028	70	8.72	6.69	10.75
63	Perälä, 2008	Finland. North	general population	CIDI	both	Schizophrenia	10	Lifetime	8028	148	18.44	15.49	21.38
63	Perälä, 2008	Finland. South	general population	CIDI	both	Schizophrenia	10	Lifetime	8028	74	9.22	7.13	11.31
63	Perälä, 2008	Finland. East	general population	CIDI	both	Schizophrenia	10	Lifetime	8028	86	10.71	8.46	12.96
63	Perälä, 2008	Finland. South-west	general population	CIDI	both	Schizophrenia	10	Lifetime	8028	51	6.35	4.61	8.09
63	Perälä, 2008	Finland. West	general population	CIDI	both	Schizophrenia	10	Lifetime	8028	63	7.85	5.92	9.78
64	Phanthunane, 2010	Thailand	general population	others	both	SRD	12	Lifetime	11700	103	8.80	7.11	10.50
65	Phillips, 2004	China	general population	clinical	ICD	Schizophrenia	11	Point	19223	90	4.68	3.72	5.65
66	Phillips, 2009	China	general population	SCID	DSM	Schizophrenia	16	Point	63004	492	7.81	7.12	8.50
67	Pringle, 1995	Ireland	attended	clinical	DSM	Schizophrenia	11	12-month	37272	83	2.22	1.77	2.76
68	Ran, 2003	China	general population	clinical	ICD	Schizophrenia	14	Lifetime	89512	367	4.10	3.68	4.52
69	Roca, 1999	Spain	general population	SCAN	ICD	SRD	9	Point	697	3	5.00	0.89	12.53
70	Ruggeri, 2000	Italy	attended	clinical	ICD	NAP	13	12-month	62240	212	3.41	2.95	3.86
71	Schrier, 2001	Netherlands	attended	clinical	DSM	Schizophrenia	9	Point	337362	713	2.11	1.96	2.27
72	Scully, 2004	Ireland	attended	SCID	DSM	Schizophrenia	15	Lifetime	29542	115	3.59	3.21	4.67
73	Shivashankar, 2013	Scotland	general population	clinical	ICD	Schizophrenia	8	Point	205	1	4.88	-4.66	14.42
74	Singleton, 2003	UK	general population	SCAN	DSM	Probable	13	12-month	8886	44	4.95	3.49	6.41
75	Suvisaari, 2009	Finland	general population	CIDI	DSM	Schizophrenia	11	Lifetime	8028	66	8.22	0.74	2.50
76	Thornicroft, 1998	UK	attended	SCAN	ICD	NAP	13	12-month	80285	618	7.70	7.09	8.30
77	Tizon, 2007	Spain	attended	clinical	DSM	SRD	11	12-month	21236	97	4.57	3.66	5.47
78	Tizón, 2009	Spain	attended	clinical	DSM	SRD	9	Lifetime	103615	477	4.60	4.19	5.02
79	Vanasse, 2012	Canada	attended	clinical	ICD	Schizophrenia	9	Lifetime	5996925	35585	5.93	5.87	6.00
79	Vanasse, 2012	Canada	attended	clinical	ICD	Schizophrenia	9	12-month	5996925	7988	1.33	1.30	1.36
80	Vicente, 2004	Chile	general population	CIDI	DSM	NAP	13	12-month	2978	15	5.04	2.49	7.58
80	Vicente, 2004	Chile	general population	CIDI	DSM	NAP	13	Point	2978	15	5.04	2.49	7.58
81	Vicente, 2006	Chile	general population	CIDI	DSM	NAP	13	12-month	2978	21	7.05	4.05	10.06
81	Vicente, 2006	Chile	general population	CIDI	DSM	NAP	13	Lifetime	2978	54	18.13	13.34	22.93
82	Villaverde, 1993	Spain	general population	others	DSM	Schizophrenia	13	Point	660	4	6.06	0.14	11.98
83	Waldo, 1999	Micronesia	attended	SCID	DSM	Schizophrenia	10	Point	3235	22	6.80	3.97	9.63
84	Widerlöv, 1997	Sweden	attended	clinical	DSM	Schizophrenia	11	12-month	64886	273	4.21	3.71	4.71
85	Wu, 2006	USA	attended	clinical	ICD	Schizophrenia	7	12-month	6800000	34680	5.10	5.05	5.15
86	Xiang, 2008	China	general population	CIDI	ICD	Schizophrenia	15	Lifetime	5926	29	4.89	3.12	6.67
87	Yang, 2014	China	general population	CIDI	ICD	Schizophrenia	13	Point	1984	5	2.52	0.31	4.73
88	Youssef, 1999	Ireland	attended	clinical	DSM	Schizophrenia	11	12-month	21520	72	3.39	2.62	4.17

Attended: population attended in mental and/or social services.

SCID: Structure Clinical Interview for DSM-IV, CIDI: Composite International Diagnostic Interview, SCAN: Schedules for Clinical Assessment in Neuropsychiatry.

ICD: International Classification of Diseases, DSM: Diagnostic and Statistic Manual of Mental Disorders. Both: ICD and DSM.

NAP: Non-affective psychosis. SRD: Schizophrenia and related disorders. Probable: Probable psychotic disorders

LCI = Lower bound of 95% Confidence Interval; UCI: Upper bound of 95% Confidence Interval.

^1^Study quality according to criteria outlined in methodology. Min = 0, Max = 16.

^2^ Prevalence rate per 1.000.

The articles analyzed used different population settings to detect cases to be included in the studies; the majority of them (60.27%) were conducted in the general population and the rest in patients attended in health and/or social services. The method of confirming the diagnosis in the cases included in the studies in these articles was distributed as follows: 35.62% used the clinical diagnoses based on the criterion or clinical judgment of the referring physician, followed by 31.51% that used the CIDI, 10.96% used the SCID for DSM-IV, 8.22% used the SCAN and 13.7% included a variety of instruments such as DIGS, DIP, DIS, OPCRIT, or SADS. With respect to the classification of diseases on which the diagnoses were based, it is of note that 50.68% of the studies used the DSM in its versions III, III-R, IV and IV-TR, followed by 41.10% the ICD in its 7^th^, 8^th^, 9^th^ and 10^th^ versions. On several occasions both were used. The categories concerning the type of diagnosis in the studies were the following: 53.42% included only schizophrenia, 23.29% included patients with non-affective psychosis, 16.44% included patients diagnosed with schizophrenia and related disorders, and 6.85% included patients with a diagnosis of probable psychotic disorder. Quality of the studies. The mean quality score obtained through application of the Saha et al. criteria for all included articles was 11.75 points (of a possible total of 16) with all studies obtaining scores greater than 7 points.

### 3.2 Prevalence of psychotic disorders by subgroup

These 73 articles provided 101 prevalence rates based on an estimated total of 134,763 potentially overlapping cases. All data are provided in Table 1.

Tables 2, 3, 4 and 5 show the moments and quantiles for combined prevalence estimates for persons and by factors analyzed, showing the median prevalence for the 101 studies and by the four types of prevalence (global, point, 12-month and lifetime), for both sexes.

**Table 2:** Quantiles and moments of global prevalence per 1,000 persons.

Methodological variables	Number of studies	Quantiles					Mean	Standard deviation	IQR
		10%	25%	Median	75%	90%			
	101	2.11	3.24	4.60	7.2	11.03	6.18	5.10	3.95
**Case-finding method**									
General population	65	2.24	3.87	5.00	8.22	17.06	7.24	5.68	4.35
Attended in any health service	36	2.11	2.84	3.64	5.00	5.88	4.25	3.36	3.68
**Method of confirming diagnosis**									
SCID	10	3.56	4.86	7.50	9.07	16.30	9.02	6.38	4.21
CIDI	39	2.05	3.08	5.00	8.02	16.30	6.62	5.11	4.94
SCAN	6	3.99	4.26	4.98	5.08	6400	5.12	1.36	0.82
Clinical diagnosis	31	2.11	3.16	4.17	4.93	5.82	4.56	3.30	1.76
Others	15	2.30	2.94	3.75	7.71	15.46	6.89	7.20	4.77
**International classification of diseases**									
ICD	38	2.70	3.41	4.25	5.73	10.37	5.95	5.42	2.31
DSM	52	1.29	3.10	4.20	7.00	21.00	6.10	5.03	4.23
Both	11	3.70	4.04	6.35	9.01	10.71	7.25	4.60	5.00
**Diagnosis category**									
Schizophrenia only	49	2.05	2.78	4.21	6.35	8.82	5.30	4.00	3.57
Schizophrenia and related disorders	15	2.03	2.99	4.60	5.59	10.12	5.69	4.68	2.60
Non-affective psychosis	30	3.07	3.51	5.02	8.07	18.23	7.38	5.86	4.56
Probable schizophrenia	7	3.74	4.35	5.10	6.30	15.29	8.16	8.40	1.95

IQR: Interquartile range. SCID: Structure Clinical Interview for DSM-IV, CIDI: Composite International Diagnostic Interview, SCAN: Schedules for Clinical Assessment in Neuropsychiatry.

ICD: International Classification of Diseases, DSM: Diagnostic and Statistic Manual of Mental Disorders

**Table 3:** Quantiles and moments of point prevalence per 1,000 persons.

Methodological variables	Number of studies	Quantiles					Mean	Standard deviation	IQR
		10%	25%	Median	75%	90%			
	30	1.97	2.51	3.89	5.10	6.80	4.17	2.30	2.60
**Case-finding method**									
General population	18	1.76	2.65	4.44	5.77	7.12	4.54	2.64	3.12
Attended in any health service	12	2.11	2.11	3.11	5.10	5.33	3.61	1.59	2.89
**Method of confirming diagnosis**									
SCID	3	4.73	5.50	6.80	7.30	7.60	6.27	1.86	1.80
CIDI	9	1.67	2.50	3.04	5.04	6.18	3.60	2.06	2.54
SCAN	2	5.01	5.02	5.05	5.08	5.09	5.05	0.07	0.05
Clinical diagnosis	9	2.10	3.54	4.70	5.38	11.70	4.57	3.03	2.98
Others	7	1.85	2.51	3.10	3.60	4.67	3.24	1.49	1.09
**International classification of diseases**									
ICD	14	2.32	2.84	3.60	4.97	5.74	3.84	1.683	2.12
DSM	15	1.30	3.11	5.00	6.65	11.70	4.84	2.84	3.59
Both	1	4.04	4.04	4.04	4.04	4.04	4.04	-	0.00
**Diagnosis category**									
Schizophrenia only	15	2.03	2.38	3.46	5.45	6.50	3.93	2.07	3.07
Schizophrenia and related disorders	6	1.61	2.01	2.63	4.53	5.18	3.14	1.69	2.52
Non-affective psychosis	6	2.80	3.34	4.54	6.38	9.29	5.54	3.40	3.05
Probable schizophrenia	3	4.02	4.42	5.10	5.10	5.10	4.65	0.78	0.68

IQR: Interquartile range. SCID: Structure Clinical Interview for DSM-IV, CIDI: Composite International Diagnostic Interview, SCAN: Schedules for Clinical Assessment in Neuropsychiatry.

ICD: International Classification of Diseases, DSM: Diagnostic and Statistic Manual of Mental Disorders

**Table 4:** Quantiles and moments of 12-month prevalence per 1,000 persons.

Methodological variables	Number of studies	Quantiles					Mean	Standard deviation	IQR
		10%	25%	Median	75%	90%			
	37	2.03	3.20	4.03	4.97	7.79	4.69	3.24	1.77
**Case-finding method**									
General population	19	2.05	3.43	4.40	6.04	8.76	5.57	4.15	2.61
Attended in any health service	18	1.95	3.25	3.70	4.20	5.01	3.75	1.45	0.96
**Method of confirming diagnosis**									
SCID	-	-	-	-	-	-	-	-	-
CIDI	13	2.00	2.77	4.94	7.05	10.47	5.92	4.95	4.27
SCAN	4	3.98	4.00	4.49	5.63	6.87	5.16	1.75	1.63
Clinical diagnosis	17	1.86	3.00	3.70	4.21	4.73	3.57	1.12	1.01
Others	3	3.51	3.00	3.75	5.84	7.09	5.04	2.50	2.24
**International classification of diseases**									
ICD	16	3.02	3.44	3496	5.00	7.95	5.23	4.22	1.57
DSM	18	1.97	2.36	4.21	5.00	7.31	4.32	2.45	2.63
Both	3	3.77	3.87	4.04	4.10	4.14	3.97	0.24	0.23
**Diagnosis category**									
Schizophrenia only	17	1.72	2.22	3.70	4.21	6.23	4.51	4.238	1.99
Schizophrenia and related disorders	4	3.19	3.69	4.27	4.67	4.85	4.09	0.91	0.98
Non-affective psychosis	14	2.88	3.42	4.49	6.55	8.05	5.12	2.52	3.13
Probable schizophrenia	2	3.87	4.05	4.35	4.65	4.83	4.35	0.85	0.60

IQR: Interquartile range. SCID: Structure Clinical Interview for DSM-IV, CIDI: Composite International Diagnostic Interview, SCAN: Schedules for Clinical Assessment in Neuropsychiatry.

ICD: International Classification of Diseases, DSM: Diagnostic and Statistic Manual of Mental Disorders

**Table 5:** Quantiles and moments of lifetime prevalence per 1,000 persons.

Methodological variables	Number of studies	Quantiles					Mean	Standard deviation	IQR
		10%	25%	Median	75%	90%			
	34	3.62	4.63	7.49	10.92	19.67	9.57	6.69	6.29
**Case-finding method**									
General population	28	3.82	4.84	8.03	12.10	19.71	10.11	6.73	7.26
Attended in any health service	6	2.00	3.84	5.21	5.91	12.92	7.04	6.44	2.06
**Method of confirming diagnosis**									
SCID	7	3.45	5.39	8.72	12.31	18.87	10.20	7.33	6.92
CIDI	17	3.80	4.69	7.48	10.71	18.25	8.75	5.60	6.02
SCAN	-	-	-	-	-	-	-	-	-
Clinical diagnosis	5	4.30	4.60	5.82	5.93	13.85	7.92	6.31	1.33
Others	6	4.44	7.49	8.80	19.90	24.16	13.12	10.05	12.41
**International Classification of diseases**									
ICD	8	4.52	4.84	6.71	16.36	21.49	11.08	8.49	11.52
DSM	19	3.52	3.91	7.19	10.09	20–13	9.09	6.69	6.17
Both	7	4.77	7.10	8.80	9.97	13.80	9.11	4.90	2.86
Others	-	-	-	-	-	-	-	-	-
**Diagnosis category**									
Schizophrenia only	17	3.11	4.10	6.35	8.72	12.60	7.30	4.41	4.62
Schizophrenia and related disorders	5	5.09	5.82	8.80	10.99	16.33	10.02	6.06	5.17
Non-affective psychosis	10	3.66	4.66	8.19	18.88	21.36	11.66	8.03	14.21
Probable schizophrenia	2	9.44	12.37	17.24	22.12	25.04	17.24	13.79	9.75

IQR: Interquartile range. SCID: Structure Clinical Interview for DSM-IV, CIDI: Composite International Diagnostic Interview, SCAN: Schedules for Clinical Assessment in Neuropsychiatry.

ICD: International Classification of Diseases, DSM: Diagnostic and Statistic Manual of Mental Disorders

From the 101 prevalence estimates, the median global prevalence for persons across these studies was 4.6 per 1000, and the 10% and 90% quantiles ranged from 2.11 to 11.03 per 1000, respectively (a 5.2-fold difference) (Table 2). A total of 25 articles [17,19,20,22,23,29,33,35,38,40,41,45,53,56,59,62,65,66,69,71,73,80,82,83,87] estimated 30 rates of point prevalence of psychotic disorders and the median across these studies was 3.89 per 1000, and the 10% and 90% quantiles ranged from 1.98 to 6.8 per 1000, respectively (a 3.43-fold difference) (Table 3). Thirty-six of the articles [19,20,23,25,26,27,30,34,35,36,37,42,43,44,48,49,50,51,52,54,55,57,58,59,61,67,70,74,76,77,79,80,81,84,85,88] considered 37 estimates rates of the 12-month prevalence. The median 12-month prevalence across these studies was 4.03 per 1000 persons, and the 10% and 90% quantiles ranged from 2.03 to 7.79 per 1000 (a 3.84-fold difference) (Table 4). Lifetime prevalence was considered in 28 of the articles [8,18,19,21,23,24,26,28,31,32,35,37,39,46,47,49,51,52,60,63,64,68,72,75,78,79,81,86], which reported 34 estimated rates. The median lifetime prevalence for persons across these studies was 7.49 per 1000, and the 10% and 90% quantiles ranged from 3.62 to 19.67 per 1000 (a 5.43-fold difference) (Table 5).

**Fig 2:**
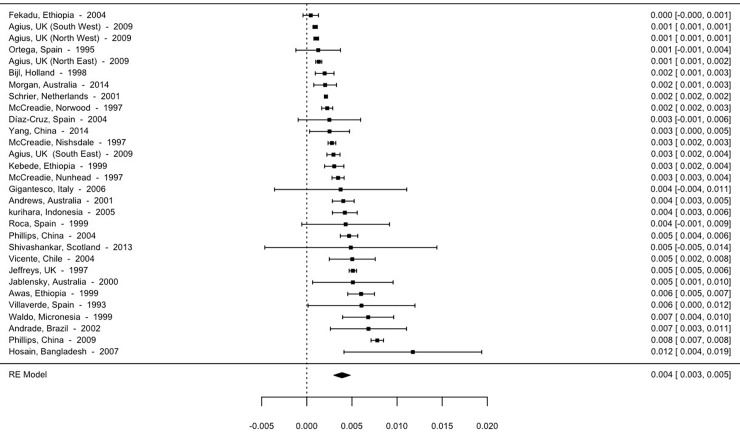
Forest plot point prevalence of psychotic disorders.

**Fig 3:**
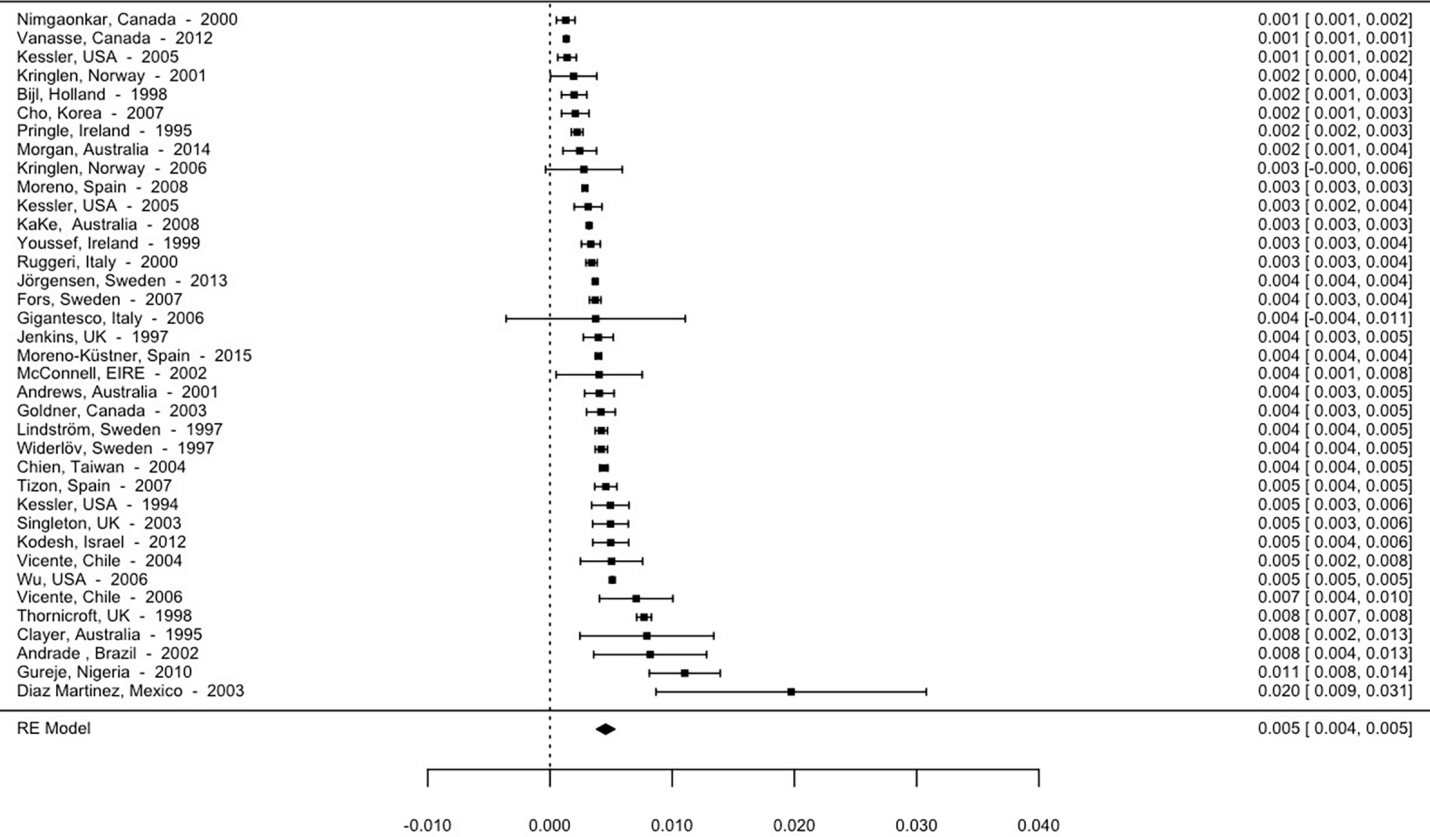
Forest plot 12-month prevalence of psychotic disorders.

**Fig 4:**
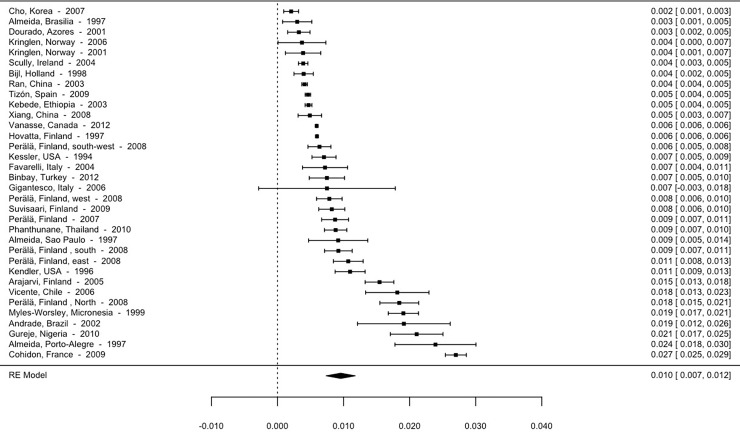
Forest plot lifetime prevalence of psychotic disorders.

Although the high heterogeneity between the studies and the distribution of the prevalence rates themselves detract from the calculation of the mean effect, making use of the median advisable, we present the means below. Thus, the overall mean prevalence is 5.93 per 1000 (95% CI; 5.63–6.24). The mean prevalence for studies using point prevalence is 3.9 per 1000 (95% CI, 3.28–4.52). The 12-month prevalence is 4.56 per 1000 (95% CI, 4.09–5.03), and lifetime prevalence is 9.57 per 1000 (95% CI, 9.01–10.13). The forest plot for point, 12-month and lifetime prevalence are shown in Figs 2–4.

This review identified that the median estimate for point prevalence was lower when the case-finding setting was health and/or social services (3.11 per 1000) and 1.4-fold higher when the study was performed in the general population (4.44 per 1000). The median 12-month prevalence estimate from general population studies (4.40 per 1000) was 1.2-fold higher than from attended population studies (3.70 per 1000). Similarly, the lifetime prevalence rates were 1.5-fold higher in general population studies (8.03 per 1000) than in attended population studies (5.21 per 1000).

Prevalence estimates were obtained from different methods of confirming diagnoses. The point prevalence median range was from a maximum of 6.80 per 1000 when the SCID was used, to a minimum of 3.04 per 1000 when CIDI were used (2.24-fold difference). Conversely, in the 12-month prevalence studies, the rate was higher when CIDI were used (4.94 per 1000), and the lowest median prevalence estimate (3.70 per 1000) was with the clinical diagnosis. Likewise, the lifetime prevalence studies group showed the highest median prevalence using other instruments to confirm the diagnosis of psychosis (8.8 per 1000) and the lowest when using the clinical criteria (5.82 per 1000).

In the point prevalence studies, the highest median estimate of 4.21 per 1000 was found when the DSM was used, while this figure was 1.2-fold lower when the ICD was used with a median of 3.60 per 1000. In the studies that calculated 12-month prevalence rates, the ICD diagnostic classification system presented slightly a lower median estimate of 3.96 per 1000, while with the DSM this figure was 4.20 per 1000. In the lifetime prevalence studies, the DSM classification was the most often used and offered a higher median estimate of 7.19 per 1000 compared to that of the ICD which was 6.71 per 1000.

Each diagnosis category displayed differences in point prevalence rate estimates. When studies included schizophrenia and related disorders, the median estimated rate was lower (2.62 per 1000) than when schizophrenia only was analyzed (3.46 per 1000 inhabitants), non-affective psychoses was higher (4.53 per 1000) and the highest was probable psychosis (5.10 per 1000). In 12-month prevalence estimates, the highest median estimated rate was similar in non-affective psychosis and probable schizophrenia (4.5 and 4.35 per 1000), lower for schizophrenia and related disorders (4.26 per 1000) and the lowest for schizophrenia only (3.7 per 1000). The highest median estimate of lifetime prevalence was probable psychosis (17.24 per 1000), followed by schizophrenia and related disorders (8.8 per 1000), non-affective psychosis (8.18 per 1000) and schizophrenia only (6.35 per 1000).

When the point prevalence estimates for persons were divided into quality score quantiles, the median quality score and corresponding IQR was 12.00 (IQR = 3.0) across the point and 12-month prevalence studies and 13.00 (IQR = 5) for the lifetime prevalence studies.

### 3.3 Association between methodological issues and the prevalence of psychotic disorders

The pooled prevalence rate of psychotic disorders revealed that heterogeneity in the studies was high (*I*^2^ = 99.8%). Therefore, it was considered important to examine and try to explain the possible methodological sources of heterogeneity that could be present in the studies included in the review. Accordingly, in this analysis we included the following variables: prevalence type, case-finding setting, method of confirming diagnosis, international classification of diseases, diagnosis categories and study quality.

The random-effects meta-regression analysis showed a significant effect for the prevalence type, with higher lifetime prevalence rates than the 12-month prevalence rates (p<0.0001). Also, a higher study quality was associated with a lower estimated prevalence of psychotic disorders (p = 0.0002). On the other hand, the diagnosis category probable psychotic disorder presented a higher prevalence rate than schizophrenia (p = 0.022) as did the non-affective psychosis group compared to the schizophrenia only group (p = 0.0091) (Table 6). Finally, studies conducted in the general population presented higher prevalence rates than those carried out in populations attended in health and/or social services (p = 0.0059). This analysis revealed that neither the disease classification used, nor the diagnosis instrument applied had a significant effect on the prevalence rate (results not shown). We have found that the proportion of the variance that is explained in the final adjusted model is 40.6%.

**Table 6:** Results of the meta-regression. Random-effects model sizes of the prevalence of psychotic disorders.

Variables	Coefficients	LCI	UCI	p
Intercept	0.0108	0.0064	0.0152	< .0001
**Prevalence type**				
12-month (reference)				
Point	-0.0006	-0.0025	0.0014	0.5537
Lifetime	0.0050	0.0031	0.0070	< .0001
**Case-finding setting**				
Population attended in any health service (reference)				
General population	0.0028	0.0008	0.0047	0.0059
**Diagnosis categories**				
Schizophrenia(reference)				
Schizophrenia and related disorders	-0.0004	-0.0026	0.0019	0.7586
Non-affective psychosis	0.0026	0.0006	0.0045	0.0091
Probable psychotic disorders	0.0042	0.0006	0.0078	0.0222
**Quality of the studies**	-0.0008	-0.0012	-0.0004	0.0002

I^2^ = 99.8%; R^2^ = 40.6

LCI = Lower bound of 95% Confidence Interval; UCI: Upper bound of 95% Confidence Interval

I^2^ = variability due to true heterogeneity

R^2^ = Coefficient of Determination

### 3.4 Publication bias

The results of the Egger’s test showed no statistical differences (z = -0.6769,p = 0.4985) and the visual inspection of the funnel plot revealed the absence of publication bias.

## Discussion

To our knowledge, this is the first systematic review including a meta-analysis summarizing the study methodology used in estimating the prevalence of psychosis globally. In addition to updating the previous systematic reviews up to 2015, we have provided a summary of the prevalence estimates by methodology undertaken and presented results of a meta-regression analysis showing the methodological aspects of the studies that influenced the variability estimates. We have also presented the proportion of the variance that is explained in the final adjusted model. Previous systematic reviews focused mainly on the descriptive findings concerning several aspects of the prevalence of schizophrenia [5,10,11]. However, in our review we also applied a meta-regression analysis.

Our review identified 73 studies from the literature on the prevalence of schizophrenia and psychotic disorders in English and Spanish published over a 25-year period (1990–2015). We used the year 1990 as a cut-off to limit our search, following Simeone et al. [11] criteria which verified that no major studies were excluded prior this date. Additionally, as they also state, since the treatment, diagnostic criteria and guidelines of diseases changes over the years, it would be not be useful to include previous years. We focused on describing the methodology adopted in each study and its association with the variability of estimated prevalence rates of psychosis and related disorders. This approach confirmed differences in rates worldwide by prevalence type, case-finding setting, diagnosis category and study quality. Conversely, the method of confirming diagnosis and the international classification of diseases were not associated with the variability of the prevalence estimates.

### 4.1 Principal findings

The descriptive results of the pooled median global prevalence of psychotic disorders was 4.6 per 1000 persons, while the median point and 12-month prevalence was 3.89 and 4.03 per 1000 persons respectively and the median lifetime prevalence was 7.49 per 1000 persons.

Meta-regression analysis showed the methodological aspects of the studies that influenced the variability of the prevalence estimates. Concerning prevalence type, lifetime prevalence was higher than 12-month prevalence (p<0.0001). Studies developed in the general population presented higher prevalence estimates that those developed in a population attended in health and/or social services (p = 0.0059), the diagnosis categories of probable psychotic diagnoses (p = 0.022) and non-affective psychosis (p = 0.0091) both present higher estimate rates than the diagnosis of schizophrenia only. Finally, a higher study quality is associated with lower estimates of prevalence of psychotic disorders (p = 0.0002). Additionally, the publication bias analyses revealed the absence of bias, thus corroborating the robustness of our results.

### 4.2 Meaning of findings

The median point prevalence of psychotic disorders was 3.89 per 1000 persons. This estimate is consistent with the earlier systematic review of 188 studies by Saha et al. [10] who reported median point prevalence estimates of 4.6 per 1000 persons. However, our 12-month prevalence figures (4.03 per 1000 persons) were higher than the 3.3 per 1000 persons found in previous systematic reviews [10,11]. We also found higher figures for median lifetime prevalence (7.49 per 1000) than the 4.0 and 4.8 found by Saha et al. and Simeone et al., respectively [10,11]. Based on the central 80% of the estimates (10% to 90% quantiles), the present review found that the different types of prevalence estimates had from 4.5-fold (point and 12-month) to 7.9-fold (lifetime) variation. Our figures on variation are again higher that the figures found by Saha et al. [10], which were 3.4-fold (point) to 4.6-fold (12-month) variation. In the systematic review by Goldner et al. [5], based on the 100% estimates (not the central 80%), a 5-fold variation for 12-month and lifetime prevalence for psychotic disorders was observed.

The following step is to determine how much of this variation is due to measurement error (methodological aspects). Thus, as methodology-dependent factors likely lead to variations in published estimates, the present review used a meta-regression analysis to determine the estimated effect size attributable to these factors.

Our findings suggest that four methodological factors (prevalence type, case-finding setting, diagnostic categories and study quality) contribute 40.6% of the variance in prevalence estimates. As with other systematic reviews of prevalence estimates, a significant heterogeneity (Q with p < .0001) explored by *I*^2^ was found in the measurement of prevalence rates that were incompletely explained by meta-regression. These results are described below.

Our results show that the mean lifetime prevalence estimates for psychotic disorders are significantly higher than the mean 12-month prevalence estimates (p<0.0001). It is reasonable to assume that lifetime prevalence should be higher than 12-month prevalence, as the study period in the first case includes all one’s life up to the time of assessment, which implies that most cases can be found. Surprisingly, however, the data from the review by Saha et al. [10] does not support this assumption. We found similar estimates in both the point and 12-month prevalence types, which suggest that in a chronic disease such as schizophrenia, assessing these prevalence types does not affect estimates.

The choice of study setting (e.g. general population versus attended in health and/or services population studies) does play an important role in identifying those persons with a mental health disorder such as psychosis. Studies conducted in the general population present higher prevalence rates (p = 0.0059) than those carried out in populations attended in health/social services. This result is in agreement with the study by Saha et al. [10] that concluded that the use of an exhaustive method to identify cases such as door-to-door surveys or interviews based on several community information sources could identify more cases than those using few sources of information. Simeone et al. [11] also found different results according to the scope of the study, in the sense that, in studies that included only hospitalized patients, the prevalence figures were up to 60% lower than those that included patients treated both at the hospital and outpatient levels.

One explanation for higher prevalence rates of schizophrenia in general population studies is that previous studies have shown lay-administered interviews overestimate prevalence (e.g. NCS-R) [48]. And it is now widely appreciated that many otherwise healthy individuals in the community report experiencing isolated psychotic experiences (e.g. lifetime prevalence = 5.8%) [89].

Cross-sectional population-based study designs for low-prevalence disorders, such as psychosis, present some drawbacks [4,9]. In this type of study it will be necessary to identify and evaluate many healthy people to reach a representative sample for reliable results [4]. Also, interviews of community members by mental health professionals for symptoms indicative of schizophrenia are time-consuming and expensive to conduct. Moreover, people with schizophrenia are probably less likely to be available for interview, or to agree to an interview if contacted. We agree that the most accurate way to assess schizophrenia and related disorders prevalence would involve full clinician interviews with the entirety of a population. However, since this is not practical in studies with large samples, a more cost-effective strategy could be to identify potential cases in a representative sample of the study population and subsequently carry out clinical interviews to confirm the diagnosis of those subjects to include them in the study [11]. One solution is to perform studies similar to the survey by Perälä et al. in Finland [8] in which they concluded that the use of many sources of information are crucial to attain precise figures of the lifetime prevalence of psychotic disorders, reporting a lifetime prevalence greater than 3%. Additionally, Moreno-Küstner et al., [58] developed a primary study of persons with schizophrenia and related disorders, based on a range of large health services databases including inpatient and outpatient records, emergency services and general practitioner surveys to attain accurate figures.

Concerning diagnosis categories, as was expected, meta-regression confirmed that the broader category of probable psychosis (p = 0.022) and non-affective psychosis (p = 0.0091) had a higher prevalence than the diagnosis of schizophrenia only. This result is in agreement with the study by Simeone et al. [11] which showed that there could be a prevalence increase of more than 70% when using a more comprehensive definition of the spectrum of "schizophrenia and associated disorders" compared to a narrower definition. Heterogeneity remained high in the probable psychosis category, but we chose a pragmatic approach to the diagnosis of psychotic disorders, given changing classification over time and between studies. This approach has construct validity with overall pooled prevalence rates [90].

We found that the quality of the study had an impact on prevalence estimates. Thus, improved study quality was associated with lower prevalence estimates (p = < .0001). This result is similar to that found in the study by Simeone et al. [11] in which prevalence rates were higher in studies with low levels of quality, which probably indicates that the actual prevalence rate of schizophrenia is lower than that offered in those studies with low quality. However, although we used the method developed by Saha et al. [10] to assess certain items concerning the quality of the original data, our results conflict with those of Saha et al. [10] who found that studies with higher overall quality scores tended to identify more cases and thus generate higher prevalence estimates than lower quality studies. Future studies could explore the impact of quality on the variation in prevalence estimates.

No differences were found between the methods of confirming diagnoses. Our results show that the method chosen to confirm the diagnosis was not associated with the variability of the prevalence estimates. This is very important, as it implies that any of the instruments mentioned below, patient interviews or clinical judgment may be used to confirm the diagnosis as this factor does not play an important role in the prevalence estimates.

In order to count the disorders of interest, it is essential to have reliable diagnostic criteria to define cases. Several validated instruments are available for the assessment of a range of mental disorders. These include the CIDI [91], SCID [92], SCAN [93], and the Mini-International Neuropsychiatric Interview [94]. A significant advantage of a number of these instruments is that they are designed to be administered by trained lay-interviewers allowing for rapid surveying of large samples. The instruments used may vary from brief symptom checklists used in the clinical diagnoses made by the professional, to fully standardized surveys, and finally, the gold standard of the clinical interview.

This review sought epidemiological data for mental disorders defined according to DSM or ICD diagnostic criteria. We found no differences associated with any of these international classifications in the prevalence estimates. We have analysed the moderating effect of different versions of the classification of diseases (ICD; 8th, 9th and 10th and DSM; III, IV). We have included them in the bi-variate analyses but found no association with the dependent variable. Simeone et al. [11] found only minor differences in prevalence estimates of schizophrenia calculated using different diagnostic criteria such as ICD-9 vs. ICD-10. This result has an important implication for research on psychotic disorders, which is that using ICD or DSM classification is not associated with prevalence estimates. Further research using DSM-5 should be carried out to confirm this result.

### 4.3 Limitations

Although we used a comprehensive search and selection strategy of the published literature based on a validated and reliable methodology to minimize missed studies, we had no help in designing or conducting the search from informaticians or documentalists, so a number of data gaps became evident in the course of conducting this review. Another limitation is the exclusion of non-English and non-Spanish literature. However, cross-checking English abstracts of excluded studies showed that few studies (including no major studies) were missed given the language restriction which appears to reflect that studies today are commonly published in English. Furthermore, the age range of the study samples included in this review varied greatly, which limited our ability to use age range as a variable for sub-analyses. The scope of this review was restricted only to general populations or out-patients attended in health and/or social services, rather than including focused populations such as institutionalized or incarcerated patients, homeless persons, and migrants. Special populations such as these do have a higher reported prevalence of psychotic disorders so the prevalence rates could be higher than the rate reported here. But these rates should be described separately, so as not to overestimate the prevalence in the general population. Finally, since the methodologies of the individual studies present large differences, pooled effect sizes should be interpreted in line with corresponding *I*^2^ statistics [95].

### 4.4 Conclusions and practical implications

Despite the wide variation in the methodological components of the studies reviewed, these data indicate that approximately one in 150 individuals will be diagnosed with psychosis disorders at some point during their lifetime. Prevalence estimates across studies varied when looking at different periods of assessment, study design setting, diagnosis categories and quality scores. Thus, a well-designed epidemiological study with homogeneous methodology will help to improve our understanding of the global prevalence of this disease.

One of the principal clinical implications is that when a systematic review of these types of data is conducted rigorously and data analyzed appropriately, these reviews can be of great benefit to healthcare professionals and policy makers. This evidence suggests that a focus on a sub-group of studies that meet a number of criteria (e.g. study quality, prevalence type, setting design) may provide a better reflection of the true prevalence of psychosis.

This updated review provides vital evidence on the epidemiology of psychosis in general and attended populations, which is important information for healthcare planning. The present review provides an overall comprehensive comparison of methodologies used in psychotic disorders prevalence studies, which could generate insightful information for future epidemiological studies in adopting the relevant methodological approach.

## Supporting information

S1 Appendix PRISMA checklist. Details of how this systematic review conformed to the PRISMA standards for systematic reviewing.
(DOC)

S2 Appendix Card for each article. (DOC)

S1 Table Table with definitions for the variables used to characterize the prevalence studies. (DOC)

S2 Table Reporting scale for assessing study quality. (DOC)
