# Supplementary material for: Prevalence of psychotic disorders and its association with methodological issues. A systematic review and meta-analyses
Source: PLoS One. 2018 Apr 12;13(4):e0195687. doi: 10.1371/journal.pone.0195687 (PMC5896987; doi:10.1371/journal.pone.0195687)
Supplement: S2 Appendix — (DOC) [file pone.0195687.s002.doc]

**S2 Appendix. CARD FOR EACH ARTICLE**

**REFERENCE NUMBER:** 17

**AUTHORS:** Agius M and Ward C.

**YEAR OF PUBLICATION:** 2009.

**TITLE:** The epidemiology of psychoses in Luton.

**JOURNAL:** *Psychiatria Danubina, 21*(4), 508-513.

**===============================================================================================**

**STUDY COUNTRY:** United Kingdom. **STUDY YEAR:** 2001.

**CASE FINDING SETTING:** attended population in Mental Health Services (South East, North West, North East and South West).

**SAMPLING:** nonrandom.

**If sampling, indicate Clusters: CL1-CL2-P/CL:**

**NUMBER OF STAGES:** 1.

**SCREENING INSTRUMENT:** It is not used as it is in a phase.

**RESPONSE % PHASE 1: RESPONSE % PHASE 2:**

**DIAGNOSTIC INSTRUMENT:** clinical.

**CLASSIFICATION OF DISEASES:** DSM- IV.

**DIAGNOSTIC CATEGORIES:**Schizophrenia and related disorders

**STUDY AREA:** Luton. **POPULATION SIZE (denominator):** 184,371 (21,350 SE+ 43,829 NE+ 57,414 NW+ 61,778 SW).

**AGE RANGE: LOWER:** 16. **UPPER:** 74.

**STUDY QUALITY:** 7.

**===============================================================================================**

**NUMBER OF ESTIMATES:** 42 + 137 + 122 + 331 (between 4 teams Mental Health).

**PREVALENCE RATE (indicated period: point, 12-month, lifetime)**

**PREVALENCE RATE (Point):** 1.97 / 3.13 / 2.12 / 5.36 per 1,000 inhabitants (for each of the 4 Mental Health teams).

**STANDARD ERROR: CI (confidence interval): LOW: UPPER:**

**MEN: STANDARD ERROR: CI: LOW: UPPER:**

**WOMEN: STANDARD ERROR: CI: LOW: UPPER:**

**===============================================================================================**

**OBSERVATIONS:**

**REFERENCE NUMBER:** 18

**AUTHORS:** Almeida-Filho N, Mari Jair De J, Coutinho E; Franca Josimar F, Fernandes J, Andreoli SB, Busnello and Ellis D'A.

**YEAR OF PUBLICATION:** 1997.

**TITLE:** Brazilian multicentric study of psychiatric morbidity. Methodological features and prevalence estimates.

**JOURNAL:** *The British Journal of Psychiatry, 171,* 524-529.

**===============================================================================================**

**STUDY COUNTRY:** Brazil. **STUDY YEAR:** 1991.

**CASE FINDING SETTING:** census, general population.

**SAMPLING:** nonrandom.

**If sampling, indicate Clusters: CL1-CL2-P/CL:**

**NUMBER OF STAGES:** 2.

**SCREENING INSTRUMENT:** SCID (Structure Clinical Interview for DSM-IV).

**RESPONSE % PHASE 1: RESPONSE % PHASE 2:**

**DIAGNOSTIC INSTRUMENT:** SCID (Structure Clinical Interview for DSM-IV).

**CLASSIFICATION OF DISEASES:** DSM- III.

**DIAGNOSTIC CATEGORIES:** non-affective psychoses.

**STUDY AREA:** 3 regions (Brasilia, Porto Alegre and Sao Paulo).

**POPULATION SIZE (denominator):** Brasilia (2,345), Porto-Alegre (2,384) and Sao-Paulo (1,742).

**AGE RANGE: LOWER:** 15. **UPPER:** 90.

**STUDY QUALITY:** 15.

**===============================================================================================**

**NUMBER OF ESTIMATES:** 80 between the 3 regions (7 Brasilia + 57 Porto Alegre + 16 Sao Paulo).

**PREVALENCE RATE (indicated period: point, 12-month, lifetime)**

**PREVALENCE RATE (Lifetime):** 3.24 (Brasilia) / 24.00 (Porto Alegre) / 9.00 (Sao Paulo). Per 1,000 inhabitants.

**STANDARD ERROR:** 0.40. **CI: LOW: UPPER:**

**MEN: STANDARD ERROR: CI: LOW: UPPER:**

**WOMEN:** 5.00 (Brasilia) / 25.00 (Porto Alegre) / 1.20 (Sao Paulo) per 1,000 inhabitants.

**STANDARD ERROR: CI: LOW: UPPER:**

**===============================================================================================**

**OBSERVATIONS:**

**REFERENCE NUMBER:** 19

**AUTHORS:** Andrade L, Walters E, Gentil V and Laurenti R.

**YEAR OF PUBLICATION:** 2002.

**TITLE:** Prevalence of ICD-10 mental disorders in a catchment area in the city of Sao Paulo, Brazil.

**JOURNAL:** [*Social Psychiatry and Psychiatric Epidemiology,*](javascript:AL_get(this, 'jour', 'Soc Psychiatry Psychiatr Epidemiol.');) *37*(7), 316-25.

**===============================================================================================**

**STUDY COUNTRY:** Brazil. **STUDY YEAR:** 2000.

**CASE FINDING SETTING:** census, general population.

**SAMPLING:** nonrandom.

**If sampling, indicate Clusters: CL1-CL2-P/CL:**

**NUMBER OF STAGES:** 1.

**SCREENING INSTRUMENT:**

**RESPONSE % PHASE 1: RESPONSE % PHASE 2:**

**DIAGNOSTIC INSTRUMENT:** CIDI (Composite International Diagnostic Interview).

**CLASSIFICATION OF DISEASES:** ICD-10.

**DIAGNOSTIC CATEGORIES:** non-affective psychoses.

**STUDY AREA:** Sao Paulo. **POPULATION SIZE (denominator):** 1,464.

**AGE RANGE: LOWER:** 18. **UPPER:** 90.

**STUDY QUALITY:** 13.

**===============================================================================================**

**NUMBER OF ESTIMATES:** 50 (12 + 10 + 28).

**PREVALENCE RATE (indicated period: point, 12-month, lifetime)**

**PREVALENCE RATE (Point):** 6.83 per 1,000 inhabitants. **STANDARD ERROR:** 2.00  **CI: LOW: UPPER:**

**PREVALENCE RATE (12-month):** 8.20 per 1,000 inhabitants. **STANDARD ERROR:** 2.00 **CI: LOW**: **UPPER:**

**PREVALENCE RATE (Lifetime):** 19.13 per 1,000 inhabitants. **STANDARD ERROR:** 3.00 **CI: LOW**: **UPPER:**

**MEN:** point (5.00) / 12-month (8.00) / lifetime (17.00). **STANDARD ERROR: CI: LOW**: **UPPER:**

**WOMEN:** point (8.00) / 12-month (8.00) / lifetime (20.00). **STANDARD ERROR: CI: LOW:**   **UPPER:**

**===============================================================================================**

**OBSERVATIONS:**

**REFERENCE NUMBER:** 20

**AUTHORS:** Andrews G, Henderson S and Hall W.

**YEAR OF PUBLICATION:** 2001.

**TITLE:** Prevalence, comorbidity, disability and service utilisation.

**JOURNAL:** *The British Journal of Psychiatry, 178,* 145-153.

**===============================================================================================**

**STUDY COUNTRY:** Australia. **STUDY YEAR:** 1997.

**CASE FINDING SETTING:** census, general population.

**SAMPLING:** cluster.

**If sampling, indicate Clusters: CL1-CL2-P/CL:** CL1.

**NUMBER OF STAGES:** 1.

**SCREENING INSTRUMENT:**

**RESPONSE % PHASE 1: RESPONSE % PHASE 2:**

**DIAGNOSTIC INSTRUMENT:** CIDI (Composite International Diagnostic Interview).

**CLASSIFICATION OF DISEASES:** ICD-10 + DSM-IV.

**DIAGNOSTIC CATEGORIES:** non-affective psychoses.

**STUDY AREA:** Australia. **POPULATION SIZE (denominator):** 10,641.

**AGE RANGE: LOWER:** 18. **UPPER:** 90.

**STUDY QUALITY:** 15.

**===============================================================================================**

**NUMBER OF ESTIMATES:** 43.

**PREVALENCE RATE (indicated period: point, 12-month, lifetime)**

**PREVALENCE RATE (Point):** 4.04 per 1,000 inhabitants. **STANDARD ERROR:** 1.0  **CI: LOW: UPPER:**

**PREVALENCE RATE (12-month):** 4.04 per 1,000 inhabitants. **STANDARD ERROR:** 1.0  **CI: LOW: UPPER:**

**MEN: STANDARD ERROR: CI: LOW: UPPER:**

**WOMEN:**   **STANDARD ERROR: CI: LOW: UPPER:**

**===============================================================================================**

**OBSERVATIONS:**

**REFERENCE NUMBER:** 21

**AUTHORS:** Arajärvi R, Suvisaari J, Suokas J, Schreck M, Haukka J, Hintikka J, Lönnqvist J and Partonen T.

**YEAR OF PUBLICATION:** 2005.

**TITLE:** Prevalence and diagnosis of schizophrenia based on register, case record and interview data in an isolated Finnish birth cohort born 1940–1969.

**JOURNAL:** *Social Psychiatry and Psychiatric Epidemiology, 40*(10), 808-816.

**===============================================================================================**

**STUDY COUNTRY:** Finland **STUDY YEAR:** 1998.

**CASE FINDING SETTING:** attended population in Mental Health Services + Primary Care + Social Services.

**SAMPLING:** simple random.

**If sampling, indicate Clusters: CL1-CL2-P/CL:**

**NUMBER OF STAGES:** 1.

**SCREENING INSTRUMENT:**

**RESPONSE % PHASE 1: RESPONSE % PHASE 2:**

**DIAGNOSTIC INSTRUMENT:** SCID (Structure Clinical Interview for DSM-IV).

**CLASSIFICATION OF DISEASES:** ICD-8 + ICD-9 + ICD-10.

**DIAGNOSTIC CATEGORIES:** Schizophrenia.

**STUDY AREA:** Finland. **POPULATION SIZE (denominator):** 12,368.

**AGE RANGE: LOWER:** 40. **UPPER:** 69.

**STUDY QUALITY:** 14.

**===============================================================================================**

**NUMBER OF ESTIMATES:** 191.

**PREVALENCE RATE (indicated period: point, 12-month, lifetime)**

**PREVALENCE RATE (Lifetime):** 15.44 per 1,000 inhabitants. **STANDARD ERROR:**   **CI: LOW: UPPER:**

**MEN: STANDARD ERROR: CI: LOW: UPPER:**

**WOMEN:**   **STANDARD ERROR: CI: LOW: UPPER:**

**===============================================================================================**

**OBSERVATIONS:**

**REFERENCE NUMBER:** 22

**AUTHORS:** Awas M, Kebede D and Alem A.

**YEAR OF PUBLICATION:** 1999.

**TITLE:** Major mental disorders in Butajira, southern Ethiopia.

**JOURNAL:** *Acta Psychiatrica Scandinavica Supplementum, 397*, 56-64.

**===============================================================================================**

**STUDY COUNTRY:** Ethiopia.  **STUDY YEAR:** 1996.

**CASE FINDING SETTING:** census, general population.

**SAMPLING:** cluster.

**If sampling, indicate Clusters: CL1-CL2-P/CL:** CL2.

**NUMBER OF STAGES:** 2.

**SCREENING INSTRUMENT:**

**RESPONSE % PHASE 1:** 15%. **RESPONSE % PHASE 2:**

**DIAGNOSTIC INSTRUMENT:** CIDI (Composite International Diagnostic Interview).

**CLASSIFICATION OF DISEASES:** ICD-10.

**DIAGNOSTIC CATEGORIES:** Schizophrenia.

**STUDY AREA:** Ethiopia.  **POPULATION SIZE (denominator):** 10,468.

**AGE RANGE: LOWER:** 15. **UPPER:** 90.

**STUDY QUALITY:** 15.

**===============================================================================================**

**NUMBER OF ESTIMATES:** 63.

**PREVALENCE RATE (indicated period: point, 12-month, lifetime)**

**PREVALENCE RATE (Point):** 6.02 per 1,000 inhabitants. **STANDARD ERROR:**   **CI: LOW: UPPER:**

**MEN:** point (2.00) / lifetime (4.00) per 1,000 inhabitants.  **STANDARD ERROR: CI: LOW: UPPER:**

**WOMEN:** point (9.00) / life (11.00) per 1,000 inhabitants. **STANDARD ERROR: CI: LOW: UPPER:**

**===============================================================================================**

**OBSERVATIONS:**

**REFERENCE NUMBER:** 23

**AUTHORS:** Bijl R, Ravelli A and Zessen G.

**YEAR OF PUBLICATION:** 1998.

**TITLE:** Prevalence of psychiatric disorder in the general population: results of the Netherlands Mental Health Survey and Incidence Study (NEMESIS).

**JOURNAL:** *Social Psychiatry and Psychiatric Epidemiology, 33*(12), 587-595.

**===============================================================================================**

**STUDY COUNTRY:** Holland.  **STUDY YEAR:** 1996.

**CASE FINDING SETTING:** census, general population.

**SAMPLING:** cluster.

**If sampling, indicate Clusters: CL1-CL2-P/CL:** CL1.

**NUMBER OF STAGES:** 1.

**SCREENING INSTRUMENT:**

**RESPONSE % PHASE 1:** 69%. **RESPONSE % PHASE 2:**

**DIAGNOSTIC INSTRUMENT:** CIDI (Composite International Diagnostic Interview).

**CLASSIFICATION OF DISEASES:** DSM-III-R.

**DIAGNOSTIC CATEGORIES:** Schizophrenia.

**STUDY AREA:** Holland.  **POPULATION SIZE (denominator):** 7,076.

**AGE RANGE: LOWER:** 18. **UPPER:** 64.

**STUDY QUALITY:** 16.

**===============================================================================================**

**NUMBER OF ESTIMATES:** 14 (12-month) + 14 (Point) + 28 (Lifetime).

**PREVALENCE RATE (indicated period: point, 12-month, lifetime)**

**PREVALENCE RATE (Point):** 1.98 per 1,000 inhabitants. **STANDARD ERROR:** 0.05  **CI: LOW: UPPER:**

**PREVALENCE RATE (12-month):** 1.98 per 1,000 inhabitants. **STANDARD ERROR:** 1.00  **CI: LOW: UPPER:**

**PREVALENCE RATE (Lifetime):** 3.96 per 1,000 inhabitants. **STANDARD ERROR:** 1.00  **CI: LOW: UPPER:**

**MEN:** point (1.00) / 12-month (2.00) / life (4.00) per 1,000 inhabitants. **STANDARD ERROR: CI: LOW**: **UPPER:**

**WOMEN:** point (2.00) / 12-month (2.00) / life (3.00) per 1,000 inhabitants. **STANDARD ERROR: CI: LOW:**   **UPPER:**

**===============================================================================================**

**OBSERVATIONS:**

**REFERENCE NUMBER:** 24

**AUTHORS:** Binbay T, Alptekin K, Elbi H, Zagh N, Drukker M, Aksu T, Özkinay F, Onay H and van Os J**.**

**YEAR OF PUBLICATION:** 2012.

**TITLE:** Lifetime Prevalence and Correlates of Schizophrenia and Disorders with Psychotic Symptoms in the General Population of Izmir, Turkey.

**JOURNAL:** *Turkish Journal of Psychiatry, 23*(3), 149-160.

**===============================================================================================**

**STUDY COUNTRY:** Turkey. **STUDY YEAR:** 2008.

**CASE FINDING SETTING:** census, general population.

**SAMPLING:** Multistage sampling, random, by clusters.

**If sampling, indicate Clusters: CL1-CL2-P/CL:** CL1.

**NUMBER OF STAGES:** 1.

**SCREENING INSTRUMENT:** CIDI (Composite International Diagnostic Interview) 2.A.

**RESPONSE % PHASE 1:** 76.5% **RESPONSE % PHASE 2:**

**DIAGNOSTIC INSTRUMENT:** SCID-I (Structure Clinical Interview for DSM-IV).

**CLASSIFICATION OF DISEASES:** DSM- IV.

**DIAGNOSTIC CATEGORIES:** Schizophrenia.

**STUDY AREA:** Izmir (Smyrna), about 5,245,549 inhabitants.

**POPULATION SIZE (denominator):** 4,011 eligible individuals selected at the end.

**AGE RANGE: LOWER:** 15. **UPPER:** 64.

**STUDY QUALITY:** 13.

**===============================================================================================**

**NUMBER OF ESTIMATES:** 30.

**PREVALENCE RATE (indicated period: point, 12-month, lifetime)**

**PREVALENCE RATE (Lifetime):** 7.48 per 1,000 inhabitants. **STANDARD ERROR: CI: LOW:** 4.80 **UPPER:** 14.10

**MEN:** 9.50 per 1,000 inhabitants. **STANDARD ERROR: CI: LOW:** 4.90 **UPPER:** 14.10

**WOMEN:** 6.00 per 1,000 inhabitants. **STANDARD ERROR: CI: LOW:** 2.90 **UPPER:** 9.20

**===============================================================================================**

**OBSERVATIONS:**

**REFERENCE NUMBER:** 25

**AUTHORS:** Chien I, Chou Y, Lin C, Bih S and Chou P.

**YEAR OF PUBLICATION:** 2004.

**TITLE:** Prevalence of Psychiatric Disorders Among National Health Insurance Enrollees in Taiwan.

**JOURNAL:** *Psychiatry and clinical Neurosciences, 58*(6), 611-618.

**===============================================================================================**

**STUDY COUNTRY:** Taiwan.  **STUDY YEAR:** 2000.

**CASE FINDING SETTING:** census, general population.

**SAMPLING:** random stratified.

**If sampling, indicate Clusters: CL1-CL2-P/CL:**

**NUMBER OF STAGES:** 1.

**SCREENING INSTRUMENT:**

**RESPONSE % PHASE 1: RESPONSE % PHASE 2:**

**DIAGNOSTIC INSTRUMENT:** clinical.

**CLASSIFICATION OF DISEASES:** ICD-9.

**DIAGNOSTIC CATEGORIES:** Schizophrenia.

**STUDY AREA:** Taiwan.  **POPULATION SIZE (denominator):** 137,914.

**AGE RANGE: LOWER:** 18. **UPPER:** 90.

**STUDY QUALITY:** 12.

**===============================================================================================**

**NUMBER OF ESTIMATES:** 607.

**PREVALENCE RATE (indicated period: point, 12-month, lifetime)**

**PREVALENCE RATE (12-month):** 4.40 per 1,000 inhabitants. **STANDARD ERROR: CI: LOW:**  **UPPER:**

**MEN:** 4.90 per 1,000 inhabitants. **STANDARD ERROR: CI: LOW: UPPER:**

**WOMEN:** 4.00 per 1,000 inhabitants. **STANDARD ERROR: CI: LOW: UPPER:**

**===============================================================================================**

**OBSERVATIONS:**

**REFERENCE NUMBER:** 26

**AUTHORS:** Cho M, Kim J, Jeon H, Suh T, Chung I, Hong J, Bae J, Lee D, Park J, Cho S and Lee C and Hahm B.

**YEAR OF PUBLICATION:** 2007.

**TITLE:** Lifetime and 12-Month Prevalence of DSM-IV Psychiatric Disorders Among Korean Adults.

**JOURNAL:** *The Journal of Nervous and Mental Disease,* *195*(3), 203-210.

**===============================================================================================**

**STUDY COUNTRY:** Korea.  **STUDY YEAR:** 2001.

**CASE FINDING SETTING:** census, general population.

**SAMPLING:** nonrandom.

**If sampling, indicate Clusters: CL1-CL2-P/CL:**

**NUMBER OF STAGES:** 1.

**SCREENING INSTRUMENT:**

**RESPONSE % PHASE 1:** 79.8%. **RESPONSE % PHASE 2:**

**DIAGNOSTIC INSTRUMENT:** CIDI (Composite International Diagnostic Interview).

**CLASSIFICATION OF DISEASES:** DSM-IV.

**DIAGNOSTIC CATEGORIES:** Schizophrenia.

**STUDY AREA:** Korea. **POPULATION SIZE (denominator):** 6,275.

**AGE RANGE: LOWER:** 18. **UPPER:** 64.

**STUDY QUALITY:** 13.

**===============================================================================================**

**NUMBER OF ESTIMATES:** 13.

**PREVALENCE RATE (indicated period: point, 12-month, lifetime)**

**PREVALENCE RATE (12-month):** 2.07 per 1,000 inhabitants. **STANDARD ERROR: CI: LOW:**  **UPPER:**

**PREVALENCE RATE (Lifetime):** 2.07 per 1,000 inhabitants. **STANDARD ERROR: CI: LOW:**  **UPPER:**

**MEN:** 12-month (1.00) / lifetime (1.00) per 1,000 inhabitants. **STANDARD ERROR: CI: LOW: UPPER:**

**WOMEN:** 12-month (2.00) / lifetime (2.00) per 1,000 inhabitants. **STANDARD ERROR: CI: LOW: UPPER:**

**===============================================================================================**

**OBSERVATIONS:**

**REFERENCE NUMBER:** 27

**AUTHORS:** Clayer J, McFarlane A, Bookless C, Air T, Wright G and Czechowicz A.

**YEAR OF PUBLICATION:** 1995.

**TITLE:** Prevalence of psychiatric disorders in rural South Australia.

**JOURNAL:** *The* *Medical Journal of Australia, 163*(3), 124-125, 128-129.

**===============================================================================================**

**STUDY COUNTRY:** Australia.  **STUDY YEAR:** 1991.

**CASE FINDING SETTING:** census, general population.

**SAMPLING:** cluster.

**If sampling, indicate Clusters: CL1-CL2-P/CL:**

**NUMBER OF STAGES:** 1.

**SCREENING INSTRUMENT:**

**RESPONSE % PHASE 1: RESPONSE % PHASE 2:**

**DIAGNOSTIC INSTRUMENT:** DISSI (Diagnostic Interview Schedule computer screening interview).

**CLASSIFICATION OF DISEASES:** DSM-III.

**DIAGNOSTIC CATEGORIES:** Schizophrenia.

**STUDY AREA:** Australia. **POPULATION SIZE (denominator):** 1,009.

**AGE RANGE: LOWER:** 18. **UPPER:** 90.

**STUDY QUALITY:** 13.

**===============================================================================================**

**NUMBER OF ESTIMATES:** 8.

**PREVALENCE RATE (indicated period: point, 12-month, lifetime)**

**PREVALENCE RATE (12-month):** 7.93 per 1,000 inhabitants. **STANDARD ERROR: CI: LOW:**  **UPPER:**

**MEN:** 2.00 per 1,000 inhabitants. **STANDARD ERROR: CI: LOW: UPPER:**

**WOMEN:** 13.00 per 1,000 inhabitants. **STANDARD ERROR: CI: LOW: UPPER:**

**===============================================================================================**

**OBSERVATIONS:**

**REFERENCE NUMBER:** 28

**AUTHORS:** Cohidon C, Imbernon E and Gorldberg M.

**YEAR OF PUBLICATION:** 2009.

**TITLE:** Prevalence of Common Mental Disorders and Their Work Consequences in France, According to Occupational Category.

**JOURNAL:** *American Journal of Industrial Medicine, 52*(2), 141-152.

**===============================================================================================**

**STUDY COUNTRY:** France.  **STUDY YEAR:** 2003.

**CASE FINDING SETTING:** census, general population.

**SAMPLING:** nonrandom.

**If sampling, indicate Clusters: CL1-CL2-P/CL:**

**NUMBER OF STAGES:** 1.

**SCREENING INSTRUMENT:**

**RESPONSE % PHASE 1: RESPONSE % PHASE 2:**

**DIAGNOSTIC INSTRUMENT:** MINI (Mini-International Neuropsychiatric Interview).

**CLASSIFICATION OF DISEASES:** ICD-10.

**DIAGNOSTIC CATEGORIES:** Probable psychotic disorder.

**STUDY AREA:** France. **POPULATION SIZE (denominator):** 40,157.

**AGE RANGE: LOWER:** 18. **UPPER:** 90.

**STUDY QUALITY:** 7.

**===============================================================================================**

**NUMBER OF ESTIMATES:** 1084.

**PREVALENCE RATE (indicated period: point, 12-month, lifetime)**

**PREVALENCE RATE (Lifetime):** 26.99 per 1,000 inhabitants. **STANDARD ERROR: CI: LOW:**  **UPPER:**

**MEN:** 29.50 per 1,000 inhabitants. **STANDARD ERROR: CI: LOW: UPPER:**

**WOMEN:** 24.80 per 1,000 inhabitants. **STANDARD ERROR: CI: LOW: UPPER:**

**===============================================================================================**

**OBSERVATIONS:**

**REFERENCE NUMBER:** 29

**AUTHORS:** Díaz-Cruz F, Bethencourt Juan and Peñate W.

**YEAR OF PUBLICATION:** 2004.

**TITLE:** Prevalencia de los trastornos mentales en la isla de Tenerife.

**JOURNAL:** *Revista de la Asociación Española de Neuropsiquiatría, 23*(90), 3082-3099.

**===============================================================================================**

**STUDY COUNTRY:** Spain.  **STUDY YEAR:** 1999.

**CASE FINDING SETTING:** census, general population.

**SAMPLING:** nonrandom.

**If sampling, indicate Clusters: CL1-CL2-P/CL:**

**NUMBER OF STAGES:** 2.

**SCREENING INSTRUMENT:**

**RESPONSE % PHASE 1: RESPONSE % PHASE 2:**

**DIAGNOSTIC INSTRUMENT:** CIDI (Composite International Diagnostic Interview).

**CLASSIFICATION OF DISEASES:** DSM-IV.

**DIAGNOSTIC CATEGORIES:** non-affective psychoses.

**STUDY AREA:** Spain. **POPULATION SIZE (denominator):** 800.

**AGE RANGE: LOWER:** 18. **UPPER:** 90.

**STUDY QUALITY:** 12.

**===============================================================================================**

**NUMBER OF ESTIMATES:** 2.

**PREVALENCE RATE (indicated period: point, 12-month, lifetime)**

**PREVALENCE RATE (Point):** 2.50 per 1,000 inhabitants. **STANDARD ERROR: CI: LOW:**  **UPPER:**

**MEN:** 29.50 per 1,000 inhabitants. **STANDARD ERROR: CI: LOW: UPPER:**

**WOMEN:** 10.00 per 1,000 inhabitants. **STANDARD ERROR: CI: LOW: UPPER:**

**===============================================================================================**

**OBSERVATIONS:**

**REFERENCE NUMBER:** 30

**AUTHORS:** Díaz-Martínez A, Díaz-Martínez R, Osornio-Rojo A and Rascón-Gasca M.

**YEAR OF PUBLICATION:** 2003.

**TITLE:** La salud mental en el municipio de Querétaro: un modelo de investigación psiquiátrica en la comunidad.

**JOURNAL:** *Gaceta Médica de México, 139*(2), 101-107.

**===============================================================================================**

**STUDY COUNTRY:** Mexico.  **STUDY YEAR:** 2001.

**CASE FINDING SETTING:** census, general population.

**SAMPLING:** nonrandom.

**If sampling, indicate Clusters: CL1-CL2-P/CL:**

**NUMBER OF STAGES:** 1.

**SCREENING INSTRUMENT:**

**RESPONSE % PHASE 1: RESPONSE % PHASE 2:**

**DIAGNOSTIC INSTRUMENT:** CIDI (Composite International Diagnostic Interview).

**CLASSIFICATION OF DISEASES:** ICD-10.

**DIAGNOSTIC CATEGORIES:** schizophrenia.

**STUDY AREA:** Mexico. **POPULATION SIZE (denominator):** 608.

**AGE RANGE: LOWER:** 15. **UPPER:** 65.

**STUDY QUALITY:** 7.

**===============================================================================================**

**NUMBER OF ESTIMATES:** 12.

**PREVALENCE RATE (indicated period: point, 12-month, lifetime)**

**PREVALENCE RATE (12-month):** 19.74 per 1,000 inhabitants. **STANDARD ERROR: CI: LOW:**  **UPPER:**

**MEN:** 10.00 per 1,000 inhabitants. **STANDARD ERROR: CI: LOW: UPPER:**

**WOMEN:** 10.00 per 1,000 inhabitants. **STANDARD ERROR: CI: LOW: UPPER:**

**===============================================================================================**

**OBSERVATIONS:**

**REFERENCE NUMBER:** 31

**AUTHORS:** Dourado A, Azevedo M, Macedo A, Coelho I, Valente J, Soares M, Luis A, Pato C and Pato M.

**YEAR OF PUBLICATION:** 2001.

**TITLE:** A look at the influence of Genetics in Psychiatry: Reduced prevalence of psychoses in Santa Maria Island, Azores, Portugal.

**JOURNAL:** *American Journal of Medical Genetics ­ Neuropsychiatric Genetics, 96*(4), 513.

**===============================================================================================**

**STUDY COUNTRY:** Azores.  **STUDY YEAR:** 1998.

**CASE FINDING SETTING:** attended population in Mental Health Services + Primary care + Social services.

**SAMPLING:** nonrandom.

**If sampling, indicate Clusters: CL1-CL2-P/CL:**

**NUMBER OF STAGES:** 1.

**SCREENING INSTRUMENT:** OPCRIT (Operational Criteria Checklist for Psychosis).

**RESPONSE % PHASE 1: RESPONSE % PHASE 2:**

**DIAGNOSTIC INSTRUMENT:** DIGS (Diagnostic Interview for Genetic Studies).

**CLASSIFICATION OF DISEASES:** DSM-III-R + ICD-10.

**DIAGNOSTIC CATEGORIES:** Schizophrenia.

**STUDY AREA:** Santa Maria Island. **POPULATION SIZE (denominator):** 4,332.

**AGE RANGE: LOWER:** 15. **UPPER:** Unlimited.

**STUDY QUALITY:** 10.

**===============================================================================================**

**NUMBER OF ESTIMATES:** 14.

**PREVALENCE RATE (indicated period: point, 12-month, lifetime)**

**PREVALENCE RATE (Lifetime):** 2.40 per 1,000 inhabitants. **STANDARD ERROR: CI: LOW:**  **UPPER:**

**MEN:** 2.70 per 1,000 inhabitants. **STANDARD ERROR: CI: LOW: UPPER:**

**WOMEN:** 2.00 per 1,000 inhabitants. **STANDARD ERROR: CI: LOW: UPPER:**

**===============================================================================================**

**OBSERVATIONS:**

**REFERENCE NUMBER:** 32

**AUTHORS:** Faravelli C, Abrardi L, Bartolozzi D, Cecchi C, Cosci F, D'Adamo D, Lo Iacono B, Ravaldi C, Scarpato MA, Truglia E, Rosi S. .

**YEAR OF PUBLICATION:** 2004.

**TITLE:** The Sesto Fiorentino Study: Background, Methods and Preliminary Results. Lifetime Prevalence of Psychiatric Disorders in an Italian Community Sample Using Clinical Interviewers.

**JOURNAL:** *Psychothery and Psychosomatics, 73*(4), 216-225.

**===============================================================================================**

**STUDY COUNTRY:** Italy.  **STUDY YEAR:** 2001.

**CASE FINDING SETTING:** census, general population.

**SAMPLING:** random stratified.

**If sampling, indicate Clusters: CL1-CL2-P/CL:**

**NUMBER OF STAGES:** 3.

**SCREENING INSTRUMENT:**

**RESPONSE % PHASE 1: RESPONSE % PHASE 2:**

**DIAGNOSTIC INSTRUMENT:** SCID (Structure Clinical Interview for DSM-IV).

**CLASSIFICATION OF DISEASES:** DSM-IV.

**DIAGNOSTIC CATEGORIES:** non-affective psychoses.

**STUDY AREA:** Italy. **POPULATION SIZE (denominator):** 2,363.

**AGE RANGE: LOWER:** 14. **UPPER:** 90.

**STUDY QUALITY:** 14.

**===============================================================================================**

**NUMBER OF ESTIMATES:** 17.

**PREVALENCE RATE (indicated period: point, 12-month, lifetime)**

**PREVALENCE RATE (Lifetime):** 7.19 per 1,000 inhabitants. **STANDARD ERROR: CI: LOW:**  **UPPER:**

**MEN:** 5.00 per 1,000 inhabitants. **STANDARD ERROR: CI: LOW: UPPER:**

**WOMEN:** 9.00 per 1,000 inhabitants. **STANDARD ERROR: CI: LOW: UPPER:**

**===============================================================================================**

**OBSERVATIONS:**

**REFERENCE NUMBER:** 33

**AUTHORS:** Fekadu A, Shibre T, Alem A, Kebede D, Kebreab, Negash A and Owen M**.**

**YEAR OF PUBLICATION:** 2004.

**TITLE:** Bipolar disorder among an isolated island community in Ethiopia.

**JOURNAL:** *Journal of Affective Disorders, 80*(1), 1–10.

**===============================================================================================**

**STUDY COUNTRY:** Ethiopia.  **STUDY YEAR:** 1998.

**CASE FINDING SETTING:** census, general population.

**SAMPLING:** Simple random.

**If sampling, indicate Clusters: CL1-CL2-P/CL:**

**NUMBER OF STAGES:** 3.

**SCREENING INSTRUMENT:** CIDI (Composite International Diagnostic Interview) 2.1.

**RESPONSE % PHASE 1:** 74.1%. **RESPONSE % PHASE 2:** 91.2%.

**DIAGNOSTIC INSTRUMENT:** SCAN (Schedules for Clinical Assessment in Neuropsychiatry).

**CLASSIFICATION OF DISEASES:** ICD-10.

**DIAGNOSTIC CATEGORIES:** Schizophrenia.

**STUDY AREA:** Zeway. **POPULATION SIZE (denominator):** 2,281.

**AGE RANGE: LOWER:** 15. **UPPER:** Unlimited.

**STUDY QUALITY:** 10.

**===============================================================================================**

**NUMBER OF ESTIMATES:** 1.

**PREVALENCE RATE (indicated period: point, 12-month, lifetime)**

**PREVALENCE RATE (Point):** 0.44 per 1,000 inhabitants. **STANDARD ERROR: CI: LOW:**  **UPPER:**

**MEN:** **STANDARD ERROR: CI: LOW: UPPER:**

**WOMEN: STANDARD ERROR: CI: LOW: UPPER:**

**===============================================================================================**

**OBSERVATIONS:**

**REFERENCE NUMBER:** 34

**AUTHORS:** Fors B, Isacson D, Bingefors K and Widerlöw B.

**YEAR OF PUBLICATION:** 2007.

**TITLE:** Mortality among persons with schizophrenia in Sweden: An epidemiological study.

**JOURNAL:** *Nordic Journal of Psychiatry, 61*(4), 252–259.

**===============================================================================================**

**STUDY COUNTRY:** Sweden.  **STUDY YEAR:** 1991.

**CASE FINDING SETTING:** attended population in Mental Health Services + Primary Care + Social Services.

**SAMPLING:** nonrandom.

**If sampling, indicate Clusters: CL1-CL2-P/CL:**

**NUMBER OF STAGES:** 1.

**SCREENING INSTRUMENT:**

**RESPONSE % PHASE 1: RESPONSE % PHASE 2:**

**DIAGNOSTIC INSTRUMENT:** clinical.

**CLASSIFICATION OF DISEASES:** DSM-III-R + DSM-IV.

**DIAGNOSTIC CATEGORIES:** Schizophrenia.

**STUDY AREA:** Uppsala. **POPULATION SIZE (denominator):** 64,041.

**AGE RANGE: LOWER:** 18. **UPPER:** Unlimited.

**STUDY QUALITY:** 10.

**===============================================================================================**

**NUMBER OF ESTIMATES:** 237.

**PREVALENCE RATE (indicated period: point, 12-month, lifetime)**

**PREVALENCE RATE (12-month):** 3.70 per 1,000 inhabitants. **STANDARD ERROR: CI: LOW:**  **UPPER:**

**MEN:** 4.20 per 1,000 inhabitants. **STANDARD ERROR: CI: LOW: UPPER:**

**WOMEN:** 3.30 per 1,000 inhabitants.  **STANDARD ERROR: CI: LOW: UPPER:**

**===============================================================================================**

**OBSERVATIONS:**

**REFERENCE NUMBER:** 35

**AUTHORS:** Gigantesco A, Palumbo G, Mirabella F, Pettinelli M. and Morosini P.

**YEAR OF PUBLICATION:** 2006.

**TITLE:** Prevalence of Psychiatric Disorders in an Italian Town: Low Prevalence Conﬁrmed with Two Different Interviews.

**JOURNAL:** *Psychotherapy Psychosomatics, 75*(3), 170-176.

**===============================================================================================**

**STUDY COUNTRY:** Italy.  **STUDY YEAR:** 2000.

**CASE FINDING SETTING:** census, general population.

**SAMPLING:** random stratified.

**If sampling, indicate Clusters: CL1-CL2-P/CL:**

**NUMBER OF STAGES:** 1.

**SCREENING INSTRUMENT:**

**RESPONSE % PHASE 1: RESPONSE % PHASE 2:**

**DIAGNOSTIC INSTRUMENT:** CIDI (Composite International Diagnostic Interview).

**CLASSIFICATION OF DISEASES:** ICD-10.

**DIAGNOSTIC CATEGORIES:** probable psychotic disorder.

**STUDY AREA:** Italy. **POPULATION SIZE (denominator):** 267.

**AGE RANGE: LOWER:** 18. **UPPER:** 90.

**STUDY QUALITY:** 15.

**===============================================================================================**

**NUMBER OF ESTIMATES:** 1 (point) + 1 (12-month) + 2 (lifetime) = 3.

**PREVALENCE RATE (indicated period: point, 12-month, lifetime)**

**PREVALENCE RATE (Point):** 3.75 per 1,000 inhabitants. **STANDARD ERROR: CI: LOW:** 0.00 **UPPER:** 21.00

**PREVALENCE RATE (12-month):** 3.75 per 1,000 inhabitants. **STANDARD ERROR: CI: LOW:** 0.00 **UPPER:** 21.00

**PREVALENCE RATE (Lifetime):** 7.49 per 1,000 inhabitants. **STANDARD ERROR: CI: LOW:** 3.00 **UPPER:** 10.00

**MEN:** 8.00 (point) / 8.00 (12-month) / 16.00 (lifetime) per 1,000 inhabitants. **STANDARD ERROR: CI: LOW: UPPER:**

**WOMEN:**  **STANDARD ERROR: CI: LOW: UPPER:**

**===============================================================================================**

**OBSERVATIONS:**

**REFERENCE NUMBER:** 36

**AUTHORS:** Goldner EM, Jones W and Waraich P.

**YEAR OF PUBLICATION:** 2003.

**TITLE:** Using administrative Data to Analyze the Prevalence and Distribution of Schizophrenic Disorders.

**JOURNAL:** *Psychiatric Services, 54*(7), 1017-1021.

**===============================================================================================**

**STUDY COUNTRY:** Canada.  **STUDY YEAR:** 1999.

**CASE FINDING SETTING:** attended population in Mental Health Services + Primary Care + Social Services.

**SAMPLING:** nonrandom.

**If sampling, indicate Clusters: CL1-CL2-P/CL:**

**NUMBER OF STAGES:** 1.

**SCREENING INSTRUMENT:**

**RESPONSE % PHASE 1: RESPONSE % PHASE 2:**

**DIAGNOSTIC INSTRUMENT:** clinical.

**CLASSIFICATION OF DISEASES:** ICD-9 + DSM-IV.

**DIAGNOSTIC CATEGORIES:** Schizophrenia.

**STUDY AREA:** British Columbia. **POPULATION SIZE (denominator):** 11,516.

**AGE RANGE: LOWER:** 15. **UPPER:** 65.

**STUDY QUALITY:** 8.

**===============================================================================================**

**NUMBER OF ESTIMATES:** 48.

**PREVALENCE RATE (indicated period: point, 12-month, lifetime)**

**PREVALENCE RATE (12-month):** 4.17 per 1,000 inhabitants. **STANDARD ERROR: CI: LOW: UPPER:**

**MEN:** **STANDARD ERROR: CI: LOW: UPPER:**

**WOMEN:**  **STANDARD ERROR: CI: LOW: UPPER:**

**===============================================================================================**

**OBSERVATIONS:**

**REFERENCE NUMBER:** 37

**AUTHORS:** Gureje O, Olowosegun O, Adebayo K and Stein D.

**YEAR OF PUBLICATION:** 2010.

**TITLE:** The prevalence and profile of non-affective psychosis in the Nigerian Survey of Mental Health and Wellbeing.

**JOURNAL:** *Official Journal of the World Psychiatric Association, 9*(1), 50-55.

**===============================================================================================**

**STUDY COUNTRY:** Nigeria.  **STUDY YEAR:** 2003.

**CASE FINDING SETTING:** census, general population.

**SAMPLING:** Random stratified.

**If sampling, indicate Clusters: CL1-CL2-P/CL:**

**NUMBER OF STAGES:** 2.

**SCREENING INSTRUMENT:**

**RESPONSE % PHASE 1: RESPONSE % PHASE 2:**

**DIAGNOSTIC INSTRUMENT:** CIDI (Composite International Diagnostic Interview).

**CLASSIFICATION OF DISEASES:** DSM-IV.

**DIAGNOSTIC CATEGORIES:** non-affective psychoses.

**STUDY AREA:** Nigeria. **POPULATION SIZE (denominator):** 4,985.

**AGE RANGE: LOWER:** 18. **UPPER:** Unlimited.

**STUDY QUALITY:** 11.

**===============================================================================================**

**NUMBER OF ESTIMATES:** 105 (lifetime) + 55 (12-month)

**PREVALENCE RATE (indicated period: point, 12-month, lifetime)**

**PREVALENCE RATE (12-month):** 11.03 per 1,000 inhabitants. **STANDARD ERROR: CI: LOW: UPPER:**

**PREVALENCE RATE (Lifetime):** 21.06 per 1,000 inhabitants. **STANDARD ERROR: CI: LOW: UPPER:**

**MEN:** **STANDARD ERROR: CI: LOW: UPPER:**

**WOMEN:**  **STANDARD ERROR: CI: LOW: UPPER:**

**===============================================================================================**

**OBSERVATIONS:**

**REFERENCE NUMBER:** 38

**AUTHORS:** Hosain GM, Chatterjee N, Ara N and Islam T.

**YEAR OF PUBLICATION:** 2007.

**TITLE:** Prevalence, pattern and determinants of mental disorders in rural Bangladesh.

**JOURNAL:** *Public Health, 121*(1), 18-24.

**===============================================================================================**

**STUDY COUNTRY:** Bangladesh.  **STUDY YEAR:** 2001.

**CASE FINDING SETTING:** census, general population.

**SAMPLING:** nonrandom.

**If sampling, indicate Clusters: CL1-CL2-P/CL:**

**NUMBER OF STAGES:** 2.

**SCREENING INSTRUMENT:**

**RESPONSE % PHASE 1: RESPONSE % PHASE 2:**

**DIAGNOSTIC INSTRUMENT:** clinical.

**CLASSIFICATION OF DISEASES:** DSM-IV.

**DIAGNOSTIC CATEGORIES:** non-affective psychoses.

**STUDY AREA:** rural Bangladesh. **POPULATION SIZE (denominator):** 766.

**AGE RANGE: LOWER:** 18. **UPPER:** 60.

**STUDY QUALITY:** 10.

**===============================================================================================**

**NUMBER OF ESTIMATES:** 9.

**PREVALENCE RATE (indicated period: point, 12-month, lifetime)**

**PREVALENCE RATE (Point):** 11.75 per 1,000 inhabitants. **STANDARD ERROR: CI: LOW: UPPER:**

**MEN:** 8.00 per 1,000 inhabitants. **STANDARD ERROR: CI: LOW: UPPER:**

**WOMEN:** 14.00 per 1,000 inhabitants. **STANDARD ERROR: CI: LOW: UPPER:**

**===============================================================================================**

**OBSERVATIONS:**

**REFERENCE NUMBER:** 39

**AUTHORS:** Hovatta I, Terwilliger JD, Lichtermann D, Mäkikyrö T, Suvisaari J, Peltonen L. and Lönnqvist J.

**YEAR OF PUBLICATION:** 1997.

**TITLE:** Schizophrenia in the Genetic Isolate of Finland.

**JOURNAL:** *American Journal of Medical Genetics, 74*(4), 353-360.

**===============================================================================================**

**STUDY COUNTRY:** Finland. **STUDY YEAR:** 1991.

**CASE FINDING SETTING:** attended population in Mental Health Services + Primary Care.

**SAMPLING:** simple random.

**If sampling, indicate Clusters: CL1-CL2-P/CL:**

**NUMBER OF STAGES:** 1.

**SCREENING INSTRUMENT:**

**RESPONSE % PHASE 1: RESPONSE % PHASE 2:**

**DIAGNOSTIC INSTRUMENT:** clinical.

**CLASSIFICATION OF DISEASES:** DSM-III-R.

**DIAGNOSTIC CATEGORIES:** Schizophrenia and related disorders

**STUDY AREA:** Finland. **POPULATION SIZE (denominator):** 4,998,478 (Finland).

**AGE RANGE: LOWER:** 35. **UPPER:** 54.

**STUDY QUALITY:** 12.

**===============================================================================================**

**NUMBER OF ESTIMATES:** 29,091.

**PREVALENCE RATE (indicated period: point, 12-month, lifetime)**

**PREVALENCE RATE (Lifetime):** 5.82 per 1,000 inhabitants. **STANDARD ERROR: CI: LOW: UPPER:**

**MEN:**  **STANDARD ERROR: CI: LOW: UPPER:**

**WOMEN: STANDARD ERROR: CI: LOW: UPPER:**

**===============================================================================================**

**OBSERVATIONS:**

**REFERENCE NUMBER:** 40

**AUTHORS:** Jablensky A, McGrath J, Herrman H, Castle D, Gureje O, Evans M, Carr V, Morgan V, Korten A and Harvey C.

**YEAR OF PUBLICATION:** 2000.

**TITLE:** Psychotic disorders in urban areas: an overview of the Study on Low Prevalence Disorders.

**JOURNAL:** *Australian & New Zealand Journal of Psychiatry, 34*(S2), 26-34.

**===============================================================================================**

**STUDY COUNTRY:** Australia. **STUDY YEAR:** 1997.

**CASE FINDING SETTING:** attended population in Mental Health Services + Primary Care + Social Services.

**SAMPLING:** simple random.

**If sampling, indicate Clusters: CL1-CL2-P/CL:**

**NUMBER OF STAGES:** 2.

**SCREENING INSTRUMENT:**

**RESPONSE % PHASE 1: RESPONSE % PHASE 2:**

**DIAGNOSTIC INSTRUMENT:** OPCRIT (Operational Criteria Checklist for Psychosis) + SCAN (Schedules for Clinical Assessment in Neuropsychiatry).

**CLASSIFICATION OF DISEASES:** ICD-10.

**DIAGNOSTIC CATEGORIES:** probable psychotic disorder.

**STUDY AREA:** Australia. **POPULATION SIZE (denominator):** 980.

**AGE RANGE: LOWER:** 18. **UPPER:** 65.

**STUDY QUALITY:** 13.

**===============================================================================================**

**NUMBER OF ESTIMATES:** 5.

**PREVALENCE RATE (indicated period: point, 12-month, lifetime)**

**PREVALENCE RATE (Point):** 5.10 per 1,000 inhabitants. **STANDARD ERROR: CI: LOW:** 3.90 **UPPER:** 6.90

**MEN:**  5.20 per 1,000 inhabitants. **STANDARD ERROR: CI: LOW: UPPER:**

**WOMEN:** 4.10 per 1,000 inhabitants.  **STANDARD ERROR: CI: LOW: UPPER:**

**===============================================================================================**

**OBSERVATIONS:**

**REFERENCE NUMBER:** 41

**AUTHORS:** Jeffreys SE, Harvey CA, McNaught AS, Quayle AS, King MB and Bird AS.

**YEAR OF PUBLICATION:** 1997.

**TITLE:** The Hampstead Schizophrenia Survey 1991. I: Prevalence and Service Use Comparisons in an Inner London Health Authority, 1986-1991.

**JOURNAL:** *The British Journal of Psychiatry, 170,* 301-306.

**===============================================================================================**

**STUDY COUNTRY:** United Kingdom. **STUDY YEAR:** 1991.

**CASE FINDING SETTING:** attended population in Mental Health Services + Primary Care + Social Services.

**SAMPLING:** simple random.

**If sampling, indicate Clusters: CL1-CL2-P/CL:**

**NUMBER OF STAGES:** 1.

**SCREENING INSTRUMENT:**

**RESPONSE % PHASE 1: RESPONSE % PHASE 2:**

**DIAGNOSTIC INSTRUMENT:** SCAN (Schedules for Clinical Assessment in Neuropsychiatry).

**CLASSIFICATION OF DISEASES:** DSM-III-R.

**DIAGNOSTIC CATEGORIES:** probable psychotic disorder.

**STUDY AREA:** United Kingdom. **POPULATION SIZE (denominator):** 115,294.

**AGE RANGE: LOWER:** 15. **UPPER:** 54.

**STUDY QUALITY:** 14.

**===============================================================================================**

**NUMBER OF ESTIMATES:** 588.

**PREVALENCE RATE (indicated period: point, 12-month, lifetime)**

**PREVALENCE RATE (Point):** 5.10 per 1,000 inhabitants. **STANDARD ERROR: CI: LOW:**  **UPPER:**

**MEN:**  **STANDARD ERROR: CI: LOW: UPPER:**

**WOMEN:** **STANDARD ERROR: CI: LOW: UPPER:**

**===============================================================================================**

**OBSERVATIONS:**

**REFERENCE NUMBER:** 42

**AUTHORS:** Jenkins R, Lewis G, Bebbington P, Brugha T, Farrell M, Gill B and Meltzer H.

**YEAR OF PUBLICATION:** 1997.

**TITLE:** The National Psychiatric Morbidity Surveys of Great Britain – initial findings from the Household Survey.

**JOURNAL:** *The British Journal of Psychiatry, 170,* 301-306.

**===============================================================================================**

**STUDY COUNTRY:** United Kingdom. **STUDY YEAR:** 1994.

**CASE FINDING SETTING:** census, general population.

**SAMPLING:** nonrandom.

**If sampling, indicate Clusters: CL1-CL2-P/CL:**

**NUMBER OF STAGES:** 2.

**SCREENING INSTRUMENT:**

**RESPONSE % PHASE 1:** 79.4%.  **RESPONSE % PHASE 2:**

**DIAGNOSTIC INSTRUMENT:** SCAN (Schedules for Clinical Assessment in Neuropsychiatry).

**CLASSIFICATION OF DISEASES:** ICD-10.

**DIAGNOSTIC CATEGORIES:** non-affective psychoses.

**STUDY AREA:** with the exception of Highlands and Islands of Scotland. **POPULATION SIZE (denominator):** 10,108

**AGE RANGE: LOWER:** 15. **UPPER:** 65.

**STUDY QUALITY:** 15.

**===============================================================================================**

**NUMBER OF ESTIMATES:** 40.

**PREVALENCE RATE (indicated period: point, 12-month, lifetime)**

**PREVALENCE RATE (12-month):** 3.96 per 1,000 inhabitants. **STANDARD ERROR: CI: LOW:** 2.00 **UPPER:** 6.00

**MEN:** 4.00 per 1,000 inhabitants. **STANDARD ERROR: CI: LOW: UPPER:**

**WOMEN:** 4.00 per 1,000 inhabitants. **STANDARD ERROR: CI: LOW: UPPER:**

**===============================================================================================**

**OBSERVATIONS:**

**REFERENCE NUMBER:** 43

**AUTHORS:** Jörgensen L, Allebeck P and Dalman C.

**YEAR OF PUBLICATION:** 2013.

**TITLE:** Prevalence of psychoses in Stockholm Country-A population-based study using comprehensive healthcare registers.

**JOURNAL:** *Nordic Journal of Psychiatry, 68*(1), 60-65.

**===============================================================================================**

**STUDY COUNTRY:** Sweden. **STUDY YEAR:** 2005.

**CASE FINDING SETTING:** population attended in Mental Health Services + Primary Care.

**SAMPLING:** nonrandom.

**If sampling, indicate Clusters: CL1-CL2-P/CL:**

**NUMBER OF STAGES:** 1.

**SCREENING INSTRUMENT:**

**RESPONSE % PHASE 1:**   **RESPONSE % PHASE 2:**

**DIAGNOSTIC INSTRUMENT:** clinical.

**CLASSIFICATION OF DISEASES:** ICD-10.

**DIAGNOSTIC CATEGORIES:** schizophrenia.

**STUDY AREA:** Stockholm. **POPULATION SIZE (denominator):** 946,381.

**AGE RANGE: LOWER:** 18. **UPPER:** 64.

**STUDY QUALITY:** 10.

**===============================================================================================**

**NUMBER OF ESTIMATES:** 3,502.

**PREVALENCE RATE (indicated period: point, 12-month, lifetime)**

**PREVALENCE RATE (12-month):** 3.70 per 1,000 inhabitants. **STANDARD ERROR: CI: LOW:** 2.90 **UPPER:** 4.40

**MEN:** 4.20 per 1,000 inhabitants. **STANDARD ERROR: CI: LOW:** 4.00 **UPPER:** 4.40

**WOMEN:** 3.10 per 1,000 inhabitants. **STANDARD ERROR: CI: LOW:** 2.90 **UPPER:** 3.20

**===============================================================================================**

**OBSERVATIONS:**

**REFERENCE NUMBER:** 44

**AUTHORS:** Kake TR, Arnold R and Ellis P.

**YEAR OF PUBLICATION:** 2008.

**TITLE:** Estimating the prevalence of schizophrenia amongst New Zealand Mäori: a capture-recapture approach.

**JOURNAL:** *Australian & New Zealand Journal of Psychiatry, 42*(11), 941-949.

**===============================================================================================**

**STUDY COUNTRY:** Australia. **STUDY YEAR:** 2003.

**CASE FINDING SETTING:** attended population in Mental Health Services.

**SAMPLING:** Simple random.

**If sampling, indicate Clusters: CL1-CL2-P/CL:**

**NUMBER OF STAGES:** 1.

**SCREENING INSTRUMENT:**

**RESPONSE % PHASE 1:**   **RESPONSE % PHASE 2:**

**DIAGNOSTIC INSTRUMENT:** clinical.

**CLASSIFICATION OF DISEASES:** ICD-9.

**DIAGNOSTIC CATEGORIES:** schizophrenia.

**STUDY AREA:** Australia.  **POPULATION SIZE (denominator):** 3,736,269.

**AGE RANGE: LOWER:** 15. **UPPER:** 65.

**STUDY QUALITY:** 10.

**===============================================================================================**

**NUMBER OF ESTIMATES:** 11,956.

**PREVALENCE RATE (indicated period: point, 12-month, lifetime)**

**PREVALENCE RATE (12-month):** 3.20 per 1,000 inhabitants. **STANDARD ERROR: CI: LOW:** 2.60 **UPPER:** 3.80

**MEN:** 3.10 per 1,000 inhabitants. **STANDARD ERROR: CI: LOW:**  **UPPER:**

**WOMEN:** 2.90 per 1,000 inhabitants. **STANDARD ERROR: CI: LOW:**  **UPPER:**

**===============================================================================================**

**OBSERVATIONS:**

**REFERENCE NUMBER:** 45

**AUTHORS:** Kebede D and Alem A.

**YEAR OF PUBLICATION:** 1999.

**TITLE:** Major mental disorders in Addis Ababa, Ethiopia. I. Schizophrenia, schizoaffective and cognitive disorders.

**JOURNAL:** *Acta Psychiatrica Scandinavica Supplementum, 397*, 11-17.

**===============================================================================================**

**STUDY COUNTRY:** Ethiopia. **STUDY YEAR:** 1994.

**CASE FINDING SETTING:** census, general population.

**SAMPLING:** nonrandom.

**If sampling, indicate Clusters: CL1-CL2-P/CL:**

**NUMBER OF STAGES:** 2.

**SCREENING INSTRUMENT:**

**RESPONSE % PHASE 1:**   **RESPONSE % PHASE 2:**

**DIAGNOSTIC INSTRUMENT:** CIDI (Composite International Diagnostic Interview).

**CLASSIFICATION OF DISEASES:** ICD-10.

**DIAGNOSTIC CATEGORIES:** schizophrenia.

**STUDY AREA:** Ethiopia.  **POPULATION SIZE (denominator):** 10,203.

**AGE RANGE: LOWER:** 15. **UPPER:** 90.

**STUDY QUALITY:** 12.

**===============================================================================================**

**NUMBER OF ESTIMATES:** 31.

**PREVALENCE RATE (indicated period: point, 12-month, lifetime)**

**PREVALENCE RATE (Point):** 3.04 per 1,000 inhabitants. **STANDARD ERROR: CI: LOW:**  **UPPER:**

**MEN:** 2.00 (point) per 1,000 inhabitants. **STANDARD ERROR: CI: LOW:**  **UPPER:**

**WOMEN:** 3.00 (point) per 1,000 inhabitants. **STANDARD ERROR: CI: LOW:**  **UPPER:**

**===============================================================================================**

**OBSERVATIONS:**

**REFERENCE NUMBER:** 46

**AUTHORS:** Kebede D, Alem A, Shibre T, Negash A, Fekadu A, Fekadu D, Deyassa N, Jacobsson L and Kullgren G.

**YEAR OF PUBLICATION:** 2003.

**TITLE:** Onset and clinical course of schizophrenia.

**JOURNAL:** *Social Psychiatry and Psychiatric Epidemiology, 38*(11), 625-31.

**===============================================================================================**

**STUDY COUNTRY:** Ethiopia. **STUDY YEAR:** 2001.

**CASE FINDING SETTING:** census, general population.

**SAMPLING:** Simple random.

**If sampling, indicate Clusters: CL1-CL2-P/CL:**

**NUMBER OF STAGES:** 2.

**SCREENING INSTRUMENT:** CIDI (Composite International Diagnostic Interview).

**RESPONSE % PHASE 1:**   **RESPONSE % PHASE 2:**

**DIAGNOSTIC INSTRUMENT:** SCAN (Schedules for Clinical Assessment in Neuropsychiatry).

**CLASSIFICATION OF DISEASES:** ICD-10.

**DIAGNOSTIC CATEGORIES:** schizophrenia.

**STUDY AREA:** Butajira. **POPULATION SIZE (denominator):** 68,378.

**AGE RANGE: LOWER:** 15. **UPPER:** 49.

**STUDY QUALITY:** 11.

**===============================================================================================**

**NUMBER OF ESTIMATES:** 321.

**PREVALENCE RATE (indicated period: point, 12-month, lifetime)**

**PREVALENCE RATE (Lifetime):** 4.69 per 1,000 inhabitants. **STANDARD ERROR: CI: LOW:**  **UPPER:**

**MEN:** **STANDARD ERROR: CI: LOW:**  **UPPER:**

**WOMEN:** **STANDARD ERROR: CI: LOW:**  **UPPER:**

**===============================================================================================**

**OBSERVATIONS:**

**REFERENCE NUMBER:** 47

**AUTHORS:** Kendler KS, Gallagher TJ, Abelson JM and Kessler RC.

**YEAR OF PUBLICATION:** 1996.

**TITLE:** Lifetime Prevalence, Demographic Risk Factors, and Diagnostic Validity of Nonaffective Psychosis as Assessed in a US Community Sample: The National Comorbidity Survey.

**JOURNAL:** *The National Comorbidity Survey. Archives of General Psychiatry, 53*(11), 1022-1031.

**===============================================================================================**

**STUDY COUNTRY:** USA. **STUDY YEAR:** 1992.

**CASE FINDING SETTING:** census, general population.

**SAMPLING:** Nonrandom.

**If sampling, indicate Clusters: CL1-CL2-P/CL:**

**NUMBER OF STAGES:** 2.

**SCREENING INSTRUMENT:**

**RESPONSE % PHASE 1:**   **RESPONSE % PHASE 2:**

**DIAGNOSTIC INSTRUMENT:** CIDI (Composite International Diagnostic Interview).

**CLASSIFICATION OF DISEASES:** DSM-III-R.

**DIAGNOSTIC CATEGORIES:** Schizophrenia and related disorders

**STUDY AREA:** USA.  **POPULATION SIZE (denominator):** 8,098.

**AGE RANGE: LOWER:** 15. **UPPER:** 54.

**STUDY QUALITY:** 14.

**===============================================================================================**

**NUMBER OF ESTIMATES:** 89.

**PREVALENCE RATE (indicated period: point, 12-month, lifetime)**

**PREVALENCE RATE (Lifetime):** 10.99 per 1,000 inhabitants. **STANDARD ERROR: CI: LOW:** 9.00 **UPPER:** 13.00

**MEN:** **STANDARD ERROR: CI: LOW:**  **UPPER:**

**WOMEN:** **STANDARD ERROR: CI: LOW:**  **UPPER:**

**===============================================================================================**

**OBSERVATIONS:**

**REFERENCE NUMBER:** 48

**AUTHORS:** Kessler RC, Birnbaum H, Demler O, Falloon IRH, Gagnon E, Guyer M, Howes MJ, Kendler KS, Shi L, Walters E and Wu EQ.

**YEAR OF PUBLICATION:** 2005.

**TITLE:** The prevalence and correlates of non-affective psychosis in the National Comorbidity Survey Replication (NCS-R).

**JOURNAL:** *Biological Psychiatry, 58*(8), 668-676.

**===============================================================================================**

**STUDY COUNTRY:** USA. **STUDY YEAR:** 2003.

**CASE FINDING SETTING:** census, general population.

**SAMPLING:** Simple random.

**If sampling, indicate Clusters: CL1-CL2-P/CL:**

**NUMBER OF STAGES:** 1.

**SCREENING INSTRUMENT:**

**RESPONSE % PHASE 1:** 70.90%. **RESPONSE % PHASE 2:**

**DIAGNOSTIC INSTRUMENT:** CIDI (Composite International Diagnostic Interview) + SCID (Structure Clinical Interview for DSM-IV).

**CLASSIFICATION OF DISEASES:** DSM-IV.

**DIAGNOSTIC CATEGORIES:** non-affective psychoses.

**STUDY AREA:** USA.  **POPULATION SIZE (denominator):** 9,282.

**AGE RANGE: LOWER:** 18. **UPPER:** Unlimited.

**STUDY QUALITY:** 13.

**===============================================================================================**

**NUMBER OF ESTIMATES:** 46 (12-month) + 29 (lifetime).

**PREVALENCE RATE (indicated period: point, 12-month, lifetime)**

**PREVALENCE RATE (12-month):** 5.00 per 1,000 inhabitants. **STANDARD ERROR:** 1.00 **CI: LOW:**  **UPPER:**

**PREVALENCE RATE (Lifetime):** 3.12 per 1,000 inhabitants. **STANDARD ERROR:** 1.50 **CI: LOW:**  **UPPER:**

**MEN:** **STANDARD ERROR: CI: LOW:**  **UPPER:**

**WOMEN:** **STANDARD ERROR: CI: LOW:**  **UPPER:**

**===============================================================================================**

**OBSERVATIONS:**

**REFERENCE NUMBER:** 49

**AUTHORS:** Kessler RC, McGonagle KA, Zhao S, Nelson CB, Hughes M, Eshleman S, Wittchen H and Kendler K.

**YEAR OF PUBLICATION:** 1994.

**TITLE:** Lifetime and 12-Month Prevalence of DSM-III-R Psychiatric Disorders in the United States: Results From the National Comorbidity Survey.

**JOURNAL:** *Archives of General Psychiatry, 51*(1):8-19.

**===============================================================================================**

**STUDY COUNTRY:** USA. **STUDY YEAR:** 1992.

**CASE FINDING SETTING:** census, general population.

**SAMPLING:** Nonrandom.

**If sampling, indicate Clusters: CL1-CL2-P/CL:**

**NUMBER OF STAGES:** 2.

**SCREENING INSTRUMENT:**

**RESPONSE % PHASE 1:**   **RESPONSE % PHASE 2:**

**DIAGNOSTIC INSTRUMENT:** CIDI (Composite International Diagnostic Interview).

**CLASSIFICATION OF DISEASES:** DSM-III-R.

**DIAGNOSTIC CATEGORIES:** non-affective psychoses.

**STUDY AREA:** USA.  **POPULATION SIZE (denominator):** 8,098.

**AGE RANGE: LOWER:** 15. **UPPER:** 54.

**STUDY QUALITY:** 15.

**===============================================================================================**

**NUMBER OF ESTIMATES:** 40 (12-month) + 57 (lifetime).

**PREVALENCE RATE (indicated period: point, 12-month, lifetime)**

**PREVALENCE RATE (12-month):** 4.94 per 1,000 inhabitants. **STANDARD ERROR:** 1.00 **CI: LOW:**  **UPPER:**

**PREVALENCE RATE (Lifetime):** 7.04 per 1,000 inhabitants. **STANDARD ERROR:** 1.00 **CI: LOW:**  **UPPER:**

**MEN:** **STANDARD ERROR: CI: LOW:**  **UPPER:**

**WOMEN:** **STANDARD ERROR: CI: LOW:**  **UPPER:**

**===============================================================================================**

**OBSERVATIONS:**

**REFERENCE NUMBER:** 50

**AUTHORS:** Kodesh A, Goldshtein I, Gelkopf M, Goren I, Chodick G and Shalev V.

**YEAR OF PUBLICATION:** 2012.

**TITLE:** Epidemiology and comorbidity of severe mental illnesses in the community: findings from a computerized mental health registry in a large Israeli health organization.

**JOURNAL:** *Social Psychiatry and Psychiatric Epidemiology, 47*(11), 1775-1782.

**===============================================================================================**

**STUDY COUNTRY:** Israel. **STUDY YEAR:** 2009.

**CASE FINDING SETTING:** attended population in Mental Health Services + Primary Care.

**SAMPLING:** Nonrandom.

**If sampling, indicate Clusters: CL1-CL2-P/CL:**

**NUMBER OF STAGES:** 1.

**SCREENING INSTRUMENT:**

**RESPONSE % PHASE 1: RESPONSE % PHASE 2:**

**DIAGNOSTIC INSTRUMENT:** clinical.

**CLASSIFICATION OF DISEASES:** ICD-9.

**DIAGNOSTIC CATEGORIES:** Schizophrenia and related disorders

**STUDY AREA:** Israel. **POPULATION SIZE (denominator):** 8,848.

**AGE RANGE: LOWER:** 5. **UPPER:** 85.

**STUDY QUALITY:** 8.

**===============================================================================================**

**NUMBER OF ESTIMATES:** 44.

**PREVALENCE RATE (indicated period: point, 12-month, lifetime)**

**PREVALENCE RATE (12-month):** 4.97 per 1,000 inhabitants. **STANDARD ERROR:**   **CI: LOW:**  **UPPER:**

**MEN:** 5.40 per 1,000 inhabitants. **STANDARD ERROR: CI: LOW:**  **UPPER:**

**WOMEN:** 5.10 per 1,000 inhabitants. **STANDARD ERROR: CI: LOW:**  **UPPER:**

**===============================================================================================**

**OBSERVATIONS:**

**REFERENCE NUMBER:** 51

**AUTHORS:** Kringlen E, Torgersen S and Cramer V.

**YEAR OF PUBLICATION:** 2001.

**TITLE:** A Norwegian Psychiatric Epidemiological Study.

**JOURNAL:** *American Journal of Psychiatry, 158*(7), 1091-1098.

**===============================================================================================**

**STUDY COUNTRY:** Norway.  **STUDY YEAR:** 1997.

**CASE FINDING SETTING:** census, general population.

**SAMPLING:** random stratified.

**If sampling, indicate Clusters: CL1-CL2-P/CL:**

**NUMBER OF STAGES:** 1.

**SCREENING INSTRUMENT:**

**RESPONSE % PHASE 1: RESPONSE % PHASE 2:**

**DIAGNOSTIC INSTRUMENT:** CIDI (Composite International Diagnostic Interview).

**CLASSIFICATION OF DISEASES:** DSM-III-R.

**DIAGNOSTIC CATEGORIES:** non-affective psychoses.

**STUDY AREA:** Norway.  **POPULATION SIZE (denominator):** 2,066.

**AGE RANGE: LOWER:** 18. **UPPER:** 65.

**STUDY QUALITY:** 12.

**===============================================================================================**

**NUMBER OF ESTIMATES:** 4 (12-month) + 8 (lifetime).

**PREVALENCE RATE (indicated period: point, 12-month, lifetime)**

**PREVALENCE RATE (12-month):** 1.94 per 1,000 inhabitants. **STANDARD ERROR:** 1.00  **CI: LOW:**  **UPPER:**

**PREVALENCE RATE (Lifetime):** 3.87 per 1,000 inhabitants. **STANDARD ERROR:** 2.00  **CI: LOW:**  **UPPER:**

**MEN:** **STANDARD ERROR: CI: LOW:**  **UPPER:**

**WOMEN:** **STANDARD ERROR: CI: LOW:**  **UPPER:**

**===============================================================================================**

**OBSERVATIONS:**

**REFERENCE NUMBER:** 52

**AUTHORS:** Kringlen E, Torgersen S and Cramer V.

**YEAR OF PUBLICATION:** 2006.

**TITLE:** Mental illness in a rural area: a Norwegian psychiatric epidemiological study.

**JOURNAL:** *Social Psychiatry and Psychiatric Epidemiology, 41*(9), 713-719.

**===============================================================================================**

**STUDY COUNTRY:** Norway. **STUDY YEAR:** 1999.

**CASE FINDING SETTING:** census, general population.

**SAMPLING:** random stratified.

**If sampling, indicate Clusters: CL1-CL2-P/CL:**

**NUMBER OF STAGES:** 1.

**SCREENING INSTRUMENT:**

**RESPONSE % PHASE 1: RESPONSE % PHASE 2:**

**DIAGNOSTIC INSTRUMENT:** CIDI (Composite International Diagnostic Interview).

**CLASSIFICATION OF DISEASES:** DSM-III-R.

**DIAGNOSTIC CATEGORIES:** non-affective psychoses.

**STUDY AREA:** Sogn and Fjordane.  **POPULATION SIZE (denominator):** 1,080.

**AGE RANGE: LOWER:** 18. **UPPER:** 65.

**STUDY QUALITY:** 15.

**===============================================================================================**

**NUMBER OF ESTIMATES:** 3 (12-month) + 4 (lifetime).

**PREVALENCE RATE (indicated period: point, 12-month, lifetime)**

**PREVALENCE RATE (12-month):** 2.78 per 1,000 inhabitants. **STANDARD ERROR:** 2.00  **CI: LOW:**  **UPPER:**

**PREVALENCE RATE (Lifetime):** 3.70 per 1,000 inhabitants. **STANDARD ERROR:** 2.00  **CI: LOW:**  **UPPER:**

**MEN:** 2.00 (12-month) / 2.00 (lifetime) per 1,000 inhabitants. **STANDARD ERROR: CI: LOW:**  **UPPER:**

**WOMEN:** 4.00 (12-month) / 6.00 (lifetime) per 1,000 inhabitants. **STANDARD ERROR: CI: LOW:**  **UPPER:**

**===============================================================================================**

**OBSERVATIONS:**

**REFERENCE NUMBER:** 53

**AUTHORS:** Kurihara T, Kato M, Reverger R, Tirta IGR and Kashima H.

**YEAR OF PUBLICATION:** 2005.

**TITLE:** Never-treated patients with Schizophrenia in the developing country of Bali.

**JOURNAL:** *Schizophrenia Research, 79*(2-3), 307 –13.

**===============================================================================================**

**STUDY COUNTRY:** Indonesia. **STUDY YEAR:** 2002.

**CASE FINDING SETTING:** census, general population.

**SAMPLING:** Simple random.

**If sampling, indicate Clusters: CL1-CL2-P/CL:**

**NUMBER OF STAGES:** 1.

**SCREENING INSTRUMENT:**

**RESPONSE % PHASE 1: RESPONSE % PHASE 2:**

**DIAGNOSTIC INSTRUMENT:** SCID (Structure Clinical Interview for DSM-IV) + clinical.

**CLASSIFICATION OF DISEASES:** DSM-III-R.

**DIAGNOSTIC CATEGORIES:** schizophrenia.

**STUDY AREA:** Bali. **POPULATION SIZE (denominator):** 8,546.

**AGE RANGE: LOWER:** 15. **UPPER:** Unlimited.

**STUDY QUALITY:** 11.

**===============================================================================================**

**NUMBER OF ESTIMATES:** 36.

**PREVALENCE RATE (indicated period: point, 12-month, lifetime)**

**PREVALENCE RATE (Point):** 4.21 per 1,000 inhabitants. **STANDARD ERROR:**  **CI: LOW:**  **UPPER:**

**MEN:** **STANDARD ERROR: CI: LOW:**  **UPPER:**

**WOMEN:** **STANDARD ERROR: CI: LOW:**  **UPPER:**

**===============================================================================================**

**OBSERVATIONS:**

**REFERENCE NUMBER:** 54

**AUTHORS:** Lindström E, Widerlöv B and von Knorring L.

**YEAR OF PUBLICATION:** 1997.

**TITLE:** The ICD-10 and DSM-IV diagnostic criteria and the prevalence of schizophrenia.

**JOURNAL:** *European Psychiatry, 12,* 217-223.

**===============================================================================================**

**STUDY COUNTRY:** Sweden. **STUDY YEAR:** 1991.

**CASE FINDING SETTING:** attended population in Mental Health Services + Primary Care.

**SAMPLING:** nonrandom.

**If sampling, indicate Clusters: CL1-CL2-P/CL:**

**NUMBER OF STAGES:** 1.

**SCREENING INSTRUMENT:**

**RESPONSE % PHASE 1: RESPONSE % PHASE 2:**

**DIAGNOSTIC INSTRUMENT:** clinical.

**CLASSIFICATION OF DISEASES:** DSM-III-R.

**DIAGNOSTIC CATEGORIES:** schizophrenia.

**STUDY AREA:** Uppsala. **POPULATION SIZE (denominator):** 64,886.

**AGE RANGE: LOWER:** 18. **UPPER:** Unlimited.

**STUDY QUALITY:** 9.

**===============================================================================================**

**NUMBER OF ESTIMATES:** 273.

**PREVALENCE RATE (indicated period: point, 12-month, lifetime)**

**PREVALENCE RATE (12-month):** 4.21 per 1,000 inhabitants. **STANDARD ERROR:**  **CI: LOW:**  **UPPER:**

**MEN:** 5.00 per 1,000 inhabitants. **STANDARD ERROR: CI: LOW:**  **UPPER:**

**WOMEN:** 3.50 per 1,000 inhabitants. **STANDARD ERROR: CI: LOW:**  **UPPER:**

**===============================================================================================**

**OBSERVATIONS:**

**REFERENCE NUMBER:** 55

**AUTHORS:** McConnell P, Bebbington P, McClelland R, Gillespie K and Houghton S.

**YEAR OF PUBLICATION:** 2002.

**TITLE:** Prevalence of psychiatric disorder and the need for psychiatric care in Northern Ireland: Population study in the District of Derry.

**JOURNAL:** *The* *British Journal of Psychiatry, 181,* 214-219.

**===============================================================================================**

**STUDY COUNTRY:** Ireland. **STUDY YEAR:** 1994.

**CASE FINDING SETTING:** census, general population.

**SAMPLING:** random stratified.

**If sampling, indicate Clusters: CL1-CL2-P/CL:**

**NUMBER OF STAGES:** 2.

**SCREENING INSTRUMENT:**

**RESPONSE % PHASE 1: RESPONSE % PHASE 2:**

**DIAGNOSTIC INSTRUMENT:** SCAN (Schedules for Clinical Assessment in Neuropsychiatry).

**CLASSIFICATION OF DISEASES:** ICD-10.

**DIAGNOSTIC CATEGORIES:** schizophrenia.

**STUDY AREA:** Ireland.  **POPULATION SIZE (denominator):** 1,242.

**AGE RANGE: LOWER:** 18. **UPPER:** 65.

**STUDY QUALITY:** 10.

**===============================================================================================**

**NUMBER OF ESTIMATES:** 5.

**PREVALENCE RATE (indicated period: point, 12-month, lifetime)**

**PREVALENCE RATE (12-month):** 4.03 per 1,000 inhabitants. **STANDARD ERROR:**  **CI: LOW:** 0.00 **UPPER:** 10.00

**MEN:**  **STANDARD ERROR: CI: LOW:**  **UPPER:**

**WOMEN:** **STANDARD ERROR: CI: LOW:**  **UPPER:**

**===============================================================================================**

**OBSERVATIONS:**

**REFERENCE NUMBER:** 56

**AUTHORS:** McCreadie RG, Leese M, Tilak-Singh D, Loftus L, MacEwan T and Thornicroft G.

**YEAR OF PUBLICATION:** 1997.

**TITLE:** Nithsdale, Nunhead and Norwood: Similarities and Differences in Prevalence of Schizophrenia and Utilisation of Services in Rural and Urban Areas.

**JOURNAL:** *The British Journal of Psychiatry, 170*(1), 31-36.

**===============================================================================================**

**STUDY COUNTRY:** United Kingdom. **STUDY YEAR:** 1992.

**CASE FINDING SETTING:** attended population in Mental Health Services + Primary Care + Social Services.

**SAMPLING:** simple random.

**If sampling, indicate Clusters: CL1-CL2-P/CL:**

**NUMBER OF STAGES:** 1.

**SCREENING INSTRUMENT:**

**RESPONSE % PHASE 1: RESPONSE % PHASE 2:**

**DIAGNOSTIC INSTRUMENT:** OPCRIT (Operational Criteria Checklist for Psychosis).

**CLASSIFICATION OF DISEASES:** ICD-10.

**DIAGNOSTIC CATEGORIES:** schizophrenia.

**STUDY AREA:** United Kingdom.

**POPULATION SIZE (denominator):** 57,831 (Nishsdale), 29,448 (Nunhead) and 23,007 (Norwood).

**AGE RANGE: LOWER:** 18. **UPPER:** 90.

**STUDY QUALITY:** 12.

**===============================================================================================**

**NUMBER OF ESTIMATES:** 161 in Nithsdale, 102 in Nunhead and 52 in Norwood.

**PREVALENCE RATE (indicated period: point, 12-month, lifetime)**

**PREVALENCE RATE (Point):** 2.78 (Nithsdale) / 3.46 (Nunhead) / 2.24 (Norwood) per 1,000 inhabitants.

**STANDARD ERROR:** 1.00 **CI: LOW:**  **UPPER:**

**MEN:**  **STANDARD ERROR: CI: LOW:**  **UPPER:**

**WOMEN:** **STANDARD ERROR: CI: LOW:**  **UPPER:**

**===============================================================================================**

**OBSERVATIONS:**

**REFERENCE NUMBER:** 57

**AUTHORS:** Moreno B, García-Alonso CR, Negin Hernández MA, Torres-González F and Salvador-Carulla L.

**YEAR OF PUBLICATION:** 2008.

**TITLE:** Spatial analysis to identify hotspots of prevalence of schizophrenia.

**JOURNAL:** *Social Psychiatry and Psychiatric Epidemiology, 43*(10), 782–91.

**===============================================================================================**

**STUDY COUNTRY:** Spain. **STUDY YEAR:** 1999.

**CASE FINDING SETTING:** attended population in Mental Health Services + Primary Care.

**SAMPLING:** nonrandom.

**If sampling, indicate Clusters: CL1-CL2-P/CL:**

**NUMBER OF STAGES:** 1.

**SCREENING INSTRUMENT:**

**RESPONSE % PHASE 1: RESPONSE % PHASE 2:**

**DIAGNOSTIC INSTRUMENT:** clinical.

**CLASSIFICATION OF DISEASES:** ICD-10.

**DIAGNOSTIC CATEGORIES:** Schizophrenia and related disorders

**STUDY AREA:** Granada.  **POPULATION SIZE (denominator):** 270,629.

**AGE RANGE: LOWER:** 14. **UPPER:** Unlimited.

**STUDY QUALITY:** 9.

**===============================================================================================**

**NUMBER OF ESTIMATES:** 774.

**PREVALENCE RATE (indicated period: point, 12-month, lifetime)**

**PREVALENCE RATE (12-month):** 2.86 per 1,000 inhabitants. **STANDARD ERROR:**  **CI: LOW:**  **UPPER:**

**MEN:**  **STANDARD ERROR: CI: LOW:**  **UPPER:**

**WOMEN:** **STANDARD ERROR: CI: LOW:**  **UPPER:**

**===============================================================================================**

**OBSERVATIONS:**

**REFERENCE NUMBER:** 58

**AUTHORS:** Moreno­Küstner B, Mayoral F, Navas­Campaña D, García­Herrera JM, Angona P, Martín C and Rivas F.

**YEAR OF PUBLICATION:** 2016

**TITLE:** Prevalence of schizophrenia and related disorders in Malaga (Spain): results using multiple clinical databases.

**JOURNAL:** *Epidemiology and Psychiatric Sciences, 25,* 38-58.

**===============================================================================================**

**STUDY COUNTRY:** Spain. **STUDY YEAR:** 2008.

**CASE FINDING SETTING:** attended population in Mental Health Services + Primary Care.

**SAMPLING:** nonrandom.

**If sampling, indicate Clusters: CL1-CL2-P/CL:**

**NUMBER OF STAGES:** 1.

**SCREENING INSTRUMENT:**

**RESPONSE % PHASE 1: RESPONSE % PHASE 2:**

**DIAGNOSTIC INSTRUMENT:** clinical.

**CLASSIFICATION OF DISEASES:** ICD-10.

**DIAGNOSTIC CATEGORIES:** Schizophrenia and related disorders

**STUDY AREA:** Malaga.  **POPULATION SIZE (denominator):** 265,229.

**AGE RANGE: LOWER:** 14. **UPPER:** Unlimited.

**STUDY QUALITY:** 11.

**===============================================================================================**

**NUMBER OF ESTIMATES:** 1053.

**PREVALENCE RATE (indicated period: point, 12-month, lifetime)**

**PREVALENCE RATE (12-month):** 3.97 per 1,000 inhabitants. **STANDARD ERROR:** 0.12 **CI: LOW:** 1.95 **UPPER:** 6.31

**MEN:** 5.88 per 1,000 inhabitants. **STANDARD ERROR:** 0.21 **CI: LOW:** 5.46 **UPPER:** 6.31

**WOMEN:** 2.20 per 1,000 inhabitants. **STANDARD ERROR:** 0.13 **CI: LOW:** 1.95 **UPPER:** 2.24

**===============================================================================================**

**OBSERVATIONS:**

**REFERENCE NUMBER:** 59

**AUTHORS:** Morgan VA, McGrath JJ, Jablensky A, Badcock JC, Waterreus A, Bush R, Carr V, Castle D, Cohen M, Galletly C, Harvey C, Hocking B, McGorry P, Neil AL, Saw S, Shah S, Stain HJ and Mackinnon A.

**YEAR OF PUBLICATION:** 2014.

**TITLE:** Psychosis prevalence and Physical, metabolic and cognitive co-morbidity: data from the second Australian National survey of psychosis.

**JOURNAL:** *Psychological Medicine, 44,* 2163-2176.

**===============================================================================================**

**STUDY COUNTRY:** Australia. **STUDY YEAR:** 2010.

**CASE FINDING SETTING:** census, general population.

**SAMPLING:** Stratified random sampling.

**If sampling, indicate Clusters: CL1-CL2-P/CL:**

**NUMBER OF STAGES:** 2.

**SCREENING INSTRUMENT:** OPCRIT (Operational Criteria Checklist for Psychosis).

**RESPONSE % PHASE 1: RESPONSE % PHASE 2:**

**DIAGNOSTIC INSTRUMENT:** DIP (Diagnostic Interview for Psychoses).

**CLASSIFICATION OF DISEASES:** ICD-10.

**DIAGNOSTIC CATEGORIES:** NAP (non-affective psychoses).

**STUDY AREA:** 5 States of Australia. **POPULATION SIZE (denominator):** 4,928.

**AGE RANGE: LOWER:** 18. **UPPER:** 64.

**STUDY QUALITY:** 13.

**===============================================================================================**

**NUMBER OF ESTIMATES:** 10 (point) + 12 (lifetime).

**PREVALENCE RATE (indicated period: point, 12-month, lifetime)**

**PREVALENCE RATE (Point):** 3.10 per 1,000 inhabitants. **STANDARD ERROR:**  **CI: LOW:** 1.99  **UPPER:** 2.25

**PREVALENCE RATE (Lifetime):** 3.45 per 1,000 inhabitants. **STANDARD ERROR:**  **CI: LOW:** 2.33 **UPPER:** 2.52

**MEN:** point 2.89 / lifetime 3.31. **STANDARD ERROR: CI: LOW:** 2.68/3.07 **UPPER:** 3.10/3.55

**WOMEN:** point 1.35 / lifetime 1.48. **STANDARD ERROR: CI: LOW:** 1.13/1.23 **UPPER:** 1.57/1.72

**===============================================================================================**

**OBSERVATIONS:**

**REFERENCE NUMBER:** 60

**AUTHORS:** Myles-Worsley M, Coon H, Tiobech J, Collier J, Dale P, Wender P, Reimherr F, Polloi A and Byerley W.

**YEAR OF PUBLICATION:** 1999.

**TITLE:** Genetic Epidemiological Study of Schizophrenia in Palau, Micronesia: Prevalence and Familiarity.

**JOURNAL:** *American Journal of Medical Genetics, 88*(1), 4-10.

**===============================================================================================**

**STUDY COUNTRY:** Micronesia. **STUDY YEAR:** 1996.

**CASE FINDING SETTING:** attended population in Mental Health Services.

**SAMPLING:** simple random.

**If sampling, indicate Clusters: CL1-CL2-P/CL:**

**NUMBER OF STAGES:** 1.

**SCREENING INSTRUMENT:**

**RESPONSE % PHASE 1: RESPONSE % PHASE 2:**

**DIAGNOSTIC INSTRUMENT:** SADS (Schedule for Affective Disorders and Schizophrenia).

**CLASSIFICATION OF DISEASES:** DSM-III-R.

**DIAGNOSTIC CATEGORIES:** Schizophrenia and related disorders .

**STUDY AREA:** Micronesia.  **POPULATION SIZE (denominator):** 13,750.

**AGE RANGE: LOWER:** 15. **UPPER:** 90.

**STUDY QUALITY:** 10.

**===============================================================================================**

**NUMBER OF ESTIMATES:** 262.

**PREVALENCE RATE (indicated period: point, 12-month, lifetime)**

**PREVALENCE RATE (Lifetime):** 19.90 per 1,000 inhabitants. **STANDARD ERROR:**  **CI: LOW:**  **UPPER:**

**MEN:** 27.70 per 1,000 inhabitants. **STANDARD ERROR: CI: LOW: UPPER:**

**WOMEN:** 12.40 per 1,000 inhabitants. **STANDARD ERROR: CI: LOW: UPPER:**

**===============================================================================================**

**OBSERVATIONS:**

**REFERENCE NUMBER:** 61

**AUTHORS:** Nimgaonkar VL, Gentry K, Maendel S, Maendel M and Eaton J.

**YEAR OF PUBLICATION:** 2000.

**TITLE:** Low Prevalence of Psychoses Among the Hutterites, an Isolated Religious Community.

**JOURNAL:** *American Journal of Psychitry, 157*, 1065-1070.

**===============================================================================================**

**STUDY COUNTRY:** Canada. **STUDY YEAR:** 1997.

**CASE FINDING SETTING:** attended population in Mental Health Services + Primary Care.

**SAMPLING:** nonrandom.

**If sampling, indicate Clusters: CL1-CL2-P/CL:**

**NUMBER OF STAGES:** 1.

**SCREENING INSTRUMENT:**

**RESPONSE % PHASE 1: RESPONSE % PHASE 2:**

**DIAGNOSTIC INSTRUMENT:** clinical.

**CLASSIFICATION OF DISEASES:** DSM-IV.

**DIAGNOSTIC CATEGORIES:** schizophrenia.

**STUDY AREA:** Manitoba. **POPULATION SIZE (denominator):** 8,542.

**AGE RANGE: LOWER:** 15. **UPPER:** Unlimited.

**STUDY QUALITY:** 9.

**===============================================================================================**

**NUMBER OF ESTIMATES:** 11.

**PREVALENCE RATE (indicated period: point, 12-month, lifetime)**

**PREVALENCE RATE (12-month):** 1.29 per 1,000 inhabitants. **STANDARD ERROR:**  **CI: LOW:**  **UPPER:**

**MEN:** **STANDARD ERROR: CI: LOW: UPPER:**

**WOMEN:** **STANDARD ERROR: CI: LOW: UPPER:**

**===============================================================================================**

**OBSERVATIONS:**

**REFERENCE NUMBER:** 62

**AUTHORS:** Ortega MA, Seva A and Pérez A.

**YEAR OF PUBLICATION:** 1995.

**TITLE:** Morbilidad psíquica diagnóstica en la población general de La Rioja.

**JOURNAL:** *Anales de Psiquiatría, 2*(9), 320-326.

**===============================================================================================**

**STUDY COUNTRY:** Spain. **STUDY YEAR:** 1992.

**CASE FINDING SETTING:** census, general population.

**SAMPLING:** cluster.

**If sampling, indicate Clusters: CL1-CL2-P/CL:** CL2

**NUMBER OF STAGES:** 2.

**SCREENING INSTRUMENT:**

**RESPONSE % PHASE 1: RESPONSE % PHASE 2:**

**DIAGNOSTIC INSTRUMENT:** CIS (Clinical Interview Shedule).

**CLASSIFICATION OF DISEASES:** DSM-III-R.

**DIAGNOSTIC CATEGORIES:** Schizophrenia and related disorders .

**STUDY AREA:** The Rioja.  **POPULATION SIZE (denominator):** 793.

**AGE RANGE: LOWER:** 15. **UPPER:** 65.

**STUDY QUALITY:** 14.

**===============================================================================================**

**NUMBER OF ESTIMATES:** 1.

**PREVALENCE RATE (indicated period: point, 12-month, lifetime)**

**PREVALENCE RATE (Point):** 1.26 per 1,000 inhabitants. **STANDARD ERROR:**  **CI: LOW:**  **UPPER:**

**MEN:** **STANDARD ERROR: CI: LOW: UPPER:**

**WOMEN:** **STANDARD ERROR: CI: LOW: UPPER:**

**===============================================================================================**

**OBSERVATIONS:**

**REFERENCE NUMBER:** 8

**AUTHORS:** Perälä J, Suvisaari J, Saarni SI, Kuoppasalmi K, Isometsä E, Pirkola S, Partonen T, Tuulio-Henriksson A, Hintikka J, Kieseppä T, Härkänen T, Koskinen S and Lönnqvist J.

**YEAR OF PUBLICATION:** 2007.

**TITLE:** Lifetime Prevalence of Psychotic and Bipolar I Disorders in a General Population.

**JOURNAL:** *Archives General of Psychiatry, 64*(1):19-28.

**===============================================================================================**

**STUDY COUNTRY:** Finland. **STUDY YEAR:** 2004.

**CASE FINDING SETTING:** census, general population.

**SAMPLING:** nonrandom.

**If sampling, indicate Clusters: CL1-CL2-P/CL:**

**NUMBER OF STAGES:** 2.

**SCREENING INSTRUMENT:**

**RESPONSE % PHASE 1: RESPONSE % PHASE 2:**

**DIAGNOSTIC INSTRUMENT:** SCID (Structure Clinical Interview for DSM-IV).

**CLASSIFICATION OF DISEASES:** DSM-IV.

**DIAGNOSTIC CATEGORIES:** schizophrenia.

**STUDY AREA:** Finland.  **POPULATION SIZE (denominator):** 8,028.

**AGE RANGE: LOWER:** 30. **UPPER:** 90.

**STUDY QUALITY:** 16.

**===============================================================================================**

**NUMBER OF ESTIMATES:** 70.

**PREVALENCE RATE (indicated period: point, 12-month, lifetime)**

**PREVALENCE RATE (Lifetime):** 8.72 per 1,000 inhabitants. **STANDARD ERROR:**  **CI: LOW:** 6.80 **UPPER:** 11.10

**MEN:** 8.20 per 1,000 inhabitants. **STANDARD ERROR: CI: LOW: UPPER:**

**WOMEN:** 9.10 per 1,000 inhabitants. **STANDARD ERROR: CI: LOW: UPPER:**

**===============================================================================================**

**OBSERVATIONS:**

**REFERENCE NUMBER:** 63

**AUTHORS:** Perälä J, Saarni SI, Ostamo A, Pirkola S, Haukka J, Härkänen T, Koskinen S, Lönnqvist J and Suvisaari J.

**YEAR OF PUBLICATION:** 2008.

**TITLE:** Geographic variation and sociodemographic characteristics of psychotic disorders in Finland.

**JOURNAL:** *Schizophrenia Research, 106*(2-3), 337-347.

**===============================================================================================**

**STUDY COUNTRY:** Finland. **STUDY YEAR:** 2004.

**CASE FINDING SETTING:** census, general population.

**SAMPLING:** clusters.

**If sampling, indicate Clusters: CL1-CL2-P/CL:** CL2

**NUMBER OF STAGES:** 2.

**SCREENING INSTRUMENT:** CIDI (Composite International Diagnostic Interview).

**RESPONSE % PHASE 1: RESPONSE % PHASE 2:**

**DIAGNOSTIC INSTRUMENT:** SCID-I (Structure Clinical Interview for DSM-IV).

**CLASSIFICATION OF DISEASES:** DSM-IV + DSM-IV-TR.

**DIAGNOSTIC CATEGORIES:** schizophrenia.

**STUDY AREA:** 80 areas of Finland. **POPULATION SIZE (denominator):** Sample = 8,028.

**AGE RANGE: LOWER:** 30. **UPPER:** 65.

**STUDY QUALITY:** 10.

**===============================================================================================**

**NUMBER OF ESTIMATES:** 51 (Southwest) + 74 (South) + 63 (West) + 86 (East) + 148 (North).

**PREVALENCE RATE (indicated period: point, 12-month, lifetime)**

**PREVALENCE RATE (Lifetime):** 6.35 Southwest/9.22 South/7.85 West/10.71 East/18.44 North 🡪 **6.30-18.40** per 1,000 inhabitants.

**STANDARD ERROR:** **CI: LOW: 3.20**/ 6.10/ 4.20/ 5.70/ 12.50 **UPPER:** 12.20/ 13.70/ 14.20/ 20.00/ **27.10**

**MEN:** **STANDARD ERROR: CI: LOW: UPPER:**

**WOMEN:** **STANDARD ERROR: CI: LOW: UPPER:**

**===============================================================================================**

**OBSERVATIONS:**

**REFERENCE NUMBER:** 64

**AUTHORS:** Phanthunane P, Vos T, Whiteford H, Bertram M and Udomratn P.

**YEAR OF PUBLICATION:** 2010.

**TITLE:** Schizophrenia in Thailand: prevalence and burden of disease.

**JOURNAL:** *Population Health Metrics, 8,* 24.

**===============================================================================================**

**STUDY COUNTRY:** Thailand. **STUDY YEAR:** 2003.

**CASE FINDING SETTING:** census, general population.

**SAMPLING:** random stratified.

**If sampling, indicate Clusters: CL1-CL2-P/CL:**

**NUMBER OF STAGES:** 2.

**SCREENING INSTRUMENT:** Screening questionnaire for community mental health.

**RESPONSE % PHASE 1: RESPONSE % PHASE 2:**

**DIAGNOSTIC INSTRUMENT:** MINI (Mini-International Neuropsychiatric Interview).

**CLASSIFICATION OF DISEASES:** DSM-IV + ICD-10.

**DIAGNOSTIC CATEGORIES:** Schizophrenia and related disorders .

**STUDY AREA:** Thailand.  **POPULATION SIZE (denominator):** 11,700.

**AGE RANGE: LOWER:** 15. **UPPER:** 59.

**STUDY QUALITY:** 12.

**===============================================================================================**

**NUMBER OF ESTIMATES:** 103.

**PREVALENCE RATE (indicated period: point, 12-month, lifetime)**

**PREVALENCE RATE (Lifetime):** 8.80 per 1,000 inhabitants. **STANDARD ERROR:** **CI: LOW:** 7.20 **UPPER:** 10.60

**MEN:** 8.10 per 1,000 inhabitants. **STANDARD ERROR: CI: LOW: UPPER:**

**WOMEN:** 7.30 per 1,000 inhabitants. **STANDARD ERROR: CI: LOW: UPPER:**

**===============================================================================================**

**OBSERVATIONS:**

**REFERENCE NUMBER:** 65

**AUTHORS:** Phillips MR, Yang G, Li S and Li Y.

**YEAR OF PUBLICATION:** 2004.

**TITLE:** Suicide and the unique prevalence pattern of schizophrenia in mainland China: a retrospective observational study.

**JOURNAL:** *The* *Lancet, 364*(9439), 1062–1068.

**===============================================================================================**

**STUDY COUNTRY:** China. **STUDY YEAR:** 1999.

**CASE FINDING SETTING:** census, general population.

**SAMPLING:** random stratified.

**If sampling, indicate Clusters: CL1-CL2-P/CL:**

**NUMBER OF STAGES:** 3.

**SCREENING INSTRUMENT:** Screening questionnaire for community mental health.

**RESPONSE % PHASE 1:** 90% **RESPONSE % PHASE 2:**

**DIAGNOSTIC INSTRUMENT:** clinical.

**CLASSIFICATION OF DISEASES:** ICD-9.

**DIAGNOSTIC CATEGORIES:** schizophrenia.

**STUDY AREA:** Mainland. **POPULATION SIZE (denominator):** 19,223.

**AGE RANGE: LOWER:** 15. **UPPER:** Unlimited.

**STUDY QUALITY:** 11.

**===============================================================================================**

**NUMBER OF ESTIMATES:** 90.

**PREVALENCE RATE (indicated period: point, 12-month, lifetime)**

**PREVALENCE RATE (Point):** 4.68 per 1,000 inhabitants. **STANDARD ERROR:** **CI: LOW:** 1.50 **UPPER:** 5.80

**MEN:** **STANDARD ERROR: CI: LOW: UPPER:**

**WOMEN:** 1.77 per 1,000 inhabitants. **STANDARD ERROR: CI: LOW:** 1.15 **UPPER:** 2.72

**===============================================================================================**

**OBSERVATIONS:**

**REFERENCE NUMBER:** 66

**AUTHORS:** Phillips MR, Zhang J, Shi Q, Song Z, Ding Z, Pang S, Li X, Zhang Y and Wang Z.

**YEAR OF PUBLICATION:** 2009.

**TITLE:** Prevalence, treatment, and associated disability of mental disorders in four provinces in China during 2001–05: an epidemiological survey.

**JOURNAL:** *The Lancet, 373*(9680), 2041-2053.

**===============================================================================================**

**STUDY COUNTRY:** China. **STUDY YEAR:** 2005.

**CASE FINDING SETTING:** census, general population.

**SAMPLING:** nonrandom.

**If sampling, indicate Clusters: CL1-CL2-P/CL:**

**NUMBER OF STAGES:** 2.

**SCREENING INSTRUMENT:**

**RESPONSE % PHASE 1: RESPONSE % PHASE 2:**

**DIAGNOSTIC INSTRUMENT:** SCID (Structure Clinical Interview for DSM-IV).

**CLASSIFICATION OF DISEASES:** DSM-IV.

**DIAGNOSTIC CATEGORIES:** schizophrenia.

**STUDY AREA:** China.  **POPULATION SIZE (denominator):** 63,004.

**AGE RANGE: LOWER:** 18. **UPPER:** 90.

**STUDY QUALITY:** 16.

**===============================================================================================**

**NUMBER OF ESTIMATES:** 492.

**PREVALENCE RATE (indicated period: point, 12-month, lifetime)**

**PREVALENCE RATE (Point):** 7.81 per 1,000 inhabitants. **STANDARD ERROR:** **CI: LOW:** 6.30 **UPPER:** 9.50

**MEN:** 8.20 per 1,000 inhabitants. **STANDARD ERROR: CI: LOW: UPPER:**

**WOMEN:** 7.40 per 1,000 inhabitants. **STANDARD ERROR: CI: LOW:** 1.15 **UPPER:** 2.72

**===============================================================================================**

**OBSERVATIONS:**

**REFERENCE NUMBER:** 67

**AUTHORS:** Pringle DG, Waddington JL and Youssef HA.

**YEAR OF PUBLICATION:** 1995.

**TITLE:** Schizophrenia in East County Cavan: Spatial Variations in Prevalence and their Aetiological Implications.

**JOURNAL:** *Irish Geography, 28*(1), 1­13.

**===============================================================================================**

**STUDY COUNTRY:** Ireland. **STUDY YEAR:** 1987.

**CASE FINDING SETTING:** attended population in Mental Health Services + Primary Care.

**SAMPLING:** nonrandom.

**If sampling, indicate Clusters: CL1-CL2-P/CL:**

**NUMBER OF STAGES:** 1.

**SCREENING INSTRUMENT:**

**RESPONSE % PHASE 1: RESPONSE % PHASE 2:**

**DIAGNOSTIC INSTRUMENT:** clinical.

**CLASSIFICATION OF DISEASES:** DSM-III-R.

**DIAGNOSTIC CATEGORIES:** schizophrenia.

**STUDY AREA:** County Cavan. **POPULATION SIZE (denominator):** 37,272.

**AGE RANGE: LOWER:** 21. **UPPER:** 85.

**STUDY QUALITY:** 11.

**===============================================================================================**

**NUMBER OF ESTIMATES:** 83.

**PREVALENCE RATE (indicated period: point, 12-month, lifetime)**

**PREVALENCE RATE (12-month):** 2.22 per 1,000 inhabitants. **STANDARD ERROR:** 2.00 **CI: LOW:** 2.60 **UPPER:** 4.00

**MEN:** 3.60 per 1,000 inhabitants. **STANDARD ERROR: CI: LOW: UPPER:**

**WOMEN:** 2.90 per 1,000 inhabitants. **STANDARD ERROR: CI: LOW: UPPER:**

**===============================================================================================**

**OBSERVATIONS:**

**REFERENCE NUMBER:** 68

**AUTHORS:** Ran M, Xiang M, Li S, Shan Y, Huang M, Li S, Liu Z, Chen E and Chan C.

**YEAR OF PUBLICATION:** 2003.

**TITLE:** Prevalence and course of schizophrenia in a Chinese rural area.

**JOURNAL:** *Australian & New Zealand Journal of Psychiatry, 37*(4), 452-457.

**===============================================================================================**

**STUDY COUNTRY:** China. **STUDY YEAR:** 1994.

**CASE FINDING SETTING:** census, general population.

**SAMPLING:** simple random.

**If sampling, indicate Clusters: CL1-CL2-P/CL:**

**NUMBER OF STAGES:** 2.

**SCREENING INSTRUMENT:**

**RESPONSE % PHASE 1: RESPONSE % PHASE 2:**

**DIAGNOSTIC INSTRUMENT:** clinical.

**CLASSIFICATION OF DISEASES:** ICD-10.

**DIAGNOSTIC CATEGORIES:** schizophrenia.

**STUDY AREA:** Chengdu. **POPULATION SIZE (denominator):** 89,512.

**AGE RANGE: LOWER:** 15. **UPPER:** 90.

**STUDY QUALITY:** 14.

**===============================================================================================**

**NUMBER OF ESTIMATES:** 367.

**PREVALENCE RATE (indicated period: point, 12-month, lifetime)**

**PREVALENCE RATE (Lifetime):** 4.10 per 1,000 inhabitants. **STANDARD ERROR:** **CI: LOW:** **UPPER:**

**MEN:** 3.80 per 1,000 inhabitants. **STANDARD ERROR: CI: LOW: UPPER:**

**WOMEN:** 4.30 per 1,000 inhabitants. **STANDARD ERROR: CI: LOW: UPPER:**

**===============================================================================================**

**OBSERVATIONS:**

**REFERENCE NUMBER:** 69

**AUTHORS:** Roca M, Gili M, Ferrer V, Bernardo M, Montaño JJ, Salvà JJ, Flores I and Leal S.

**YEAR OF PUBLICATION:** 1999.

**TITLE:** Mental disorders on the island of Formentera: prevalence in general population using the Schedules for Clinical Assessment in Neuropsychiatry (SCAN).

**JOURNAL:** *Social Psychiatry and Psychiatric Epidemiology, 34*(8), 410-415.

**===============================================================================================**

**STUDY COUNTRY:** Spain. **STUDY YEAR:** 1995.

**CASE FINDING SETTING:** census, general population.

**SAMPLING:** cluster.

**If sampling, indicate Clusters: CL1-CL2-P/CL:** CL2

**NUMBER OF STAGES:** 2.

**SCREENING INSTRUMENT:**

**RESPONSE % PHASE 1: RESPONSE % PHASE 2:**

**DIAGNOSTIC INSTRUMENT:** SCAN (Schedules for Clinical Assessment in Neuropsychiatry).

**CLASSIFICATION OF DISEASES:** ICD-10.

**DIAGNOSTIC CATEGORIES:** Schizophrenia and related disorders .

**STUDY AREA:** the island of Formentera. **POPULATION SIZE (denominator):** 697.

**AGE RANGE: LOWER:** 15. **UPPER:** 90.

**STUDY QUALITY:** 9.

**===============================================================================================**

**NUMBER OF ESTIMATES:** 3.

**PREVALENCE RATE (indicated period: point, 12-month, lifetime)**

**PREVALENCE RATE (Point):** 5.00 per 1,000 inhabitants. **STANDARD ERROR:** **CI: LOW:** **UPPER:**

**MEN:** **STANDARD ERROR: CI: LOW: UPPER:**

**WOMEN:** **STANDARD ERROR: CI: LOW: UPPER:**

**===============================================================================================**

**OBSERVATIONS:**

**REFERENCE NUMBER:** 70

**AUTHORS:** Ruggeri M, Leese M, Thornicroft G, Bisoffi G and Tansella M.

**YEAR OF PUBLICATION:** 2000.

**TITLE:** Definition and prevalence of severe and persistent mental illness.

**JOURNAL:** *The British Journal of Psychiatry, 177,* 149-155.

**===============================================================================================**

**STUDY COUNTRY:** Italy. **STUDY YEAR:** 1998.

**CASE FINDING SETTING:** attended population in Mental Health Services.

**SAMPLING:** simple random.

**If sampling, indicate Clusters: CL1-CL2-P/CL:**

**NUMBER OF STAGES:** 1.

**SCREENING INSTRUMENT:**

**RESPONSE % PHASE 1: RESPONSE % PHASE 2:**

**DIAGNOSTIC INSTRUMENT:** clinical.

**CLASSIFICATION OF DISEASES:** ICD-10.

**DIAGNOSTIC CATEGORIES:** non-affective psychoses.

**STUDY AREA:** Verona. **POPULATION SIZE (denominator):** 62,240.

**AGE RANGE: LOWER:** 18. **UPPER:** 90.

**STUDY QUALITY:** 13.

**===============================================================================================**

**NUMBER OF ESTIMATES:** 212.

**PREVALENCE RATE (indicated period: point, 12-month, lifetime)**

**PREVALENCE RATE (12-month):** 3.41 per 1,000 inhabitants. **STANDARD ERROR:** **CI: LOW:** 2.79 **UPPER:** 4.02

**MEN:** **STANDARD ERROR: CI: LOW: UPPER:**

**WOMEN:** **STANDARD ERROR: CI: LOW: UPPER:**

**===============================================================================================**

**OBSERVATIONS:**

**REFERENCE NUMBER:** 71

**AUTHORS:** Schrier AC, van de Wetering BJ, Mulder PG and Selten JP.

**YEAR OF PUBLICATION:** 2001.

**TITLE:** Point prevalence of schizophrenia in immigrant groups in Rotterdam: data from outpatient facilities.

**JOURNAL:** *European Psychiatry, 16*(3), 162–166.

**===============================================================================================**

**STUDY COUNTRY:** Netherlands. **STUDY YEAR:** 1994.

**CASE FINDING SETTING:** attended population in Mental Health Services + Primary Care + Social Services.

**SAMPLING:** nonrandom.

**If sampling, indicate Clusters: CL1-CL2-P/CL:**

**NUMBER OF STAGES:** 1.

**SCREENING INSTRUMENT:**

**RESPONSE % PHASE 1: RESPONSE % PHASE 2:**

**DIAGNOSTIC INSTRUMENT:** clinical.

**CLASSIFICATION OF DISEASES:** DSM-III-R.

**DIAGNOSTIC CATEGORIES:** schizophrenia.

**STUDY AREA:** Rotterdam. **POPULATION SIZE (denominator):** 337,362.

**AGE RANGE: LOWER:** 20. **UPPER:** 64.

**STUDY QUALITY:** 9.

**===============================================================================================**

**NUMBER OF ESTIMATES:** 713.

**PREVALENCE RATE (indicated period: point, 12-month, lifetime)**

**PREVALENCE RATE (Point):** 2.11 per 1,000 inhabitants. **STANDARD ERROR:** **CI: LOW:** **UPPER:**

**MEN:** 2.60 per 1,000 inhabitants. **STANDARD ERROR: CI: LOW: UPPER:**

**WOMEN:** 1.60 per 1,000 inhabitants. **STANDARD ERROR: CI: LOW: UPPER:**

**===============================================================================================**

**OBSERVATIONS:**

**REFERENCE NUMBER:** 72

**AUTHORS:** Scully PJ, Owens JM, Kinsella A and Waddington JL.

**YEAR OF PUBLICATION:** 2004.

**TITLE:** Schizophrenia, schizoaffective and bipolar disorder within an epidemiologically complete, homogeneous population in rural Ireland: small area variation in rate.

**JOURNAL:** *Schizophrenia Research, 67*(2-3), 143-155.

**===============================================================================================**

**STUDY COUNTRY:** Ireland. **STUDY YEAR:** 1996.

**CASE FINDING SETTING:** attended population in Mental Health Services + Primary Care + Social Services.

**SAMPLING:** simple random.

**If sampling, indicate Clusters: CL1-CL2-P/CL:**

**NUMBER OF STAGES:** 1.

**SCREENING INSTRUMENT:**

**RESPONSE % PHASE 1: RESPONSE % PHASE 2:**

**DIAGNOSTIC INSTRUMENT:** SCID (Structure Clinical Interview for DSM-IV).

**CLASSIFICATION OF DISEASES:** DSM-III-R.

**DIAGNOSTIC CATEGORIES:** schizophrenia.

**STUDY AREA:** Ireland.  **POPULATION SIZE (denominator):** 29,542.

**AGE RANGE: LOWER:** 0. **UPPER:** 90.

**STUDY QUALITY:** 15.

**===============================================================================================**

**NUMBER OF ESTIMATES:** 115.

**PREVALENCE RATE (indicated period: point, 12-month, lifetime)**

**PREVALENCE RATE (Lifetime):** 3.59 per 1,000 inhabitants. **STANDARD ERROR:** 0.40 **CI: LOW:** **UPPER:**

**MEN:** 4.00 per 1,000 inhabitants. **STANDARD ERROR: CI: LOW: UPPER:**

**WOMEN:** 3.80 per 1,000 inhabitants. **STANDARD ERROR: CI: LOW: UPPER:**

**===============================================================================================**

**OBSERVATIONS:**

**REFERENCE NUMBER:** 73

**AUTHORS:** Shivashankar S, Telfer S, Arunagiriraj J, McKinnon M, Jauhar S, Krishnadas R and McCreadie R.

**YEAR OF PUBLICATION:** 2013.

**TITLE:** Has the prevalence, clinical presentation and social functioning of schizophrenia changed over the last 25 years? Nithsdale schizophrenia survey revisited.

**JOURNAL:** *Schizophrenia Research, 146*(1-3), 349–356.

**===============================================================================================**

**STUDY COUNTRY:** Scotland. **STUDY YEAR:** 2006.

**CASE FINDING SETTING:** census, general population.

**SAMPLING:** simple random.

**If sampling, indicate Clusters: CL1-CL2-P/CL:**

**NUMBER OF STAGES:** 1.

**SCREENING INSTRUMENT:**

**RESPONSE % PHASE 1:** 64%. **RESPONSE % PHASE 2:**

**DIAGNOSTIC INSTRUMENT:** clinical.

**CLASSIFICATION OF DISEASES:** ICD-10.

**DIAGNOSTIC CATEGORIES:** schizophrenia.

**STUDY AREA:** Nithsdale.  **POPULATION SIZE (denominator):** 205.

**AGE RANGE: LOWER:** 34. **UPPER:** 68.

**STUDY QUALITY:** 8.

**===============================================================================================**

**NUMBER OF ESTIMATES:** 1.

**PREVALENCE RATE (indicated period: point, 12-month, lifetime)**

**PREVALENCE RATE (Point):** 4.88 per 1,000 inhabitants. **STANDARD ERROR:** **CI: LOW:**  **UPPER:**

**MEN:** **STANDARD ERROR: CI: LOW: UPPER:**

**WOMEN:** **STANDARD ERROR: CI: LOW: UPPER:**

**===============================================================================================**

**OBSERVATIONS:**

**REFERENCE NUMBER:** 74

**AUTHORS:** Singleton N, Bumpstead R, O'Brien M, Lee A and Meltzer H.

**YEAR OF PUBLICATION:** 2003.

**TITLE:** Psychiatric morbidity among adults living in private households, 2000.

**JOURNAL:** *International Review of Psychiatry, 15*(1-2), 65-73.

**===============================================================================================**

**STUDY COUNTRY:** United Kingdom. **STUDY YEAR:** 2000.

**CASE FINDING SETTING:** census, general population.

**SAMPLING:** nonrandom.

**If sampling, indicate Clusters: CL1-CL2-P/CL:**

**NUMBER OF STAGES:** 2.

**SCREENING INSTRUMENT:**

**RESPONSE % PHASE 1: RESPONSE % PHASE 2:**

**DIAGNOSTIC INSTRUMENT:** SCAN (Schedules for Clinical Assessment in Neuropsychiatry).

**CLASSIFICATION OF DISEASES:** DSM-IV.

**DIAGNOSTIC CATEGORIES:** probable psychotic disorder.

**STUDY AREA:** Great Britain.  **POPULATION SIZE (denominator):** 8,886.

**AGE RANGE: LOWER:** 16. **UPPER:** 74.

**STUDY QUALITY:** 13.

**===============================================================================================**

**NUMBER OF ESTIMATES:** 44.

**PREVALENCE RATE (indicated period: point, 12-month, lifetime)**

**PREVALENCE RATE (12-month):** 4.95 per 1,000 inhabitants. **STANDARD ERROR:** **CI: LOW:**  **UPPER:**

**MEN:** 6.00 per 1,000 inhabitants. **STANDARD ERROR: CI: LOW: UPPER:**

**WOMEN:** 5.00 per 1,000 inhabitants. **STANDARD ERROR: CI: LOW: UPPER:**

**===============================================================================================**

**OBSERVATIONS:**

**REFERENCE NUMBER:** 75

**AUTHORS:** Suvisaari J, Pera J, Saarni S, Juvonen H, Tuulio-Henriksson A and Lonnqvist J.

**YEAR OF PUBLICATION:** 2009.

**TITLE:** The Epidemiology and Descriptive and Predictive Validity of DSM-IV Delusional Disorder and Subtypes of Schizophrenia.

**JOURNAL:** *Clinical Schizophrenia and Related Psychoses, 2*(4), 289–97.

**===============================================================================================**

**STUDY COUNTRY:** Finland. **STUDY YEAR:** 2001.

**CASE FINDING SETTING:** census, general population.

**SAMPLING:** clusters.

**If sampling, indicate Clusters: CL1-CL2-P/CL:** CL2

**NUMBER OF STAGES:** 2.

**SCREENING INSTRUMENT:** M-CIDI (Composite International Diagnostic Interview).

**RESPONSE % PHASE 1:** 94%. **RESPONSE % PHASE 2:**

**DIAGNOSTIC INSTRUMENT:** SCID-I (Structure Clinical Interview for DSM-IV).

**CLASSIFICATION OF DISEASES:** DSM-IV.

**DIAGNOSTIC CATEGORIES:** schizophrenia (paranoid, disorganized and undifferentiated subtypes).

**STUDY AREA:** Finland.  **POPULATION SIZE (denominator):** 8,028.

**AGE RANGE: LOWER:** 30. **UPPER:** Unlimited.

**STUDY QUALITY:** 11.

**===============================================================================================**

**NUMBER OF ESTIMATES:** 66 schizophrenia: 13 (paranoid) + 19 (disorganized) + 34 (undifferentiated).

**PREVALENCE RATE (indicated period: point, 12-month, lifetime)**

**PREVALENCE RATE (Lifetime):** 8.22 per 1,000 inhabitants. **STANDARD ERROR:** **CI: LOW:** 0.74 **UPPER:** 2.50

**PREVALENCE RATE (L. paranoid):** 2.40 per 1,000 inhabitants. **STANDARD ERROR:** **CI: LOW:** 1.50 **UPPER:** 3.70

**PREVALENCE RATE (L. disorganized):** 1.60 per 1,000 inhabitants. **STANDARD ERROR:** **CI: LOW:** 0.90 **UPPER:** 2.70

**PREVALENCE RATE (L. undifferentiated):** 4.20 per 1,000 inhabitants.**STANDARD ERROR:** **CI: LOW:** 3.00 **UPPER:** 5.90

**MEN:** **STANDARD ERROR: CI: LOW: UPPER:**

**WOMEN:** **STANDARD ERROR: CI: LOW: UPPER:**

**===============================================================================================**

**OBSERVATIONS:**

**REFERENCE NUMBER:** 76

**AUTHORS:** Thornicroft G, Strathdee G, Phelan M, Holloway F, Wykes T, Dunn G, Mccrone P, Leese M, Johnson S and Szmukler G.

**YEAR OF PUBLICATION:** 1998.

**TITLE:** Rationale and design. PRISM Psychosis Study I.

**JOURNAL:** *The British Journal of Psychiatry, 173*(11), 363-370.

**===============================================================================================**

**STUDY COUNTRY:** United Kingdom. **STUDY YEAR:** 1992.

**CASE FINDING SETTING:** attended population in Mental Health Services + Primary Care + Social Services.

**SAMPLING:** simple random.

**If sampling, indicate Clusters: CL1-CL2-P/CL:**

**NUMBER OF STAGES:** 2.

**SCREENING INSTRUMENT:**

**RESPONSE % PHASE 1: RESPONSE % PHASE 2:**

**DIAGNOSTIC INSTRUMENT:** SCAN (Schedules for Clinical Assessment in Neuropsychiatry).

**CLASSIFICATION OF DISEASES:** ICD-10.

**DIAGNOSTIC CATEGORIES:** non-affective psychoses.

**STUDY AREA:** United Kingdom. **POPULATION SIZE (denominator):** 80,285.

**AGE RANGE: LOWER:** 15. **UPPER:** 85.

**STUDY QUALITY:** 13.

**===============================================================================================**

**NUMBER OF ESTIMATES:** 618.

**PREVALENCE RATE (indicated period: point, 12-month, lifetime)**

**PREVALENCE RATE (12-month):** 7.70 per 1,000 inhabitants. **STANDARD ERROR:** **CI: LOW:** 7.10  **UPPER:** 8.40

**MEN:** **STANDARD ERROR: CI: LOW: UPPER:**

**WOMEN:** **STANDARD ERROR: CI: LOW: UPPER:**

**===============================================================================================**

**OBSERVATIONS:**

**REFERENCE NUMBER:** 77

**AUTHORS:** Tizón J, Ferrando J, Parés A, Artigué J, Parra B and Pérez C.

**YEAR OF PUBLICATION:** 2007.

**TITLE:** Schizophrenic disorders in primary care mental health.

**JOURNAL:** *Atención Primaria, 39*(3), 119-26.

**===============================================================================================**

**STUDY COUNTRY:** Spain. **STUDY YEAR:** 2000.

**CASE FINDING SETTING:** attended population in Mental Health Services + Primary Care.

**SAMPLING:** simple random.

**If sampling, indicate Clusters: CL1-CL2-P/CL:**

**NUMBER OF STAGES:** 1.

**SCREENING INSTRUMENT:**

**RESPONSE % PHASE 1: RESPONSE % PHASE 2:**

**DIAGNOSTIC INSTRUMENT:** clinical.

**CLASSIFICATION OF DISEASES:** DSM-IV.

**DIAGNOSTIC CATEGORIES:** Schizophrenia and related disorders.

**STUDY AREA:** Spain. **POPULATION SIZE (denominator):** 21,236.

**AGE RANGE: LOWER:** 15. **UPPER:** 90.

**STUDY QUALITY:** 11.

**===============================================================================================**

**NUMBER OF ESTIMATES:** 97.

**PREVALENCE RATE (indicated period: point, 12-month, lifetime)**

**PREVALENCE RATE (12-month):** 4.57 per 1,000 inhabitants. **STANDARD ERROR:** **CI: LOW:** 4.18  **UPPER:** 5.01

**MEN:** **STANDARD ERROR: CI: LOW: UPPER:**

**WOMEN:** **STANDARD ERROR: CI: LOW: UPPER:**

**===============================================================================================**

**OBSERVATIONS:**

**REFERENCE NUMBER:** 78

**AUTHORS:** Tizón J, Ferrando J, Artigue J, Parra B, Parés A, Gomà M, Pérez C, Pareja F, Sorribes M, Marzari B, Quijada Y and Català L.

**YEAR OF PUBLICATION:** 2009.

**TITLE:** Neighborhood differences in psychoses: Prevalence of psychotic disorders in two socially-differentiated metropolitan areas of Barcelona.

**JOURNAL:** *Schizophrenia Research, 112*(1-3), 143-148.

**===============================================================================================**

**STUDY COUNTRY:** Spain.  **STUDY YEAR:** 2000.

**CASE FINDING SETTING:** attended population in Mental Health Services + Primary Care.

**SAMPLING:** nonrandom.

**If sampling, indicate Clusters: CL1-CL2-P/CL:**

**NUMBER OF STAGES:** 1.

**SCREENING INSTRUMENT:**

**RESPONSE % PHASE 1: RESPONSE % PHASE 2:**

**DIAGNOSTIC INSTRUMENT:** clinical.

**CLASSIFICATION OF DISEASES:** DSM-IV.

**DIAGNOSTIC CATEGORIES:** Schizophrenia and related disorders .

**STUDY AREA:** Barcelona (Verneda and Mina). **POPULATION SIZE (denominator):** 103,615.

**AGE RANGE: LOWER:** 15. **UPPER:** 54.

**STUDY QUALITY:** 9.

**===============================================================================================**

**NUMBER OF ESTIMATES:** 477 of the total population.

**PREVALENCE RATE (indicated period: point, 12-month, lifetime)**

**PREVALENCE RATE (Lifetime):** 4.60 per 1,000 inhabitants. **STANDARD ERROR:** **CI: LOW:** 4.19 **UPPER:** 5.02

**MEN:** **STANDARD ERROR: CI: LOW: UPPER:**

**WOMEN:** **STANDARD ERROR: CI: LOW: UPPER:**

**===============================================================================================**

**OBSERVATIONS:**

**REFERENCE NUMBER:** 79

**AUTHORS:** Vanasse A, Courteau J, Fleury MJ, Grégoire JP, Lesage A and Moisan J.

**YEAR OF PUBLICATION:** 2012.

**TITLE:** Treatment prevalence and incidence of schizophrenia in Quebec using a population health services perspective: different algorithms, different estimates.

**JOURNAL:** *Social Psychiatry and Psychiatric Epidemiology, 47,* 533-543.

**===============================================================================================**

**STUDY COUNTRY:** Canada. **STUDY YEAR:** 2006.

**CASE FINDING SETTING:** attended population in Mental Health Services + Primary Care.

**SAMPLING:** nonrandom.

**If sampling, indicate Clusters: CL1-CL2-P/CL:**

**NUMBER OF STAGES:** 1.

**SCREENING INSTRUMENT:**

**RESPONSE % PHASE 1: RESPONSE % PHASE 2:**

**DIAGNOSTIC INSTRUMENT:** clinical.

**CLASSIFICATION OF DISEASES:** ICD-10.

**DIAGNOSTIC CATEGORIES:** schizophrenia.

**STUDY AREA:** Quebec. **POPULATION SIZE (denominator):** 5,996,925.

**AGE RANGE: LOWER:** 18. **UPPER:** Unlimited.

**STUDY QUALITY:** 9.

**===============================================================================================**

**NUMBER OF ESTIMATES:** 35,585 (lifetime) and 7,988 (12-month).

**PREVALENCE RATE (indicated period: point, 12-month, lifetime)**

**PREVALENCE RATE (Lifetime):** 5.93 per 1,000 inhabitants. **STANDARD ERROR:**

**CI: LOW:** 5.00-15.70. **UPPER:** 5.20-16.00.

**PREVALENCE RATE (12-month):** 1.33 per 1,000 inhabitants. **STANDARD ERROR:**

**CI: LOW:** 1.00-6.60. **UPPER:** 1.10-6.80.

**MEN:** **STANDARD ERROR: CI: LOW: UPPER:**

**WOMEN:** **STANDARD ERROR: CI: LOW: UPPER:**

**===============================================================================================**

**OBSERVATIONS:**

**REFERENCE NUMBER:** 80

**AUTHORS:** Vicente B, Kohn R, Rioseco P, Saldivia S, Baker C and Torres S.

**YEAR OF PUBLICATION:** 2004.

**TITLE:** Psychiatric Disorders among the Mapuche in Chile.

**JOURNAL:** *The British Journal of Psychiatry, 184,* 299-305.

**===============================================================================================**

**STUDY COUNTRY:** Chile. **STUDY YEAR:** 1999.

**CASE FINDING SETTING:** census, general population.

**SAMPLING:** cluster.

**If sampling, indicate Clusters: CL1-CL2-P/CL:** CL1

**NUMBER OF STAGES:** 1.

**SCREENING INSTRUMENT:**

**RESPONSE % PHASE 1: RESPONSE % PHASE 2:**

**DIAGNOSTIC INSTRUMENT:** CIDI (Composite International Diagnostic Interview).

**CLASSIFICATION OF DISEASES:** DSM-III-R.

**DIAGNOSTIC CATEGORIES:** non-affective psychoses.

**STUDY AREA:** Chile.  **POPULATION SIZE (denominator):** 2,978.

**AGE RANGE: LOWER:** 15. **UPPER:** 90.

**STUDY QUALITY:** 13.

**===============================================================================================**

**NUMBER OF ESTIMATES:** 15.

**PREVALENCE RATE (indicated period: point, 12-month, lifetime)**

**PREVALENCE RATE (Point):** 5.04 per 1,000 inhabitants. **STANDARD ERROR:** 1.00 **CI: LOW: UPPER:**

**PREVALENCE RATE (12-month):** 5.04 per 1,000 inhabitants. **STANDARD ERROR:** 1.00 **CI: LOW:** **UPPER:**

**MEN:** 2.00 per 1,000 inhabitants. **STANDARD ERROR: CI: LOW: UPPER:**

**WOMEN:** 2.00 per 1,000 inhabitants. **STANDARD ERROR: CI: LOW: UPPER:**

**===============================================================================================**

**OBSERVATIONS:**

**REFERENCE NUMBER:** 81

**AUTHORS:** Vicente B, Kohn R, Rioseco P, Saldivia S, Levav I and Torres S.

**YEAR OF PUBLICATION:** 2006.

**TITLE:** Lifetime and 12-month prevalence of DSM-III-R disorders in the Chile psychiatric prevalence study.

**JOURNAL:** *American Journal of Psychiatry, 163*(8), 1362-1370.

**===============================================================================================**

**STUDY COUNTRY:** Chile. **STUDY YEAR:** 1999.

**CASE FINDING SETTING:** census, general population.

**SAMPLING:** nonrandom.

**If sampling, indicate Clusters: CL1-CL2-P/CL:**

**NUMBER OF STAGES:** 1.

**SCREENING INSTRUMENT:**

**RESPONSE % PHASE 1: RESPONSE % PHASE 2:**

**DIAGNOSTIC INSTRUMENT:** CIDI (Composite International Diagnostic Interview).

**CLASSIFICATION OF DISEASES:** DSM-III-R.

**DIAGNOSTIC CATEGORIES:** non-affective psychoses.

**STUDY AREA:** The Mapuche.  **POPULATION SIZE (denominator):** 2,978.

**AGE RANGE: LOWER:** 15. **UPPER:** 90.

**STUDY QUALITY:** 13.

**===============================================================================================**

**NUMBER OF ESTIMATES:** 21 (12-month) + 54 (lifetime).

**PREVALENCE RATE (indicated period: point, 12-month, lifetime)**

**PREVALENCE RATE (12-month):** 7.05 per 1,000 inhabitants. **STANDARD ERROR:** 2.00 **CI: LOW: UPPER:**

**PREVALENCE RATE (Lifetime):** 18.13 per 1,000 inhabitants. **STANDARD ERROR:** 3.00 **CI: LOW:** **UPPER:**

**MEN:** 2.00 (12-month) / 16.00 (lifetime) per 1,000 inhabitants. **STANDARD ERROR: CI: LOW: UPPER:**

**WOMEN:** 11.00 (12-month) / 19.00 (lifetime) per 1,000 inhabitants. **STANDARD ERROR: CI: LOW: UPPER:**

**===============================================================================================**

**OBSERVATIONS:**

**REFERENCE NUMBER:** 82

**AUTHORS:** Villaverde ML, Gracia R, de la Fuente J, González de Rivera JL and Rodríguez-Pulido F.

**YEAR OF PUBLICATION:** 1993.

**TITLE:** Estudio comunitario de salud mental en población urbana de Tenerife.

**JOURNAL:** *El método epidemiológico en salud mental* (pp. 187-214). Barcelona: Masson-Salvat.

**===============================================================================================**

**STUDY COUNTRY:** Spain. **STUDY YEAR:** 1990.

**CASE FINDING SETTING:** census, general population.

**SAMPLING:** cluster.

**If sampling, indicate Clusters: CL1-CL2-P/CL:** CL2

**NUMBER OF STAGES:** 2.

**SCREENING INSTRUMENT:** General Health Questionnaire (GHQ-28).

**RESPONSE % PHASE 1: RESPONSE % PHASE 2:**

**DIAGNOSTIC INSTRUMENT:** CIS (Clinical Interview Schedule).

**CLASSIFICATION OF DISEASES:** DSM-III-R.

**DIAGNOSTIC CATEGORIES:** schizophrenia.

**STUDY AREA:** Spain. **POPULATION SIZE (denominator):** 660.

**AGE RANGE: LOWER:** 15. **UPPER:** 90.

**STUDY QUALITY:** 13.

**===============================================================================================**

**NUMBER OF ESTIMATES:** 4.

**PREVALENCE RATE (indicated period: point, 12-month, lifetime)**

**PREVALENCE RATE (Point):** 6.06 per 1,000 inhabitants. **STANDARD ERROR:** **CI: LOW: UPPER:**

**MEN:** **STANDARD ERROR: CI: LOW: UPPER:**

**WOMEN:** **STANDARD ERROR: CI: LOW: UPPER:**

**===============================================================================================**

**OBSERVATIONS:**

**REFERENCE NUMBER:** 83

**AUTHORS:** Waldo MC.

**YEAR OF PUBLICATION:** 1999.

**TITLE:** Schizophrenia in Kosrae, Micronesia: Prevalence, gender ratios, and clinical symptomatology.

**JOURNAL:** *Schizophrenia Research, 35*, 175-181.

**===============================================================================================**

**STUDY COUNTRY:** Micronesia. **STUDY YEAR:** 1997.

**CASE FINDING SETTING:** attended population in Mental Health Services + Primary Care + Social Services.

**SAMPLING:** simple random.

**If sampling, indicate Clusters: CL1-CL2-P/CL:**

**NUMBER OF STAGES:** 1.

**SCREENING INSTRUMENT:**

**RESPONSE % PHASE 1: RESPONSE % PHASE 2:**

**DIAGNOSTIC INSTRUMENT:** SCID (Structure Clinical Interview for DSM-IV).

**CLASSIFICATION OF DISEASES:** DSM-IV.

**DIAGNOSTIC CATEGORIES:** schizophrenia.

**STUDY AREA:** Kosrae.  **POPULATION SIZE (denominator):** 3,235.

**AGE RANGE: LOWER:** 15. **UPPER:** 90.

**STUDY QUALITY:** 10.

**===============================================================================================**

**NUMBER OF ESTIMATES:** 22.

**PREVALENCE RATE (indicated period: point, 12-month, lifetime)**

**PREVALENCE RATE (Point):** 6.80 per 1,000 inhabitants. **STANDARD ERROR:** **CI: LOW: UPPER:**

**MEN:** **STANDARD ERROR: CI: LOW: UPPER:**

**WOMEN:** **STANDARD ERROR: CI: LOW: UPPER:**

**===============================================================================================**

**OBSERVATIONS:**

**REFERENCE NUMBER:** 84

**AUTHORS:** Widerlöv B, Lindström E and von Knorring L.

**YEAR OF PUBLICATION:** 1997.

**TITLE:** One-year prevalence of long-term functional psychosis in three different areas of Uppsala.

**JOURNAL:** *Acta Psychiatrica Scandinavica, 96*(6), 452-458.

**===============================================================================================**

**STUDY COUNTRY:** Sweden. **STUDY YEAR:** 1991.

**CASE FINDING SETTING:** attended population in Mental Health Services + Primary Care + Social Services.

**SAMPLING:** simple random.

**If sampling, indicate Clusters: CL1-CL2-P/CL:**

**NUMBER OF STAGES:** 1.

**SCREENING INSTRUMENT:**

**RESPONSE % PHASE 1: RESPONSE % PHASE 2:**

**DIAGNOSTIC INSTRUMENT:** clinical.

**CLASSIFICATION OF DISEASES:** DSM-III-R.

**DIAGNOSTIC CATEGORIES:** schizophrenia.

**STUDY AREA:** Sweden.  **POPULATION SIZE (denominator):** 64,886.

**AGE RANGE: LOWER:** 18. **UPPER:** 90.

**STUDY QUALITY:** 11.

**===============================================================================================**

**NUMBER OF ESTIMATES:** 273.

**PREVALENCE RATE (indicated period: point, 12-month, lifetime)**

**PREVALENCE RATE (12-month):** 4.21 per 1,000 inhabitants. **STANDARD ERROR:** **CI: LOW: UPPER:**

**MEN:** **STANDARD ERROR: CI: LOW: UPPER:**

**WOMEN:** **STANDARD ERROR: CI: LOW: UPPER:**

**===============================================================================================**

**OBSERVATIONS:**

**REFERENCE NUMBER:** 85

**AUTHORS:** Wu EQ, Shi L, Birnbaum H, Hudson T and Kessler R.

**YEAR OF PUBLICATION:** 2006.

**TITLE:** Annual prevalence of diagnosed schizophrenia in the USA: a claims data analysis approach.

**JOURNAL:** *Psychological Medicine, 36,* 1535-1540.

**===============================================================================================**

**STUDY COUNTRY:** USA. **STUDY YEAR:** 2002.

**CASE FINDING SETTING:** attended population in Mental Health Services + Primary Care.

**SAMPLING:** nonrandom.

**If sampling, indicate Clusters: CL1-CL2-P/CL:**

**NUMBER OF STAGES:** 1.

**SCREENING INSTRUMENT:**

**RESPONSE % PHASE 1: RESPONSE % PHASE 2:**

**DIAGNOSTIC INSTRUMENT:** clinical.

**CLASSIFICATION OF DISEASES:** ICD-9.

**DIAGNOSTIC CATEGORIES:** schizophrenia.

**STUDY AREA:** USA.  **POPULATION SIZE (denominator):** 6,800,000.

**AGE RANGE: LOWER:** 18. **UPPER:** 65.

**STUDY QUALITY:** 7.

**===============================================================================================**

**NUMBER OF ESTIMATES:** 34,680.

**PREVALENCE RATE (indicated period: point, 12-month, lifetime)**

**PREVALENCE RATE (12-month):** 5.10 per 1,000 inhabitants. **STANDARD ERROR:** **CI: LOW: UPPER:**

**MEN:** 5.40 per 1,000 inhabitants. **STANDARD ERROR: CI: LOW: UPPER:**

**WOMEN:** 4.90 per 1,000 inhabitants. **STANDARD ERROR: CI: LOW: UPPER:**

**===============================================================================================**

**OBSERVATIONS:**

**REFERENCE NUMBER:** 86

**AUTHORS:** Xiang Y, Ma X, Cai Z, Li S, Xiang Y, Guo H, Hou Y, Li Z, Li Z, Tao Y, Dang W, Wu X, Deng J, Lai K and Ungvari G.

**YEAR OF PUBLICATION:** 2008.

**TITLE:** Prevalence and socio-demographic correlates of schizophrenia in Beijing, China.

**JOURNAL:** *Schizophrenia Research, 102*(1-3), 270-277.

**===============================================================================================**

**STUDY COUNTRY:** China. **STUDY YEAR:** 2003.

**CASE FINDING SETTING:** census, general population.

**SAMPLING:** cluster.

**If sampling, indicate Clusters: CL1-CL2-P/CL:** CL1

**NUMBER OF STAGES:** 1.

**SCREENING INSTRUMENT:**

**RESPONSE % PHASE 1: RESPONSE % PHASE 2:**

**DIAGNOSTIC INSTRUMENT:** CIDI (Composite International Diagnostic Interview).

**CLASSIFICATION OF DISEASES:** ICD-10.

**DIAGNOSTIC CATEGORIES:** schizophrenia.

**STUDY AREA:** Beijing.  **POPULATION SIZE (denominator):** 5,926.

**AGE RANGE: LOWER:** 15. **UPPER:** 90.

**STUDY QUALITY:** 15.

**===============================================================================================**

**NUMBER OF ESTIMATES:** 29.

**PREVALENCE RATE (indicated period: point, 12-month, lifetime)**

**PREVALENCE RATE (Lifetime):** 4.89 per 1,000 inhabitants. **STANDARD ERROR:** **CI: LOW:** 3.00 **UPPER:** 6.80

**MEN:** 4.40 per 1,000 inhabitants. **STANDARD ERROR: CI: LOW: UPPER:**

**WOMEN:** 5.50 per 1,000 inhabitants. **STANDARD ERROR: CI: LOW: UPPER:**

**===============================================================================================**

**OBSERVATIONS:**

**REFERENCE NUMBER:** 87

**AUTHORS:** Yang JZ, Kang C, Zeng Y, Li J, Li P, Wan W, Zhao X, Guo W, Xu X, Yang X, Li Q, Liu X and Pauline S.

**YEAR OF PUBLICATION:** 2014.

**TITLE:** Prevalence and prognosis of schizophrenia in Jinuo people in China: A prospective 30-year follow-up study.

**JOURNAL:** *International Journal of Social Psychiatry, 60*(5), 482-488.

**===============================================================================================**

**STUDY COUNTRY:** China. **STUDY YEAR:** 2009.

**CASE FINDING SETTING:** census, general population.

**SAMPLING:** clusters.

**If sampling, indicate Clusters: CL1-CL2-P/CL:** CL2

**NUMBER OF STAGES:** 2.

**SCREENING INSTRUMENT:**

**RESPONSE % PHASE 1: RESPONSE % PHASE 2:**

**DIAGNOSTIC INSTRUMENT:** CIDI (Composite International Diagnostic Interview).

**CLASSIFICATION OF DISEASES:** ICD-10.

**DIAGNOSTIC CATEGORIES:** schizophrenia.

**STUDY AREA:** Jinuo people.  **POPULATION SIZE (denominator):** 1,984.

**AGE RANGE: LOWER:** 18. **UPPER:** Unlimited.

**STUDY QUALITY:** 13.

**===============================================================================================**

**NUMBER OF ESTIMATES:** 5.

**PREVALENCE RATE (indicated period: point, 12-month, lifetime)**

**PREVALENCE RATE (Point):** 2.52 per 1,000 inhabitants. **STANDARD ERROR:** **CI: LOW: UPPER:**

**MEN:** 3.80 per 1,000 inhabitants. **STANDARD ERROR: CI: LOW: UPPER:**

**WOMEN:** 1.10 per 1,000 inhabitants. **STANDARD ERROR: CI: LOW: UPPER:**

**===============================================================================================**

**OBSERVATIONS:**

**REFERENCE NUMBER:** 88

**AUTHORS:** Youssef HA, Scully PJ, Kinsella A and Waddington JL.

**YEAR OF PUBLICATION:** 1999.

**TITLE:** Geographical variation in rate of schizophrenia in rural Ireland by place at birth vs place at onset.

**JOURNAL:** *Schizophrenia Research, 37*(3), 233-243.

**===============================================================================================**

**STUDY COUNTRY:** Ireland. **STUDY YEAR:** 1996.

**CASE FINDING SETTING:** attended population in Mental Health Care + Primary + Social Services.

**SAMPLING:** nonrandom.

**If sampling, indicate Clusters: CL1-CL2-P/CL:**

**NUMBER OF STAGES:** 1.

**SCREENING INSTRUMENT:**

**RESPONSE % PHASE 1: RESPONSE % PHASE 2:**

**DIAGNOSTIC INSTRUMENT:** clinical.

**CLASSIFICATION OF DISEASES:** DSM-III-R.

**DIAGNOSTIC CATEGORIES:** schizophrenia.

**STUDY AREA:** County Monaghan. **POPULATION SIZE (denominator):** 21,520.

**AGE RANGE: LOWER:** 15. **UPPER:** 44.

**STUDY QUALITY:** 11.

**===============================================================================================**

**NUMBER OF ESTIMATES:** 72.

**PREVALENCE RATE (12-month):** 3.39 per 1,000 inhabitants. **STANDARD ERROR:** 0.40 **CI: LOW: UPPER:**

**MEN:** 3.60 per 1,000 inhabitants. **STANDARD ERROR: CI: LOW: UPPER:**

**WOMEN:** 3.10 per 1,000 inhabitants. **STANDARD ERROR: CI: LOW: UPPER:**

**===============================================================================================**

**OBSERVATIONS:**
