# Supplementary material for: Prevalence of psychotic disorders and its association with methodological issues. A systematic review and meta-analyses
Source: PLoS One. 2018 Apr 12;13(4):e0195687. doi: 10.1371/journal.pone.0195687 (PMC5896987; doi:10.1371/journal.pone.0195687)
Supplement: S1 Table — (DOC) [file pone.0195687.s003.doc]

S1 Table: Variables used to characterize the prevalence articles

| Definitions used in tables | | |
| --- | --- | --- |
| **Heading** | **Description** | **Categories** |
| ID | Citations reference number |  |
| First author | Name of the first author of each article |  |
| Year | Year of publication of each article |  |
| Country | Name of the country of the study |  |
| 1.Case finding setting | Method of finding cases | - General population (census) - Attended population (mental health and/or social services) |
| 2.Method of confirming diagnosis | Method of determining ‘caseness’ | - CIDI (Composite International Diagnostic Interview) - SCAN (Schedules for Clinical Assessment in Neuropsychiatry) - SCID (Structure Clinical Interview for DSM-IV) - Clinical diagnosis (clinical judgment) - Others: CIS (Clinical Interview Schedule), DIGS (Diagnostic Interview for Genetic Studies), DISSI (Diagnostic Interview Schedule computer screening interview), DIP (Diagnostic Interview for Psychoses), MINI (Mini-International Neuropsychiatric Interview), OPCRIT (Operational Criteria Checklist for Psychosis), SADS (Schedule for Affective Disorders and Schizophrenia) |
| 3.International classification of diseases | Diagnostic criteria system used | - ICD (International Classification of Diseases: 7, 8, 9 and 10^th^ revision) - DSM (Diagnostic and Statistic Manual of Mental Disorders: III, III-R, IV and IV-TR) - Both |
| 4.Diagnostic categories | Specific diagnosis of cases in the study | - Schizophrenia (S) - Non-affective psychosis (NAP) - Schizophrenia and related disorders (SRD): schizophrenia, persistent delusional disorders, acute and transient psychotic disorders, schizoaffective disorders, schizotypal disorder, Induced delusional disorder - Probable psychotic disorder (Probable) |
| 5.Study Quality | To indicate the quality rating of each article through the Saha et al. Scale | Range 1-16 |
| 6.Type of period prevalence rates | The period of time used to estimate the prevalence reported | - Point:1 month or less - 12-months: Between 1 to 12 months - Lifetime: More than 12 months |
| Population size | Number of inhabitants in the area specified (denominator) |  |
| Cases | Number of cases estimate (numerator) |  |
| Prevalence rates | Prevalence measures the proportion of individuals who manifest a disorder at a specified time, or during a specified period.  Estimates are given as a uniform per 1,000 population |  |
