# Supplementary material for: Prevalence of psychotic disorders and its association with methodological issues. A systematic review and meta-analyses
Source: PLoS One. 2018 Apr 12;13(4):e0195687. doi: 10.1371/journal.pone.0195687 (PMC5896987; doi:10.1371/journal.pone.0195687)
Supplement: S2 Table — (DOC) [file pone.0195687.s004.doc]

**Table S2. Reporting Scale for Assessing Study Quality***

| **Items** | **Quality Score** |
| --- | --- |
| **Rate type** |  |
| Rate type mentioned | 1 |
| Rate type not mentioned | 0 |
| **Case Ascertainment** |  |
| Community survey or multiple institutions | 2 |
| Hospital inpatient & outpatients, case registers | 1 |
| Not specified | 0 |
| **Diagnosis** | |
| Any diagnostic system reported (eg., DSM, ICD, Not specified) | 1 |
| Own system / symptoms described | 0 |
| No system / not specified | 0 |
| **Method of diagnostic assignment** | |
| Diagnostic interview (face-to-face) | 3 |
| Case note review (standardized) | 2 |
| Clinical diagnosis (recorded in hospital notes or registries) | 1 |
| Unspecified | 0 |
| **Information on rates** | |
| Raw data-numerator | 1 |
| Raw data-denominator | 1 |
| Age and/or sex standardized | 1 |
| If age/sex standardized, method provided | 1 |
| Confidence Intervals | 1 |
| Numerator/denominator match in time | 1 |
| Numerator/denominator match in space | 1 |
| Additional 'merits' | |
| Text on inter-rater reliability | 1 |
| Leakage study | 1 |

* Range = 0-16

*From*: Saha S, Chant D, Welham J, McGrath J. A systematic review of the prevalence of schizophrenia. PLoS Med 2005;2 (5):413-433
